# Constraining uncertainty in the timescale of angiosperm evolution and the veracity of a Cretaceous Terrestrial Revolution

**DOI:** 10.1111/nph.15011

**Published:** 2018-02-05

**Authors:** Jose Barba‐Montoya, Mario dos Reis, Harald Schneider, Philip C. J. Donoghue, Ziheng Yang

**Affiliations:** ^1^ Department of Genetics, Evolution and Environment University College London Darwin Building Gower Street London WC1E 6BT UK; ^2^ School of Biological and Chemical Sciences Queen Mary University of London Mile End Road London E1 4NS UK; ^3^ Center of Integrative Conservation Xishuangbanna Tropical Botanical Garden Chinese Academy of Sciences Menglun Yunnan China; ^4^ Department of Botany Natural History Museum Cromwell Road London SW7 5BD UK; ^5^ School of Earth Sciences University of Bristol Life Sciences Building Tyndall Avenue Bristol BS8 1TQ UK

**Keywords:** angiosperms, Bayesian analysis, Cretaceous Terrestrial Revolution, divergence time, fossil record

## Abstract

- Through the lens of the fossil record, angiosperm diversification precipitated a Cretaceous Terrestrial Revolution (KTR) in which pollinators, herbivores and predators underwent explosive co‐diversification. Molecular dating studies imply that early angiosperm evolution is not documented in the fossil record. This mismatch remains controversial.
- We used a Bayesian molecular dating method to analyse a dataset of 83 genes from 644 taxa and 52 fossil calibrations to explore the effect of different interpretations of the fossil record, molecular clock models, data partitioning, among other factors, on angiosperm divergence time estimation.
- Controlling for different sources of uncertainty indicates that the timescale of angiosperm diversification is much less certain than previous molecular dating studies have suggested. Discord between molecular clock and purely fossil‐based interpretations of angiosperm diversification may be a consequence of false precision on both sides.
- We reject a post‐Jurassic origin of angiosperms, supporting the notion of a cryptic early history of angiosperms, but this history may be as much as 121 Myr, or as little as 23 Myr. These conclusions remain compatible with palaeobotanical evidence and a more general KTR in which major groups of angiosperms diverged later within the Cretaceous, alongside the diversification of pollinators, herbivores and their predators.

## Introduction

Angiosperms constitute one of the largest scions of the tree of life. They dominate extant plant diversity, occupy almost every habitat on Earth and are one of the principal components of modern biota playing crucial roles in terrestrial ecosystems (Augusto *et al*., 2014; Cascales‐Miñana *et al*., 2016). Angiosperms rose to ecological dominance in the Cretaceous Terrestrial Revolution (KTR), when their apparently explosive radiation is believed to have underpinned the diversification of lineages that are key components of contemporary terrestrial environments, such as birds, insects, mammals and seed‐free land plants, foreshadowing modern terrestrial biodiversity (Dilcher, 2000; Benton, 2010; Meredith *et al*., 2011; Cardinal & Danforth, 2013; Augusto *et al*., 2014; Cascales‐Miñana *et al*., 2016). However, these hypotheses of co‐diversification rest largely on the perceived coincidence in the radiation of angiosperms and the renewal of trophic networks in terrestrial ecosystems. This is evidenced, not least, by the fossil record of tricolpate pollen in the Barremian, slightly younger Aptian floral assemblages, followed by an explosive increase in diversity in the middle and late Cretaceous (Doyle, 2008; Clarke *et al*., 2011; Magallón *et al*., 2015; Herendeen *et al*., 2017). Some interpret this evidence literally to reflect an explosive radiation from a Cretaceous crown‐ancestor, with the earliest macrofossil record of unambiguous crown‐angiosperms (Friis *et al*., 2000; Sun *et al*., 2002) dating back only to the mid‐Early Cretaceous (Hickey & Doyle, 1977; Benton, 2010; Friis *et al*., 2010; Meredith *et al*., 2011; Doyle, 2012; Gomez *et al*., 2015; Cascales‐Miñana *et al*., 2016; Herendeen *et al*., 2017). In stark contrast, molecular timescales for angiosperm evolution have invariably concluded that crown‐angiosperms diverged as much as 100 million yr (Myr) earlier than the KTR (e.g. Bell *et al*., 2005, 2010; Magallón, 2010, 2014; Smith *et al*., 2010; Clarke *et al*., 2011; Magallón *et al*., 2013; Zanne *et al*., 2014; Zeng *et al*., 2014; Beaulieu *et al*., 2015; Foster *et al*., 2016; Murat *et al*., 2017) – unless they have been forced to fit with the early fossil record angiosperms (Magallón & Castillo, 2009; Magallón *et al*., 2015) – (Table 1), implying a long cryptic evolutionary history unrepresented in the fossil record. This may be because early angiosperms were not ecologically significant, or were living in environments in which fossilization was unlikely (Raven & Axelrod, 1974; Feild *et al*., 2009; Friedman, 2009; Smith *et al*., 2010; Doyle, 2012). Or it may be that molecular clock estimates are just unrealistically old, perhaps an artefact of their failure to accommodate dramatic accelerations that may have been associated with an explosive diversification of angiosperms (Magallón, 2010; Beaulieu *et al*., 2015; Brown & Smith, 2017).

**Table 1:** Overview of estimates of divergence times for selected major groups of angiosperms for some selected analyses from previous studies

Study	Data/analysis	Clade (crown group)							
		Angiosperms	Magnoliids	Monocots	Eudicots	Superrosids	Rosids	Superasterids	Asterids
Bell *et al*. (2005)	Loci: 2‐plastid, 1‐mt, 1‐nuc. Taxa: 71. Calib: 5./BRC	140–180 Ma	–	99–133 Ma	93–125 Ma	–	–	–	–
	Loci: 2‐plastid, 1‐mt, 1‐nuc. Taxa: 71. Calib: 5./PL	155–198 Ma	–	123–126 Ma	–	–	–	–	–
Magallón & Castillo (2009)	Loci: 3‐plastid. Taxa: 256. Calib: 13./PL	130–242 Ma	–	–	–	–	–	–	–
Bell *et al*. (2010)	Loci: 2‐plastid, 1‐nuc. Taxa: 567. Calib: 36a./IR	141–154 Ma	121–130 Ma	–	123–134 Ma	111–121 Ma	97–105 Ma	113–132 Ma	98–111 Ma
	Loci: 2‐plastid, 1‐nuc. Taxa: 567. Calib: 36b./IR	167–199 Ma	108–138 Ma	–	123–139 Ma	111–135 Ma	97–132 Ma	113–131 Ma	98–119 Ma
Smith *et al*. (2010)	Loci: 2‐plastid, 1‐nuc. Taxa: 154. Calib: 33./IR	182–257 Ma	136–181 Ma	139–167 Ma	128–147 Ma	–	–	–	–
	Loci: 2‐plastid, 1‐nuc. Taxa: 154. Calib: 32./IR	193–270 Ma	138–198 Ma	141–191 Ma	138–172 Ma	–	–	–	–
Clarke *et al*. (2011)	Loci: 7‐plastid. Taxa: 18. Calib: 17./IR	175–240 Ma	–	–	83–115 Ma	–	–	–	–
Magallón *et al*. (2013)	Loci: 5‐plastid. Taxa: 80. Calib: 28./IR	162–210 Ma	131–155 Ma	125–145 Ma	120–129 Ma	–	–	–	–
Magallón (2014)	Loci: 5‐plastid. Taxa: 81. Calib: 27./IR	162–210 Ma	–	–	–	–	–	–	–
Zanne *et al*. (2014)	Loci: 11‐plastid, 4‐mt, 2‐nuc. Taxa: 32 223. Calib: 39./PL	243 Ma	147 Ma	171 Ma	137 Ma	118 Ma	117 Ma	117 Ma	108 Ma
Zeng *et al*. (2014)	Loci: 59‐nuc. Taxa: 61. Calib: 2./IR	286–246 Ma	122–150 Ma	127–149 Ma	115–126 Ma	–	–	–	–
Magallón *et al*. (2015)	Loci: 3‐plastid, 2‐nuc. Taxa: 798. Calib: 137./IR	139.4 Ma	130–134 Ma	132–135 Ma	130–133 Ma	119–125 Ma	115–123 Ma	120–126 Ma	110–119 Ma
Beaulieu *et al*. (2015)	Loci: 3‐plastid, 1‐nuc. Taxa: 125. Calib: 24./IR	210–253 Ma	160–195 Ma	149–181 Ma	142–170 Ma	124–144 Ma	113–136 Ma	120–143 Ma	99–119 Ma
Foster *et al*. (2016)	Loci: 76‐plastid. Taxa: 195. Calib: 37./IR	192–251 Ma	130–171 Ma	141–176 Ma	136–154 Ma	123–135 Ma	118–131 Ma	107–126 Ma	108–124 Ma
Murat *et al*. (2017)	Loci: 1175. Taxa: 37. Calib: 2./IR	190–238 Ma	–	–	87–109 Ma	–	–	–	–
This study (composite)	Loci: 77‐plastid, 4‐mt, 2‐nuc. Taxa: 644. Calib: 52./IR	149–256 Ma	128–190 Ma	123–181 Ma	129–188 Ma	118–162 Ma	117–160 Ma	118–164 Ma	107–146 Ma

BRC, Bayesian relaxed clock (Multidivetime); PL, penalized likelihood; AR, autocorrelated rates model; IR, independent rates model; SC, strict clock model; Calib, calibration points; composite, 95% high posterior density credibility interval (HPD CI) is a composite of the 95% HPD credibility intervals across all calibration strategies, except calibration strategy B (SB). Ma, million years ago. See original works for further information on time estimates.

Moreover, the timescale of angiosperm diversification varies broadly amongst different molecular analyses (Table 1). This is not surprising given that the transformation of molecular distances (the branch lengths on a phylogeny) into geological divergence times is challenging (dos Reis & Yang, 2013). Certainly, there are a number of methodological variables in previous molecular analyses which are known to affect the accuracy and precision of divergence time estimates (dos Reis *et al*., 2016). Foremost among these is the approach taken in establishing fossil calibrations, which have been shown to contribute the greatest source of uncertainty associated with molecular clock analyses (Sauquet *et al*., 2012; Magallón *et al*., 2013; dos Reis & Yang, 2013; Warnock *et al*., 2015, 2017). Hence, a suite of best practices has been established for the formulation of fossil calibrations (Parham *et al*., 2012), but these have not generally been applied to angiosperms. Foster *et al*. (2016) have highlighted the particular challenge of dating angiosperm divergence accurately using the low taxon sampling common to theirs and other studies (e.g. Bell *et al*., 2005, 2010; Magallón, 2010, 2014; Smith *et al*., 2010; Clarke *et al*., 2011; Magallón *et al*., 2013; Zeng *et al*., 2014; Beaulieu *et al*., 2015; Foster *et al*., 2016; Murat *et al*., 2017). Some previous analyses are also limited by either insufficient outgroup lineages (e.g. Bell *et al*., 2005, 2010; Zeng *et al*., 2014; Magallón *et al*., 2015; Foster *et al*., 2016), very limited sequence data (e.g. Bell *et al*., 2005, 2010; Magallón & Castillo, 2009; Magallón, 2010, 2014; Smith *et al*., 2010; Clarke *et al*., 2011; Magallón *et al*., 2013, 2015; Beaulieu *et al*., 2015), and usually a combination thereof. Finally, simulations have shown that the convention of interpreting the results of Bayesian divergence time analyses in terms of the mean or median of a broad posterior probability distribution, when the credibility intervals (CIs) are wide, results in false precision (Warnock *et al*., 2017).

In an attempt to explore the impact of these variables on the mismatch between molecular clock estimates and fossil evidence for the origin and diversification of angiosperms, we compiled a molecular dataset of nucleotide and amino acid sequences from 83 plastid, mitochondrial and nuclear genes from 644 taxa (Soltis *et al*., 2011; Ruhfel *et al*., 2014). This encompasses the diversity of angiosperms as well as seed plant, fern and lycophyte outgroups, simultaneously addressing concerns of taxon and locus diversity, as well as outgroup inclusion. We used these data both to estimate tracheophyte interrelationships by maximum likelihood (ML) and the timescale over which this phylogeny unfolded; the large scale of the dataset is important not only for testing established phylogenetic hypotheses, but also improving timescale precision (dos Reis *et al*., 2012, 2016; dos Reis & Yang, 2013). Given the prevalence of rate variation, a rich suite of calibrations serves to provide local checks on the substitution rate across tracheophyte phylogeny (Hugall *et al*., 2007). We employed 52 fossil calibrations, all of which achieve the expectations of established best practice (Parham *et al*., 2012). We combined the molecular data and fossil calibrations in a Bayesian relaxed clock divergence time analysis. The Bayesian approach used here (Rannala & Yang, 2007; dos Reis & Yang, 2011) integrates over the uncertainty in rate variation along the phylogeny. We explored the impact of different sources of uncertainty on the timescale of angiosperm diversification. We employed five calibration strategies that accommodate different interpretations of the fossil record, and showed that these have a strong impact on posterior estimates. We also explored the impact of data partitioning, parameter choice in priors for rates and times, relaxed molecular clocks and the effect of outgroup sampling.

Above all, our aim was to establish a holistic evolutionary timescale for angiosperms, based on a broad exploration of analytic parameter space that encompasses all major sources of uncertainty. This provides the best opportunity of ameliorating the disparity between contemporary molecular clock estimates, which predict a deep Jurassic or Triassic origin of crown‐angiosperms, and interpretations of the palaeobotanical record that advocate an explosive Early Cretaceous radiation (Herendeen *et al*., 2017).

## Materials and Methods

### Molecular data assembly

We assembled a dataset comprising 83 genes from 644 taxa (632 angiosperms, eight gymnosperms, two ferns and two lycophytes) from three sources. First, sequences for 16 genes (10 plastid, four mitochondrial, two nuclear) from 640 taxa were retrieved from GenBank using the accession numbers from Soltis *et al*. (2011). As many gene sequences in the alignment of Soltis *et al*. (2011) were partial sequences or a mixture of coding and non‐coding segments (introns or spacers), we cleaned and curated their list of GenBank accession numbers and retrieved the sequences again. CDS sequences for each coding gene, as well as partial or complete sequences for nuclear rRNA genes, were retrieved. Each gene was realigned using the Mafft algorithm (Katoh & Standley, 2013) implemented in TranslatorX (Abascal *et al*., 2010) and curated. This process did not recover the original alignments of Soltis *et al*. (2011) and extra species and gene sequences previously missing or incomplete were added to the dataset. Second, sequences for 78 plastid genes from 110 taxa were taken from Ruhfel *et al*. (2014). Eleven genes in the dataset were also found to be in the dataset of Soltis *et al*. (2011), and were removed. Third, sequences for an additional 16 genes from two ferns and two lycophytes were obtained from GenBank, aligned using Mafft. Gene alignments from all three sources were combined into one dataset using SeaView (Gouy *et al*., 2010).

For each gene, a phylogenetic tree was constructed by ML using RAxML 7.7.8 (Stamatakis *et al*., 2005) (Supporting Information Table S1). Sequences with unusually long external branches (that accounted for > 30% of the total tree length) were removed (*nad5* for *Selaginella* and *rps4* for *Huperzia*). GenBank accession numbers for all sequences are available on Figshare. The final alignment includes 83 genes and has 75 030 base pairs (bp) with 71.4% missing data. This was divided into five partitions: 1^st^ and 2^nd^ codon positions for plastid genes; 3^rd^ positions for plastid genes; 1^st^ and 2^nd^ codon positions for mitochondrial genes; 3^rd^ positions for mitochondrial genes; and nuclear RNA genes. The large amount of missing data did not seem to be an impediment to this combined approach (Roure *et al*., 2013; Zheng & Wiens, 2016); the broad phylogenetic relationships were very similar to those from the analysis of 81 taxa (36% missing data) or 48 taxa (26% missing data). Some basic information about the five partitions obtained using RAxML, such as the tree length and tree topology, is given in Table S2 and Figs S1–S3. The molecular sequence alignment and the GenBank accession numbers have been deposited in Figshare: https://figshare.com/s/404b70bc39656c2cf57e.

### Tree topology

**Figure 1:**
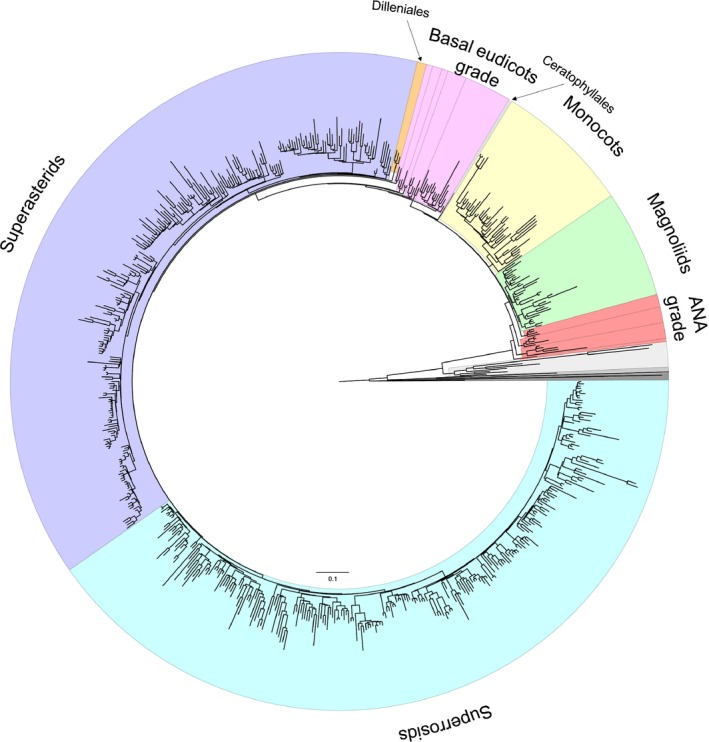
RAxML tree estimated from the 83 genes and 644 taxa of tracheophytes. The major angiosperm lineages and grades are highlighted: ANA grade (red), magnoliids (green), monocots (yellow), Ceratophyllales (pale blue), basal eudicots grade (pink), Dilleniales (orange), superasterids (purple) and superrosids (blue). Species names and bootstrap support values are indicated in Supporting Information Fig. S3.

The final alignment, with the five partitions as described above, was used to estimate the ML tree using RAxML, under the GTR + Γ model with 100 bootstrap replicates. The model assumes independent substitution parameters, with joint branch length optimization. The ML tree (Figs 1, S4) was used for subsequent molecular clock dating analyses.

### Fossil calibrations

**Figure 2:**
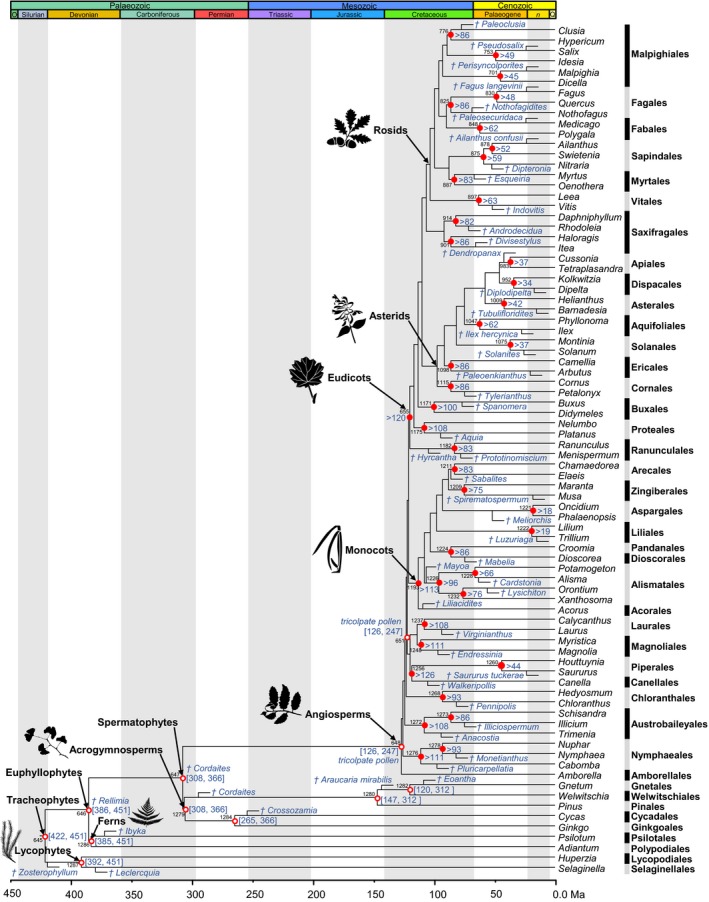
Summary tree of tracheophytes showing fossil calibrations. Calibrations are represented for 52 nodes, consisting of (>) soft minimum (closed red dots) or both ([min, max]) soft minimum and soft maximum (open red dots). Calibrated nodes are numbered as in Supporting Information Fig. S2. Justifications for these minima and maxima are provided in Notes S1 and an overview in Table S3. The dagger symbol shows a species that is extinct. The tree has been scaled to time on the basis of the minimum constraints.

Bayesian clock dating was conducted using the McmcTree program from the Paml4.8 package (Yang, 2007) incorporating soft‐bound fossil calibrations on nodes on the tree (Yang & Rannala, 2006). The calibrations (Fig. 2; Table S3; Notes S1) were formulated on the basis of: a specific fossil specimen reposited in a publically accessible collection; an apomorphy‐based justification of clade assignment; reconciliation of morphological and molecular phylogenetic context of clade assignment; geographic and stratigraphic provenance; justification of geochronological age interpretation (Parham *et al*., 2012). The inclusion of hierarchically nested outgroups allows us to take advantage of the effects of truncation in the construction of the joint time prior, which serves to preclude phylogenetically incompatible clade ages (i.e. ancestral nodes younger than descendants) from being proposed simultaneously to the Mcmc (Inoue *et al*., 2010). In this way, the conservative maximum constraint on the age of the angiosperm total group is diminished because of temporal overlap with the specified time prior on the spermatophyte, euphyllophyte and tracheophyte clades.

We employed five calibration strategies to accommodate different interpretations of the fossil record. In all, we used the independent rates (IR) model to specify the prior of evolutionary rates on branches on the tree topology. The 83‐gene dataset was subdivided and analysed as three partitions (3P) under the HKY85 + Γ5 substitution model, with third codon positions excluded from all analyses. In the first calibration strategy (SA), the 11 calibrations for which soft maximum constraints were available (Fig. 2; Table S3) were modelled using a prior probability of 94% for a uniform distribution bounded by the minimum and maximum fossil constraints B(*t*_L_, *t*_U_, *p*_L_, *p*_U_), a 1% power decay distribution on the minimum constraint (*p*_L_ = 0.01) and a 5% exponential decay on the maximum constraint (*p*_U_ = 0.05). The remaining 41 calibration nodes have minimum bounds only (Fig. 2; Table S3), specified using a truncated Cauchy distribution *L*(*t*_L_, *p*,* c*,* p*_L_), where *p* determines how far from the bound is the mode of the distribution, *c* determines how sharply the distribution decays to zero and *p*_L_ is the left tail probability (Inoue *et al*., 2010). We used *p *=* *0.1, *c *=* *0.1 and *p*_L_ = 0.01; this reflects a prior belief that the fossil minima are a close approximation of clade age. It is our view that this calibration strategy best reflects the available palaeobotanical and phylogenetic evidence, while also controlling for analytic variables, particularly the impact of construction of the joint time prior on specified calibrations (Warnock *et al*., 2017). However, we also explored the impact of: relaxing these calibrations in calibration strategy SB; further skewing the probability of the age of the angiosperm crown‐ancestor to approximate the fossil minimum in calibration strategies SC and SD; and forcing the age of the angiosperm crown‐ancestor to approximate the fossil minimum in calibration strategy SE.

In the second calibration strategy (SB), the 41 node calibrations with minimum bound inherit the maximum bound from the youngest ancestor which has a maximum bound, so that each of the 52 calibrations has a pair of minimum and maximum bounds. The prior probability of clade age was established by a uniform distribution between minimum and maximum bounds reflecting agnosticism about the true time of divergence between these bounds. Again, we used *p*_L_ = 0.01 and *p*_U_ = 0.05. The remaining three calibration strategies C to E (SC–SE) follow the first (SA), but implement different calibration densities for the crown of angiosperms (node 648 in the tree of Fig. S5) and mesangiosperms (node 451 of Fig. S5). Calibration strategies SC and SD used the truncated Cauchy distribution with either a medium tail (*c *=* *0.01) (SC) or a short tail (SD) (*c *=* *0.005) extending back in time, reflecting a view that the fossil minimum constraints are increasingly closer approximations of clade age as the bulk of the probability density is skewed towards the minimum constraint as the value of *c* diminishes. For completeness, to explore the impact of accepting the conventional palaeobotanical interpretation of a Cretaceous origin of crown‐angiosperms (e.g. Herendeen *et al*., 2017), analysis SE used an optimistic maximum (139.4 million yr ago (Ma)) soft bound for crown‐angiosperms and crown‐mesangiosperms based on an estimate of Magallón *et al*. (2015). The time unit was set to 100 million years (Myr) (phylogenetic trees in Newick format with fossil calibrations available on Figshare: https://figshare.com/s/404b70bc39656c2cf57e).

### Bayesian divergence time estimation

To examine the robustness of the posterior time estimates, several analyses were performed by changing prior assumptions and parameter settings. These include data partitioning, calibration strategies, parameter choice for priors for rates and times, birth–death process parameters and exclusion of distantly related outgroups with very long branches.

Our dating analyses used three of the five partitions described earlier, with the two partitions for third codon positions (in plastid and mitochondrial genes) excluded. The alignment had 51 792 bp, with 70.5% missing data. Our ‘standard’ analysis (SA‐IR‐3P) uses calibration strategy A, IR model (Thorne *et al*., 1998; dos Reis & Yang, 2011) and HKY85 + Γ5 substitution model (Yang & Rannala, 2006), with three partitions. The three partitions were 1^st^ and 2^nd^ codon positions for plastid genes, 1^st^ and 2^nd^ codon positions for mitochondrial genes, and nuclear RNA genes, as described above. In the IR model, the rate for any branch is a random variable from a lognormal density LN(μ*,* σ^2^), where μ is the mean of the rate and σ^2^ is the variance of the log rate. A gamma prior G(2, 50) was specified for μ, with a mean of 0.04 substitutions per site per 100 Myr or 4 × 10^−10^ substitutions per site yr^−1^. This is based on rough estimates of substitution rates obtained by fitting a strict molecular clock to the sequence data, using a point calibration (vascular plants, 438 Ma) on the root. A gamma prior G(2, 4) was assigned for σ^*2*,^ with mean of 0.5. The prior on times was constructed using fossil calibration densities combined with the birth–death sampling process, which specifies the distribution of the ages of non‐calibrated nodes (Yang & Rannala, 2006). The parameter values λ *= *μ *=* 1 and *p *=* *0 specified a uniform kernel.

We conducted ten additional analyses that are variations of the standard analysis to examine the robustness of the posterior time estimates. We examined the truncation effect among the calibrated nodes by generating the joint prior of times by running the Mcmc without data. We used the four alternative calibration strategies to assess the impact of the calibration strategy, resulting in Analyses SB‐IR‐3P, SC‐IR‐3P, SD‐IR‐3P and SE‐IR‐3P. To assess the effect of the number of partitions, we set up two analyses. In Analysis SA‐IR‐1P, the three partitions were concatenated and treated as a single partition, and, in Analysis SA‐IR‐MP, a mixed alignment, divided into plastid proteins, mitochondrial proteins and nuclear RNA genes, was used. To assess the impact of the birth–death sampling prior, the parameters of the birth–death model were altered such that the kernel had an L shape (λ = 1, μ = 4 and ρ = 0.1), giving a tree with long internal branches (Analysis SA‐IR‐3P‐BD1), or an inverted L shape (λ = 4, μ = 1 and ρ = 0.0001), giving a tree with long terminal branches (Analysis SA‐IR‐3P‐BD2). To assess the effect of the rate model, Analysis SA‐AR‐3P was conducted under the autocorrelated rates (AR) model (Rannala & Yang, 2007). Finally, to explore the effect of excluding distantly related outgroups, lycophytes and ferns were removed from the alignment (Analysis SA‐IR‐3P‐EP). In this analysis, we used a gamma prior G(2, 60) for μ with a mean of 0.03 substitutions per site per 100 Myr or 3 × 10^−10^ substitutions per site yr^−1^, based on a rough substitution rate estimate obtained by fitting a strict molecular clock to the sequence data, using a point calibration (seed plants, 337 Ma) on the root.

To evaluate the performance of different relaxed clock models, we used marginal likelihood calculation to estimate Bayes factors and posterior model probabilities. The marginal likelihood is hard to calculate, but, recently, methods such as path‐sampling (thermodynamic integration) and stepping‐stones have been integrated within phylogenetics (Lartillot & Philippe, 2006; Lepage *et al*., 2007; Linder *et al*., 2011; Xie *et al*., 2011; Baele *et al*., 2012). Here, we used the thermodynamic integration with Gaussian quadrature method (Rannala & Yang, 2017), which has been recently implemented in McmcTree (dos Reis *et al*., 2017), to calculate the marginal likelihoods for the strict clock (SC), IR and AR models. Because thermodynamic integration is computationally expensive (it must use exact likelihood calculations), we estimated the marginal likelihood for the three clock models using a smaller dataset of ten tracheophyte species (*Huperzia*,* Psilotum*,* Ginkgo*,* Amborella*,* Nymphaea*,* Acorus*,* Calycanthus*,* Platanus*,* Oxalis* and *Cornus*) for the four partitions analysed (Table S2).

The likelihood (or the probability of the sequence alignment given the tree and branch lengths) was calculated using the approximate method (Thorne *et al*., 1998; dos Reis & Yang, 2011), employing the SQRT transformation (dos Reis & Yang, 2011). ML estimates of branch lengths and the Hessian matrix were calculated using the programs Baseml and Codeml. We used the HKY85 + Γ5 model for nucleotide alignments, the cpREV64 substitution model for plastid proteins and the WAG model for the mitochondrial proteins. For each analysis, the Mcmc was run for *c*. 5.5 million iterations after a ‘burnin’ of 250 000 iterations. The chain was sampled every 80 iterations until *c*. 70 000 samples were collected. Each analysis was performed at least twice, and consistency between runs was used as a major check on Mcmc convergence. We also compared the posterior mean times and plotted the time series traces using the Mcmc samples. The resulting posterior distribution was summarized as the posterior means and 95% equal‐tail CIs for divergence times.

## Results

### Topology estimation and the effect of fossil calibration uncertainty

**Figure 3:**
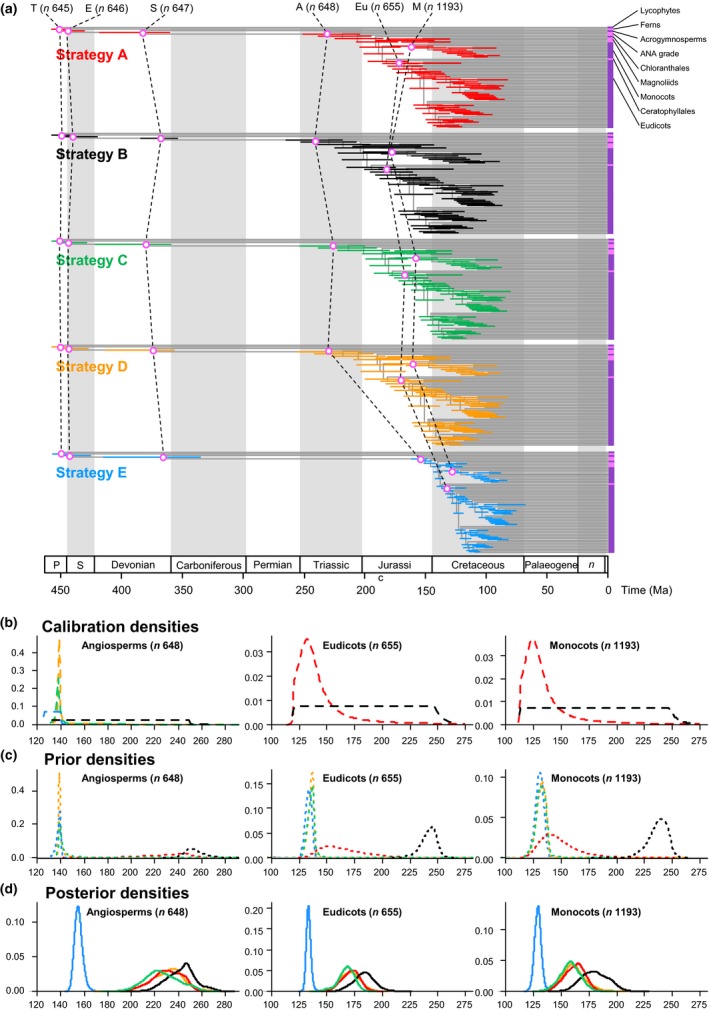
The effect of calibrations on posterior divergence time estimates of major groups of tracheophytes and angiosperms. (a) Summary chronogram for tracheophytes (including two lycophytes, two ferns, eight gymnosperms and 64 orders of angiosperms) with terminals collapsed to represent angiosperm orders showing divergence time estimates. Nodes are drawn at the posterior means obtained and horizontal bars represent 95% high posterior density (HPD) credibility intervals (CIs). Estimates were obtained using the HKY85 + Γ5 substitution model, independent rates model (IR), with the 83 genes subdivided into three partitions: 1^st^ and 2^nd^ codon positions for plastid genes; 1^st^ and 2^nd^ codon positions for mitochondrial genes; and nuclear RNA genes. Five nodes are connected (purple open dots) across the analyses to facilitate comparison: tracheophytes (*n *=* *645), seed plants (*n *=* *647), angiosperms (*n *=* *648), eudicots (*n *=* *655) and monocots (*n *=* *1193). (b–d) Calibration, prior and posterior densities for three angiosperm nodes in the tracheophyte phylogeny. Colouring relates to the calibration strategy as in (a). The phylogeny with clade names is provided in Fig. 6. Nodes in parentheses are numbered as in Supporting Information Fig. S2.

**Figure 4:**
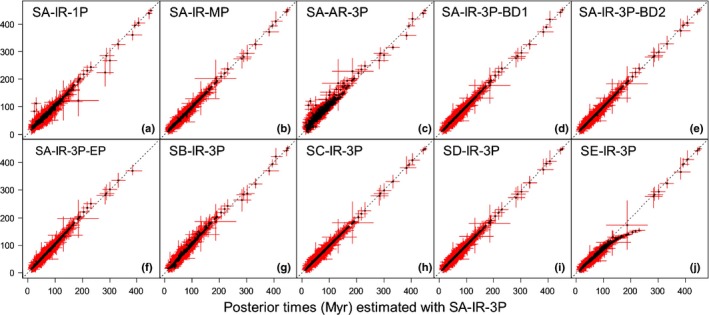
Sensitivity of time estimates to the number of partitions, rate model, birth–death process, exclusion of lycophytes + ferns and fossil calibrations. The posterior mean times (black dots) and 95% credibility intervals (CIs) (red lines) of 643 nodes under calibration strategy A (SA), independent rates (IR) model, and gene alignments and three partitions are plotted against (a) estimates using one partition, (b) mixed partitions, (c) autocorrelated rates (AR) model, (d) birth–death parameters adjusted to generate a tree with long internal branches and short tip branches (BD1), and (e) large node ages with nodes close to the root (BD2), (f) excluding ferns and lycophytes (EP), (g) calibration strategy B (SB), (h) calibration strategy C (SC), (i) calibration strategy D (SD) and (j) calibration strategy E (SE).

**Figure 5:**
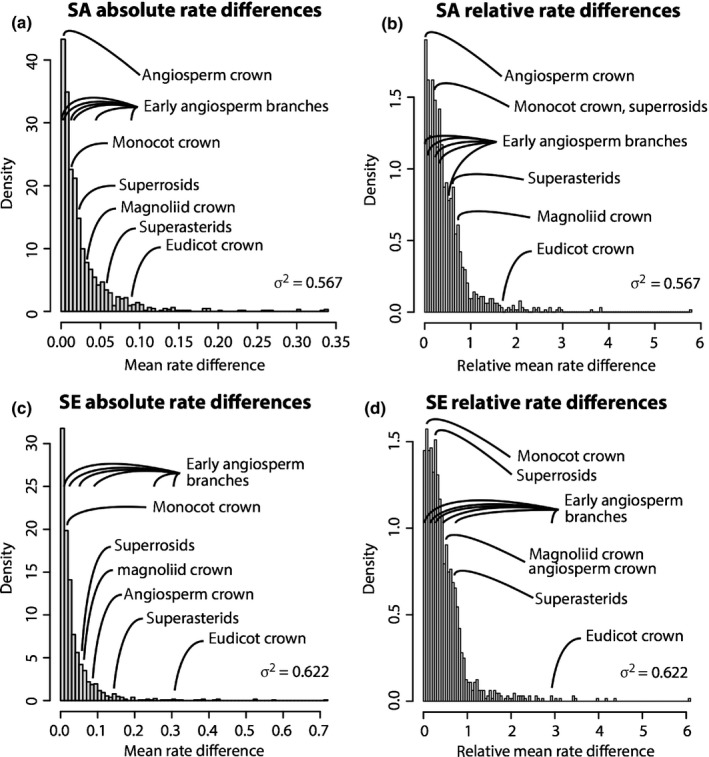
Branch rate differences inferred from competing calibration strategies. All rate differences are plotted as positive regardless of whether they represent rate accelerations or decelerations. (a) Absolute and (b) proportional rate differences based on calibration strategy A (SA) that does not force an Early Cretaceous diversification of crown‐angiosperms (but remains compatible with this scenario). (c) Absolute and (d) proportional rate differences based on calibration strategy E (SE), which forces an Early Cretaceous diversification of crown‐angiosperms. Key early angiosperm nodes are labelled. Note that the eudicot crown is an outlier in all, but early angiosperm clade rates fall within the bounds exhibited by other, younger nodes in the tree, indicating that the independent rates (IR) model can accommodate heterogeneous rates required by an Early Cretaceous diversification of crown‐angiosperms.

We recovered a topology in which deep‐level relationships among angiosperms are resolved with confidence and most branches are supported with a bootstrap value of 100% (Figs 1, S4). To explore the robustness of angiosperm divergence time estimates to calibration choice, we employed five calibration strategies that shared the same palaeontological constraints (Fig. 2; Table S3; Notes S1), but differed in their interpretation of this evidence, expressed as different statistical distributions (Fig. S6). The results of these analyses demonstrated that the calibration strategy has a strong impact on the estimated divergence times (Figs 3a, 4g–j, S6; Tables 1, S4). Estimates based on SA indicate that crown‐angiosperms originated at 255–206 Ma, crown‐eudicots at 186–156 Ma and crown‐monocots at 179–144 Ma (Figs S5, S6; Tables 1, S4). Using shorter tail calibration densities on the key nodes of crown‐angiosperms and crown‐mesangioperms (SC, SD) had no significant impact on the resulting posterior time estimates (Figs 3d, 4h,i, S6; Tables 1, S4). By contrast, calibration strategy SB produced older estimates and larger intervals than all the other calibration strategies (crown‐angiosperms at 266–219 Ma, crown‐eudicots at 201–164 Ma and crown‐monocots at 203–127 Ma; Figs 3d, S6; Tables 1, S4). This occurs because this calibration strategy is uninformative on the timing of divergence between minimum and maximum constraints, and the effect of truncation in the construction of the joint time prior results in effective priors on node ages that place the majority of the probability mass near the maximum age bound (Figs 3d, S6). In effect, the fossil minima are considered to be a poor approximation of clade age. This is particularly apparent in the marginal priors (and posteriors) for crown clades of angiosperms, mesangiosperms, monocots, eudicots (Figs 3c,d, S6), Alismatales, Laurales and stem‐Canellales. Calibration strategy SE considered whether molecular estimates could be forced into agreement with fossil evidence, employing an unrealistically optimistic 139.4 Ma maximum constraint on the age of crown‐angiosperms. Unsurprisingly, this yielded significantly younger and more precise time estimates for crown clades of angiosperms (162–149 Ma), eudicots (137–129 Ma) and monocots (135–123 Ma), together with many other clades (Figs 3, 4j, S6; Tables 1, S4). Nonetheless, the inferred age of crown‐angiosperms remains significantly older than the earliest unequivocal fossil evidence (125.9 Ma). Furthermore, the rate differences across early crown‐angiosperm nodes do not differ significantly between calibration strategies SA and SE (Fig. 5).

### Impact of partition strategy on divergence time estimates

Divergence time estimation can also be affected by the manner in which the molecular sequence alignment is partitioned (Zhu *et al*., 2015). Thus, we considered three different partition schemes. In the first (3P), the sequence alignment was subdivided into three partitions (excluding 3^rd^ codon positions): 1^st^ and 2^nd^ codon positions for plastid genes; 1^st^ and 2^nd^ codon positions for mitochondrial genes; and nuclear RNA genes. In the second (1P), these partitions were concatenated and analysed as a single partition. Our third partition strategy (MP) was a mixed alignment divided into plastid proteins, mitochondrial proteins and nuclear RNA genes. Divergence time analysis using partition scheme 1P yielded the least precise estimates (Table S5) and the posterior mean age estimates are the least compatible with the other partition schemes (Fig. 4a; Table S5). Estimates using 3P and MP are more precise and much more consistent with one another (Fig. 4b; Table S5), although the improvement is more marked between one partition and three partitions, than between three nucleotide partitions and three hybrid partitions, suggesting that 3P achieves the best trade‐off between increasing analytical complexity and accuracy.

### Impact of rate model on divergence time estimates

Rate models can also affect divergence time estimation when the molecular clock is seriously violated (dos Reis *et al*., 2015), as it is amongst angiosperms (Beaulieu *et al*., 2015). When the clock is violated, rates calculated in one part of the phylogeny serve as a poor proxy for estimating divergence times in other clades. To assess the effect of this uncertainty, we estimated divergence times for tracheophytes assuming an AR model under calibration strategy SA. In attempting to encompass the uncertainty in the rate drift model, we considered here the spread of node age estimates that arises from both rate models (Fig. 4c). Our results show that the AR model produces older estimates for shallow nodes and younger estimates for deep nodes, in comparison with the IR model, where a few nodes, especially the deep nodes, are younger (Fig. 4c; Table S5). Moreover, we tested a series of informative priors on the overall rate based on the rough rate estimates mentioned above. However, these priors did not affect time estimates noticeably, possibly because a large number of fossil calibrations constrain the time prior.

### Bayes factor calculation for clock model selection

The results of Bayesian selection of the clock model are presented in Table 2. The IR model always had the highest marginal likelihood, with the posterior model probability > 90% in all datasets. Therefore, we conclude that, overall, the IR model is the most appropriate model of rate variation on the tracheophyte data analysed here, and the divergence times calculated under the IR model should be preferred. We would expect these results to apply to the larger datasets used in the estimation of divergence times, but further work is needed to confirm this.

**Table 2:** Bayesian model selection of rate model

Dataset	Clock model	Log marginal *L*	BF	*P*
Plastid 1^st^ and 2^nd^ c.p.	SC	–141 585.67	5.1 × 10^−274^	5.05 × 10^−274^
	**IR**	**–140 956.40**	–	**0.991**
	AR	–140 961.16	0.009	0.009
Mitochondrial 1^st^ and 2^nd^ c.p.	SC	–13 776.34	7.86 × 10^−29^	7.79 × 10^−29^
	**IR**	**–13 711.64**	–	**0.991**
	AR	–13 716.36	0.009	0.009
Nuclear RNA	SC	–17 534.24	2.15 × 10^−41^	2.03 × 10^−41^
	**IR**	**–17 440.60**	–	**0.944**
	AR	–17 443.43	0.059	0.056
Concatenation (pl1&2, mt1&2, nucRNA)	SC	–173 121.00	1.03 × 10^−297^	1.02 × 10^−297^
	**IR**	**–172 437.16**	–	**0.988**
	AR	–172 441.60	0.012	0.012

SC, strict clock model; IR, independent rates model; AR, auto‐correlated rates model. The age of the root is fixed to one (i.e. we used a ‘B(0.99, 1.01)’ calibration on the root in McmcTree). The rate prior used is G(2, 10). The prior on σ^2^ is G(2, 4) in all cases. The model with the highest posterior probability in each dataset is shown in bold type.

### Impact of diversification model on divergence time estimates

We also explored the impact of the birth–death process used to specify the prior of times on the divergence time estimation. The parameters of the birth–death process with species sampling were fixed at λ = 1, μ = 1, ρ = 0, which generates uniform node ages. We assessed uncertainty by adjusting parameters λ, μ and ρ such that the kernel had an L shape (λ = 1, μ = 4, ρ = 0.1), giving a tree with long internal branches (BD1), or an inverted L shape (λ = 4, μ = 1, ρ = 0.0001), giving a tree with long terminal branches (BD2). The results of these two parameter sets are almost identical to those from the original setting (Fig 4d,e; Table S5), suggesting that parameter selection for the birth–death does not have a significant impact on divergence time estimates for this dataset.

### Impact of outgroup sampling on divergence time estimates

Finally, we considered the impact of the choice of outgroups on divergence time estimation. We included several outgroups to seed plants so that we could consider the timing of angiosperm origin in the context of land plant diversification as a whole. However, ferns and lycophytes are distantly related clades comprised of long branches, and may therefore have biased our estimates. We explored the effect of including distantly related outgroups (tracheophyte dataset) and of excluding lycophytes and ferns (EP dataset). The results (Fig. 4f; Table S5) show that the inclusion of lycophytes and ferns did not have a strong effect on the posterior time estimates, although their exclusion did result in increased ages for some intermediate clades.

## Discussion

**Figure 6:**
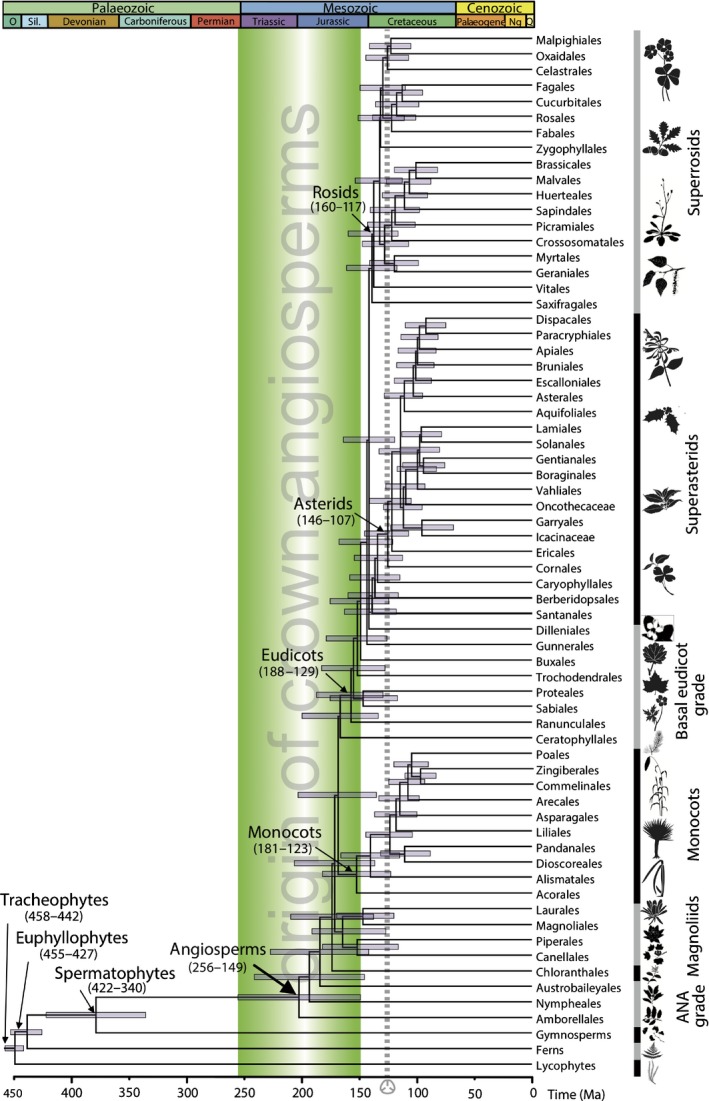
The time tree of tracheophytes encompassing uncertainty of calibration strategies. Holistic timescale for tracheophytes with terminals collapsed to represent angiosperm orders. Node ages are plotted at the posterior mean for calibration strategy A (SA), three partitions (3P), independent rates model (IR) and HKY85 + Γ5 substitution model. The node bars are composites extending from the minimum 2.5% high posterior density (HPD) limit to the maximum 97.5% limit across all calibration strategy analyses (excluding results from calibration strategy B). This timescale should be read in terms of the span of clade age uncertainty, not from the absolute position of the nodes, which are placed at an arbitrary midpoint. The interval of residual uncertainty associated with the angiosperm crown is highlighted.

Overall, the estimated divergence times for angiosperm clades are robust to variation in models and parameters, including the birth–death prior and the prior for rate parameters under the rate drift model. The main factors affecting the estimates are data partitioning, fossil calibration uncertainty, the discrepancy between the user‐specified time prior and the effective time prior, and the rate drift model. None of our component analyses provides an accurate timescale for angiosperm evolution as each one controls for a different source of uncertainty. Rather, it is necessary to integrate these uncertainties into a single timescale (Fig. 6; Table S4). This allows us to conclude that crown‐tracheophytes and crown‐euphyllophytes originated in the Late Ordovician–early Silurian interval (458–442 Ma and 455–427 Ma, respectively) and the crown‐spermatophytes within the latest Silurian–early Carboniferous (422–340 Ma). Crown‐angiosperms originated within the late Permian–latest Jurassic interval (256–149 Ma), whereas the crown clades of magnoliids, monocots and eudicots diverged between the Early Jurassic and Early Cretaceous (190–128 Ma, 181–123 Ma and 188–129 Ma, respectively), and the two main lineages of eudicots, the asterids and rosids, originated between the latest Jurassic and middle Cretaceous (146–107 Ma and 160–117 Ma, respectively). Whereas the age estimates for non‐angiosperm clades are close to their first fossil records, the conflicts between the molecular estimates of clade age and the fossil first occurrences are greater within angiosperms.

Recent studies have provided a great spread of molecular clock estimates for the origin of crown‐angiosperms (e.g. Bell *et al*., 2005, 2010; Magallón & Castillo, 2009; Magallón, 2010, 2014; Smith *et al*., 2010; Clarke *et al*., 2011; Magallón *et al*., 2013; Zanne *et al*., 2014; Zeng *et al*., 2014; Beaulieu *et al*., 2015; Foster *et al*., 2016; Murat *et al*., 2017) to the Lower Cretaceous (Bell *et al*., 2005, 2010; Magallón & Castillo, 2009; Magallón *et al*., 2015), covering the range 270–122 Ma. Our integrated timescale, which encompasses all of the unconstrainable sources of uncertainty we addressed (Fig. 6; Table S4), estimates crown‐angiosperms to have diverged in the interval 256–149 Ma, fully within the range of previous estimates (Table 1). Apart from a range of methodological differences, two factors account for many differences between our estimates and those obtained in previous studies. First, our interpretation of the analytic results in terms of the span of the posterior clade age estimate, in place of the convention of a precise, but inaccurate, point summary (Warnock *et al*., 2017). Second, the manner in which the palaeontological data are interpreted to implement fossil constraints; for example, analyses that yield Cretaceous estimates for the origin of angiosperms have used a Cretaceous point calibration or a concentrated calibration density, under the assumption that the age of crown‐angiosperms is known almost without error (Magallón & Castillo, 2009; Magallón *et al*., 2015). In general, recent molecular clock studies obtained estimates suggesting a Triassic origin of angiosperms. Hence, these molecular estimates raise the possibility that the oldest crown‐angiosperm fossils are still undiscovered, or at least unidentified.

The results of our experiments are compatible with this ‘long fuse’ interpretation, but they do not reject the ‘short fuse’ alternative. The discordance between molecular clock estimates and unequivocal fossil evidence of crown‐angiosperms implies a cryptic interval to their early evolutionary history, in which angiosperms existed but are unrepresented in the fossil record, which could be as much as 121 Myr, but as little as 23 Myr. However, the apparent mismatch may be more perceived than real. Although the early fossil record of angiosperms has been interpreted to reflect an orderly and incrementally phased environmental invasion (Hickey & Doyle, 1977; Coiffard *et al*., 2012; Doyle, 2012), this pattern may be an artefact imposed by the non‐uniformity of the rock record on the fossil record of all terrestrial clades (cf Benson *et al*., 2013). Furthermore, although the earliest unequivocal evidence of angiosperms, based on (Fischer's rule) tricolpate pollen, can be constrained minimally to the Barremian, this actually evidences the establishment of the eudicot lineage, which is remote from the angiosperm crown ancestor (Doyle & Hotton, 1991; Clarke *et al*., 2011). Monosulcate pollen, such as that produced by early‐branching lineages of extant angiosperms, is known at least as far back as the Valanginian (Brenner, 1996), and pollen exhibiting subsets of definitive crown‐angiosperm characters is known as far back as the Middle Triassic (Cornet, 1986; Doyle & Hotton, 1991; Taylor & Taylor, 2009; Hochuli *et al*., 2013), but these are difficult to discriminate from pollen produced by stem‐angiosperms or gymnosperms (Doyle & Hotton, 1991), and hence they have not been used to constrain divergence time analyses. There are also claims of pre‐Cretaceous crown‐angiosperms based on macrofossil evidence. Although the age of the angiosperm macrofossil genus *Archaefructus* (Sun *et al*., 2002; Friis *et al*., 2003) has been revised from Jurassic to Cretaceous (Chang *et al*., 2009), other putative pre‐Cretaceous angiosperm fossils are more securely dated, but their interpretation requires further attention (Crane *et al*., 1995; Taylor & Taylor, 2009; Friis *et al*., 2011; Doyle, 2012; Liu & Wang, 2016, 2017; Herendeen *et al*., 2017).

Discrimination between long and short fuse models of angiosperm diversification is problematic. It has been argued that predictions of a long cryptic early history for crown‐angiosperms is an artefact of the failure of molecular clock models that cannot accommodate the dramatic rate increases that some invoke to explain dramatic Early Cretaceous radiation (Beaulieu *et al*., 2015). The results of our experiments to discriminate among competing clock models indicate that the IR model provides a better fit than the AR relaxed clock model of the tracheophyte data. In the IR model, the variance of the rate is independent of time, and thus rate can undergo large shifts (depending on the value of σ^2^), even on adjacent branches. Under the AR model, variance depends on time, and hence the model penalizes large rate variation over short time intervals, but allows rate to vary approximately freely amongst distant clades. However, the variance increases linearly with time and, in analyses of deep phylogenies, this might lead to excessively high rate shifts. Therefore, the AR model might be more suitable for the analysis of closely related species and the IR model for the analysis of divergent species and large phylogenies. However, further research is needed to understand which clock model is the most biologically realistic and appropriate for real data analysis (Lepage *et al*., 2007; Ho, 2009; Linder *et al*., 2011). Nevertheless, our analyses of the rates implied by calibrations that force divergence time estimates into agreement with fossil clade age minima (Fig. 5) do not require rate differences across early crown‐angiosperm nodes that differ significantly from more recent angiosperm clades in the same analysis, or rate differences across the same nodes in analyses that do not force a close fit to fossil clade age minima (calibration strategies SA vs SE; Fig. 5). This indicates that the IR model can accommodate the heterogeneous rates required by an Early Cretaceous radiation of crown‐angiosperms. That it does not recover an Early Cretaceous radiation of crown‐angiosperms, unless forced to do so, is a reflection of the absence of evidence to preclude a pre‐Cretaceous origin of crown‐angiosperms. Indeed, it is perhaps ironic that the largest rate change inferred from both the SA and SE calibration strategies occurs on the eudicot crown (Fig. 5), the minimum age constraint on which informs the minimum age of all subtending clades through to the angiosperm crown. Thus, in effect, it is the fossil constraint on the minimum age of crown‐eudicots which, more than anything else, implies a pre‐Cretaceous origin of crown‐angiosperms.

It is not clear how a more precise evolutionary timescale for angiosperm diversification may be leveraged without sacrificing accuracy. It is likely that the addition of more sequence data will increase the precision of the divergence time estimates, but significant residual uncertainty will remain, associated with the fossil calibrations, which no amount of sequence data can overcome (Yang & Rannala, 2006). Increased taxon sampling is unlikely to increase precision on the age of crown‐angiosperms as there are no fundamental lineages immediately above or below this node that are absent from our dataset. It is possible that alternative approaches to molecular clock calibration, such as tip calibration, might yield greater precision. These rely on molecular and morphological data and their respective models of evolution, allowing fossil species to be included alongside their living relatives, calibrating the analysis directly, based on their age, rather than the inferred age of an ancestral node (Pyron, 2011; Ronquist *et al*., 2012). Unfortunately, this approach usually results in clade age estimates that are even older than those estimated using conventional node calibrations (O'Reilly *et al*., 2015; O'Reilly & Donoghue, 2016) and is very sensitive to the branching model used to specify the prior on times (dos Reis *et al*., 2016; Zhang *et al*., 2016).

The only practical and tractable approach to improving the precision of divergence time estimates for early angiosperm evolution can be in reducing the uncertainty associated with the fossil calibrations, and therefore with the interpretation of the fossil record. Demonstration that pre‐Cretaceous seed plant macrofossils fail to exhibit conclusive evidence of crown‐angiosperm affinity (Herendeen *et al*., 2017) is not the same as demonstrating that they are not crown‐angiosperms, or that crown‐angiosperms diverged in the Cretaceous. This false logic is invariably based on the absence of evidence of ‘key characters’ rather than evidence of their absence, at least as likely a consequence of incomplete fossilization and observation, as implicit assertion that they are primitively absent. This taphonomic artefact is widely appreciated to result in fossil taxa appearing more primitive than they are (Sansom *et al*., 2010), resulting in divergence time underestimates (Sansom & Wills, 2013; Donoghue & Yang, 2016). Furthermore, perceptions of ‘key characters’ have invariably been formulated within the increasingly out‐moded parsimony‐based phylogenetic framework (Wright & Hillis, 2014; O'Reilly *et al*., 2016, 2017; Puttick *et al*., 2017) used to infer both seed plant relationships and the phylogenetic distribution of characters. Symptomatically, much of the controversy over seed plant relationships is rooted in the false precision of parsimony‐based phylogenetic analyses of morphological characters (O'Reilly *et al*., 2016, 2017; Puttick *et al*., 2017). At the least, the hypotheses of character evolution used to discriminate stem‐ and crown‐angiosperm fossil taxa should be reviewed within a probabilistic framework that can better accommodate the uncertainty associated with such inference. However, it may be more appropriate to reconsider the phylogenetic position of critical fossil taxa using likelihood models of character evolution to accommodate phylogenetic uncertainty (Wright & Hillis, 2014; O'Reilly *et al*., 2016; Puttick *et al*., 2017) as discrimination between a stem‐ and crown‐angiosperm affinity of all pre‐Cretaceous claims may be the only way in which molecular estimates for the origin of flowering plants are going to achieve accuracy and precision.

Nonetheless, despite the uncertainty in the timing of the origin of crown‐angiosperms, the results of our analyses allow us to reject the hypothesis that crown‐angiosperms originated in the Cretaceous and, as such, allow us to reject the extreme hypothesis of KTR, or an explosive diversification of flowering plants fully within the Cretaceous (Cascales‐Miñana *et al*., 2016). However, our results remain compatible with a more general hypothesis of a KTR, in that diversification of the major groups of angiosperms occurred later (150–100 Ma), contemporaneous with the explosive diversification of derived lineages of insects (Misof *et al*., 2014), seed‐free land plants (Schneider *et al*., 2004; Feldberg *et al*., 2014; Laenen *et al*., 2014) and within the interval in which the fossil record reflects flowering plants to have risen to ecological dominance in terrestrial communities.

### Conclusions

From their first application, molecular clock methods have predicted a protracted cryptic history of crown‐angiosperms, establishing one of the most iconic and enduring of controversies between palaeontological and molecular biological approaches to establishing evolutionary timescales. Despite their ability to accommodate uncertainty in calibration dates and evolutionary rates, Bayesian approaches have only reinforced this polarization in perception of the extent of angiosperm evolutionary history.

In large part, the discrepancy between these approaches is an artefact of false precision on both sides. In molecular divergence time estimation, previous studies have failed to explore the implications of experimental variables and have inaccurately summarized the broad probabilistic estimates of clade age with undue precision. Similarly, interpretations of the palaeobotanical record have not fully recognized intrinsic evidence of its shortcomings as an archive of evolutionary history, namely the earliest conclusive angiosperm records are of the derived eudicots, the rock record in which the palaeobotanical record is entombed affords only an environmentally heterogeneous temporal archive and the affinities of early and pre‐Cretaceous angiosperm‐like fossils remain poorly constrained. As such, rejection of a pre‐Cretaceous origin of crown‐angiosperms is based on an absence of conclusive evidence of presence.

Our analyses controlled for the limitations of previous studies (e.g. low taxon sampling, limited sequence data, insufficient outgroup lineages failure to control for phylogenetic uncertainty, or a combination of these shortcomings), while also controlling for several sources of uncertainty. The ensuing timescale does not allow us to discriminate between interpretations of a long vs short cryptic interval of pre‐fossil crown‐angiosperm evolutionary history. Our results allow us to reject the conventional interpretation of a KTR; nevertheless, the diversification of speciose clades amongst crown‐angiosperms does appear to coincide with that of herbivores and pollinators and their predators, corroborating a more general hypothesis of a KTR. This underlines the power of the complementary nature of molecular and palaeontological data and approaches for inferring evolutionary timescales and establishing a deeper understanding of clade dynamics in deep time.

## Author contributions

J.B‐M., M.d.R., P.C.J.D. and Z.Y. conceived the project and designed the analysis. P.C.J.D. and H.S. compiled the fossil dataset for the calibration points. J.B‐M. prepared the datasets and carried out the analyses. J.B‐M. and P.C.J.D. wrote the main draft of the manuscript. All authors contributed to the interpretation of results and worked on the manuscript.

## Supporting information

Please note: Wiley Blackwell are not responsible for the content or functionality of any Supporting Information supplied by the authors. Any queries (other than missing material) should be directed to the *New Phytologist* Central Office.

**Fig. S1** Maximum likelihood (ML) phylogenetic tree from plastid 1^st^–2^nd^ codon positions for 643 taxa.
**Fig. S2** Maximum likelihood (ML) phylogenetic tree from mitochondrial 1^st^–2^nd^ codon positions for 515 taxa.
**Fig. S3** Maximum likelihood (ML) phylogenetic tree from nuclear RNA genes for 540 taxa.
**Fig. S4** RAxML phylogenetic tree from the 83 genes and 644 taxa of tracheophytes.
**Fig. S5** Chronogram of 644 taxa of tracheophytes (from SA‐IR‐3P).
**Fig. S6** Calibration, prior and posterior densities for 52 calibrated nodes in the tree and for the five calibration strategies.
**Table S1** List of genes included in the dataset
**Table S2** Basic information of data partitions
**Table S3** Summary of fossil calibrations used in this study in million years before the present
**Table S4** The 95% high posterior density (HPD) limits of posterior divergence times for selected nodes in the vascular plant tree under the five calibration strategies in millions of years before the present
**Table S5** The 95% high posterior density (HPD) limits of posterior divergence times, in millions of years before the present, for selected nodes in the vascular plant tree under different partition strategies, autocorrelated rates (AR) model, birth–death parameters and excluding lycophytes and ferns
**Notes S1** Justification of fossil calibrations.
