# Supplementary material for: Constraining uncertainty in the timescale of angiosperm evolution and the veracity of a Cretaceous Terrestrial Revolution
Source: New Phytol. 2018 Feb 5;218(2):819–34. doi: 10.1111/nph.15011 (PMC6055841; doi:10.1111/nph.15011)
Supplement: Supplementary file 1 — Fig. S1 Maximum likelihood (ML) phylogenetic tree from plastid 1st–2nd codon positions for 643 taxa. Fig. S2 Maximum likelihood (ML) phylogenetic tree from mitochondrial 1st–2nd codon positions for 515 taxa. Fig. S3 Maximum likelihood (ML) phylogenetic tree from nuclear RNA genes for 540 taxa. Fig. S4 RAxML phylogenetic tree from the 83 genes and 644 taxa of tracheophytes. Fig. S5 Chronogram of 644 taxa of tracheophytes (from SA‐IR‐3P). Fig. S6 Calibration, prior and posterior densities for 52 calibrated nodes in the tree and for the five calibration strategies. Table S1 List of genes included in the dataset Table S2 Basic information of data partitions Table S3 Summary of fossil calibrations used in this study in million years before the present Table S4 The 95% high posterior density (HPD) limits of posterior divergence times for selected nodes in the vascular plant tree under the five calibration strategies in millions of years before the present Table S5 The 95% high posterior density (HPD) limits of posterior divergence times, in millions of years before the present, for selected nodes in the vascular plant tree under different partition strategies, autocorrelated rates (AR) model, birth–death parameters and excluding lycophytes and ferns Notes S1 Justification of fossil calibrations. [file NPH-218-819-s001.pdf]

Constraining uncertainty in the timescale of angiosperm evolution and the veracity of a Cretaceous Terrestrial Revolution

Jose Barba-Montoya, Mario dos Reis, Harald Schneider, Philip C. J. Donoghue and Ziheng Yang

SUPPLEMENTARY FIGURES 1-6 (pages 2-15)

SUPPLEMENTARY TABLES S1-S3 (pages 16-22)

NOTES S1 – JUSTIFICATION OF FOSSIL CALIBRATIONS (pages 23-44)

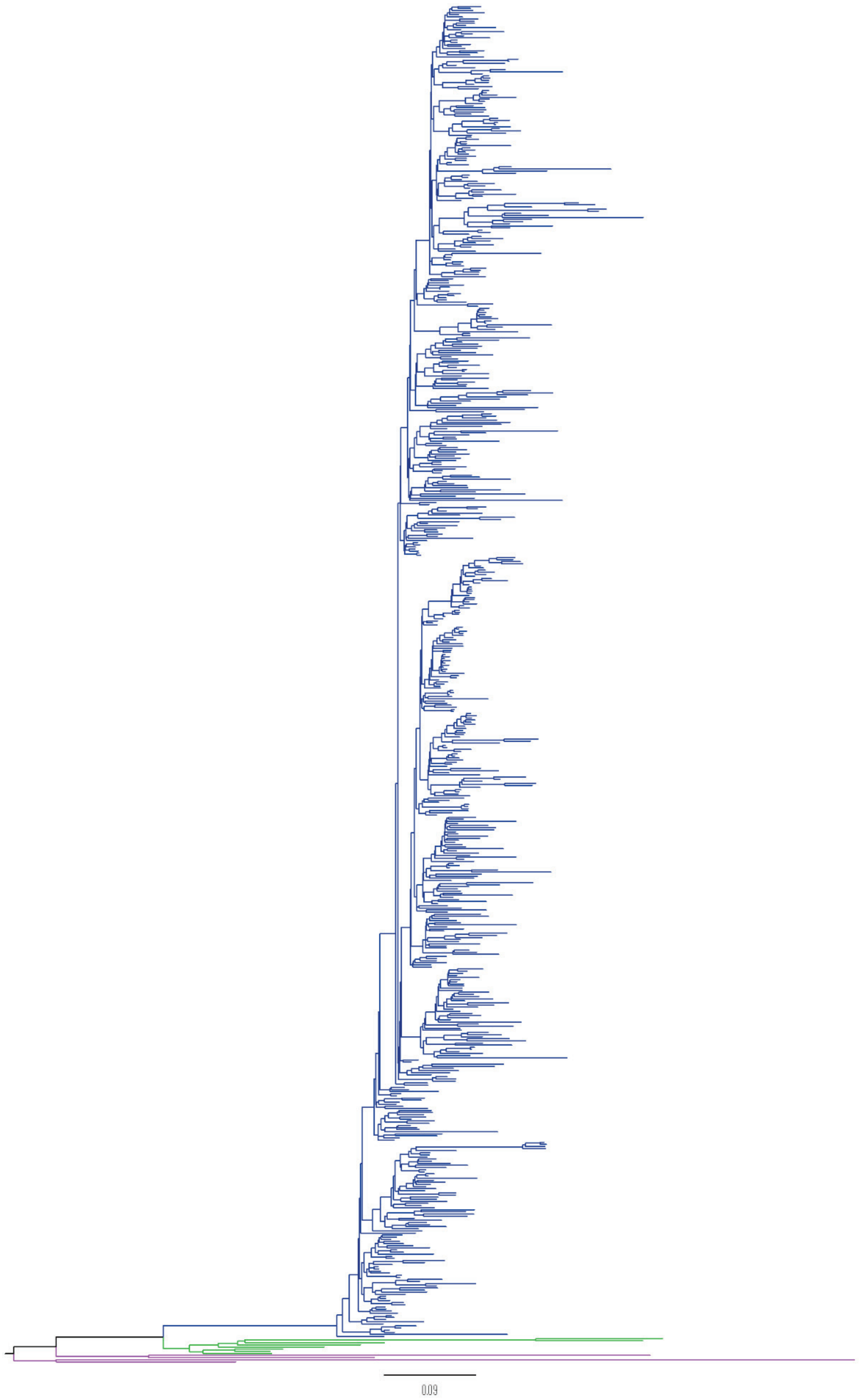

Figure S1. ML Phylogenetic tree from plastid 1st-2nd codon positions for 643 taxa. The Phylogeny was estimated using the GTR+GAMMA model and 1 partition (plastid 1st-2nd). The major lineages of tracheophytes are highlighted: lycophytes and ferns (purple), acrogymnosperms (green) and angiosperms (blue).

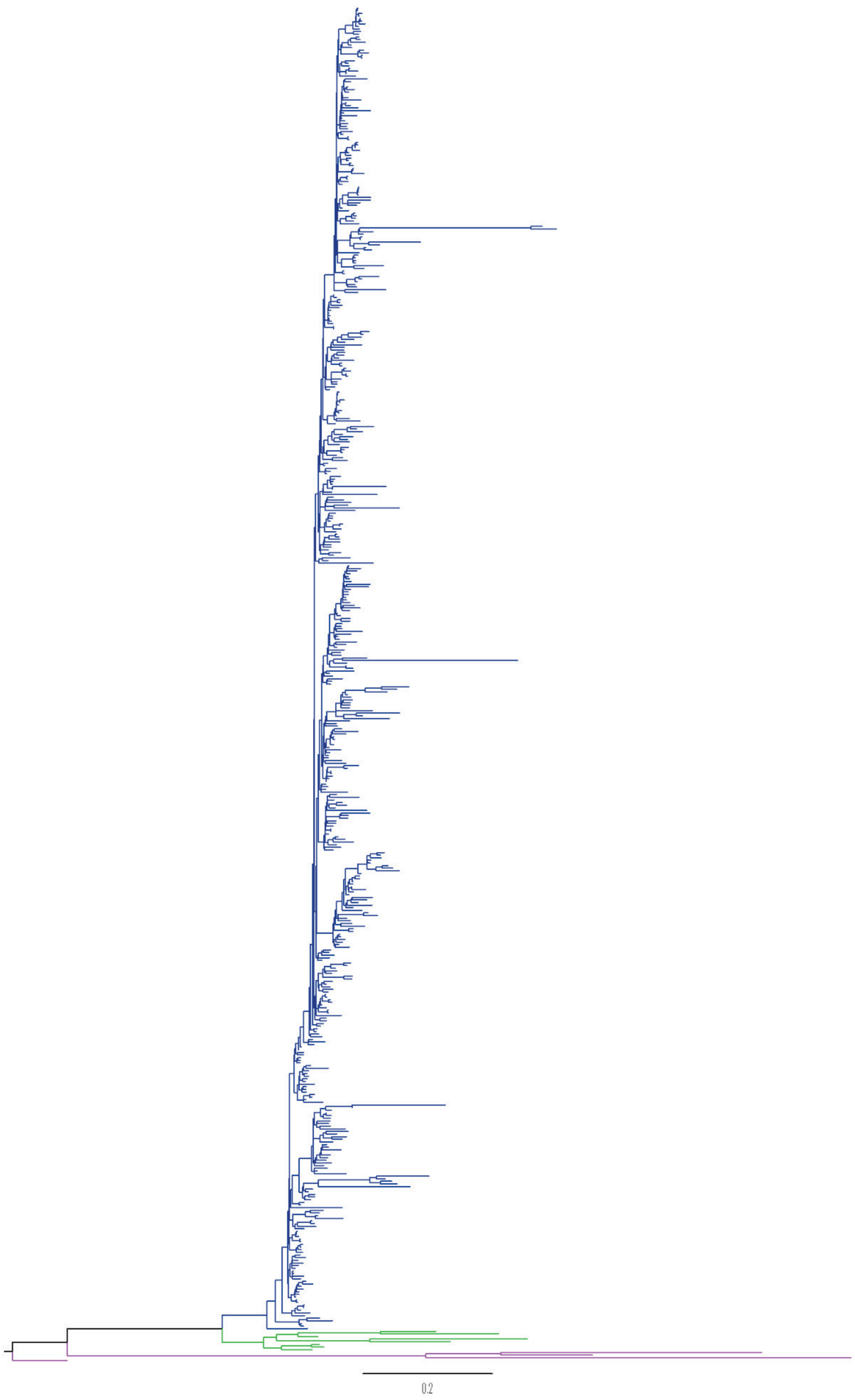

Figure S2. ML Phylogenetic tree from mitochondrial 1st-2nd codon positions for 515 taxa. The Phylogeny was estimated using the GTR+GAMMA model and 1 partition (mitochodrial 1st-2ndThe major lineages of tracheophytes are highlighted: lycophytes and ferns (purple), acrogymnosperms (green) and angiosperms (blue).

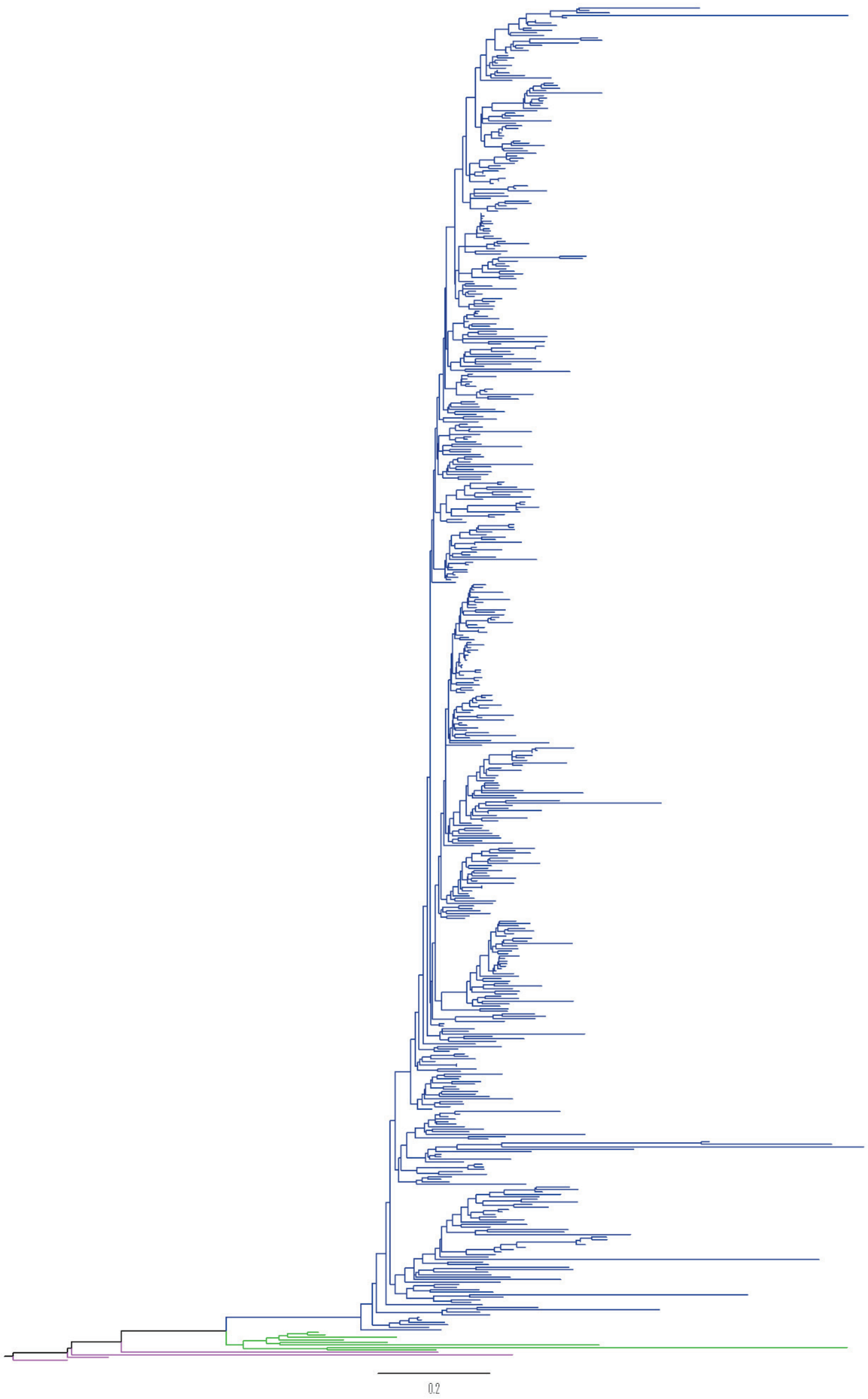

Figure S3. ML Phylogenetic tree from nuclear RNA genes for 540 taxa. The Phylogeny was estimated using the GTR+GAMMA model and 1 partition (nuclear RNA genes). The major lineages of tracheophytes are highlighted: lycophytes and ferns (purple), acrogymnosperms (green) and angiosperms (blue).

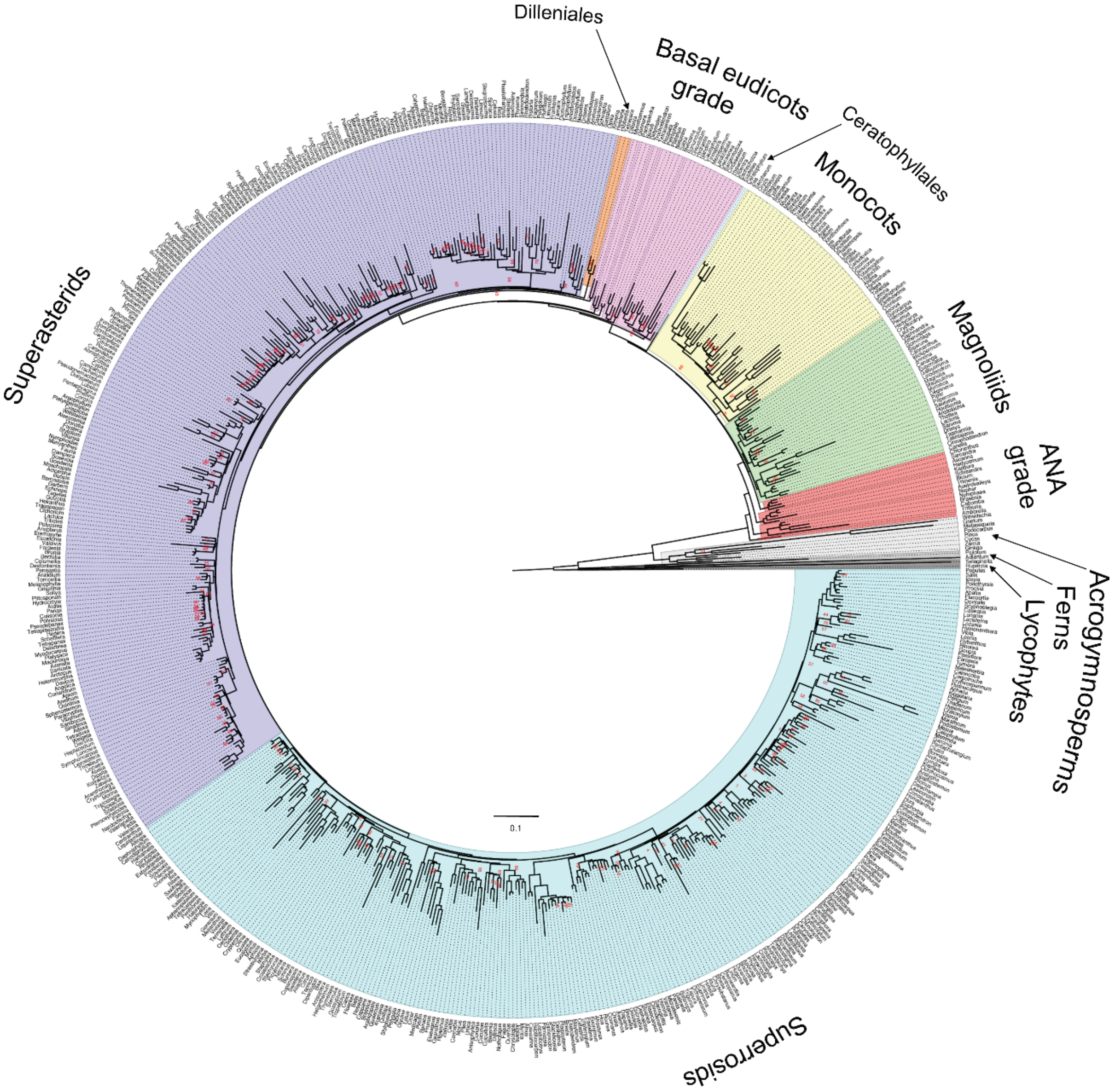

Figure S4. RAxML Phylogenetic tree from the 83 genes and 644 taxa of tracheophytes. The Phylogeny was estimated using the GTR+GAMMA model, using five partitions (plastid 1st-2nd, plastid 3rd, mitochondrial 1st-2nd, mithochondrial 3rd and nuclear RNA genes). Bootstrap support values of 100 % were excluded, while other support values < 100 % are reported directly with red numbers on nodes. The major lineages of tracheophytes and major groups of angiosperms are highlighted: lycophytes (dark grey) and ferns (middle grey), acrogymnosperms (pale grey), ANA grade (red), magnoliids (green), monocots (yellow), Ceratophyllales (pale blue), basal eudicots grade (pink), Dilleniales (orange), superasterids (purple) and superrosids (blue).

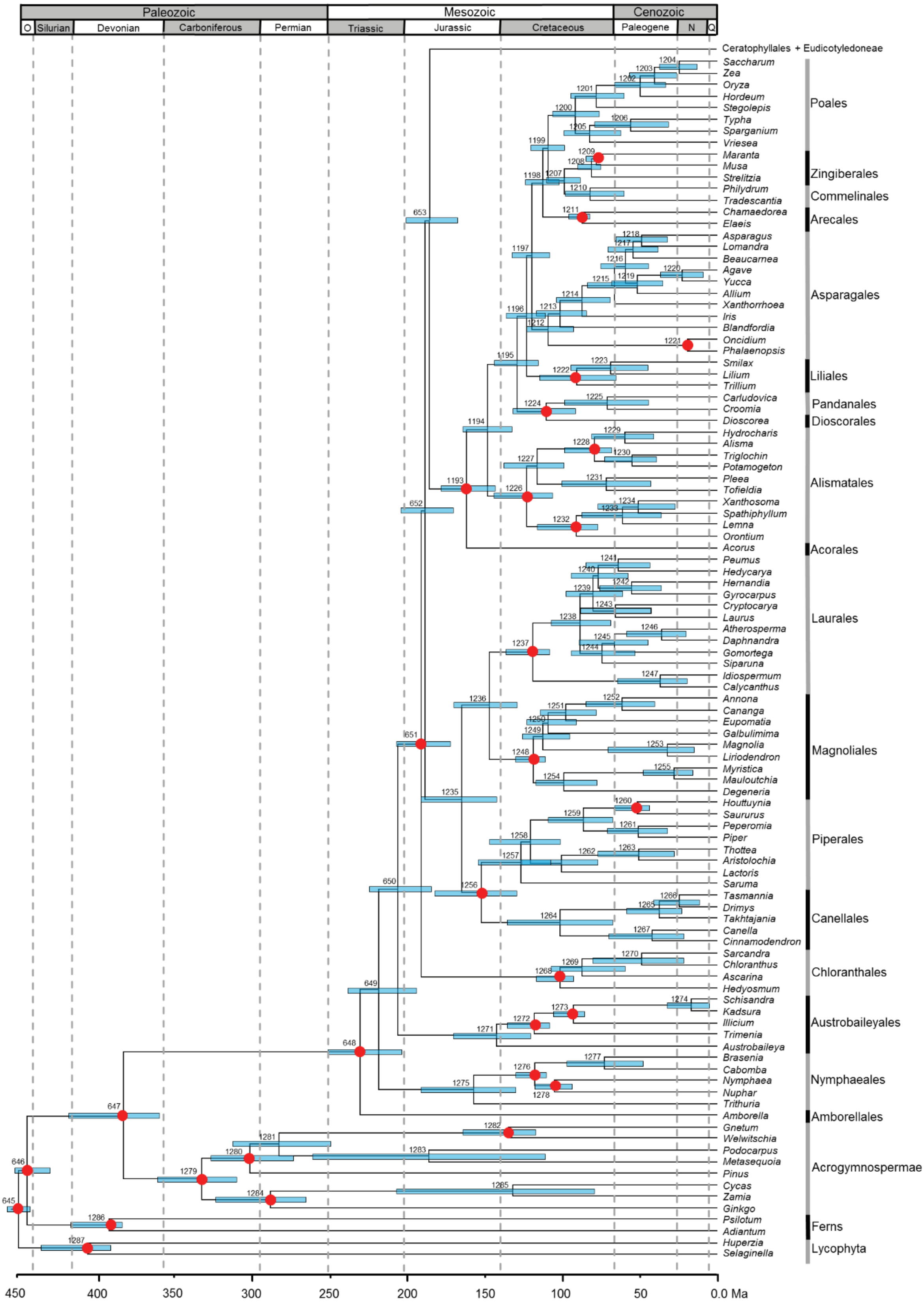

Figure S5. Chronogram of 644 taxa of tracheophytes (from SA-IR-3P). Blue bars represent the 95% HPD credibility intervals for the node ages. Red dots represent calibrated nodes. Divergence times were estimated using calibration strategy A, HKY85+Γ5 substitution model, independent rates model, with the 83 genes subdivided into three partitions: (1) 1st and 2nd codon positions for plastid genes; (2) 1st and 2nd codon positions for mitochondrial genes; and (3) nuclear RNA genes.

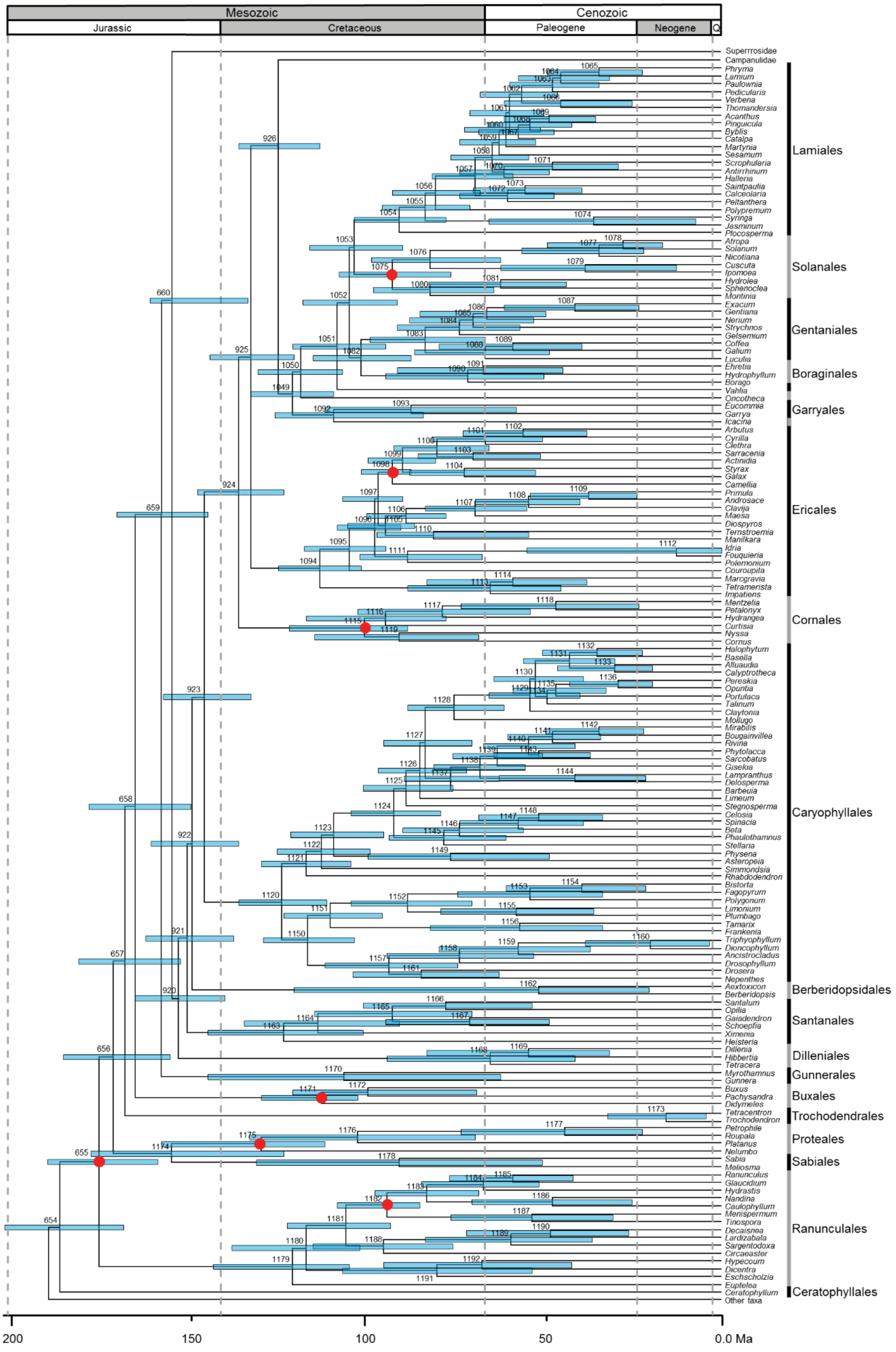

Figure S5.1. Chronogram of 644 taxa of tracheophytes (from SA-IR-3P). Continued

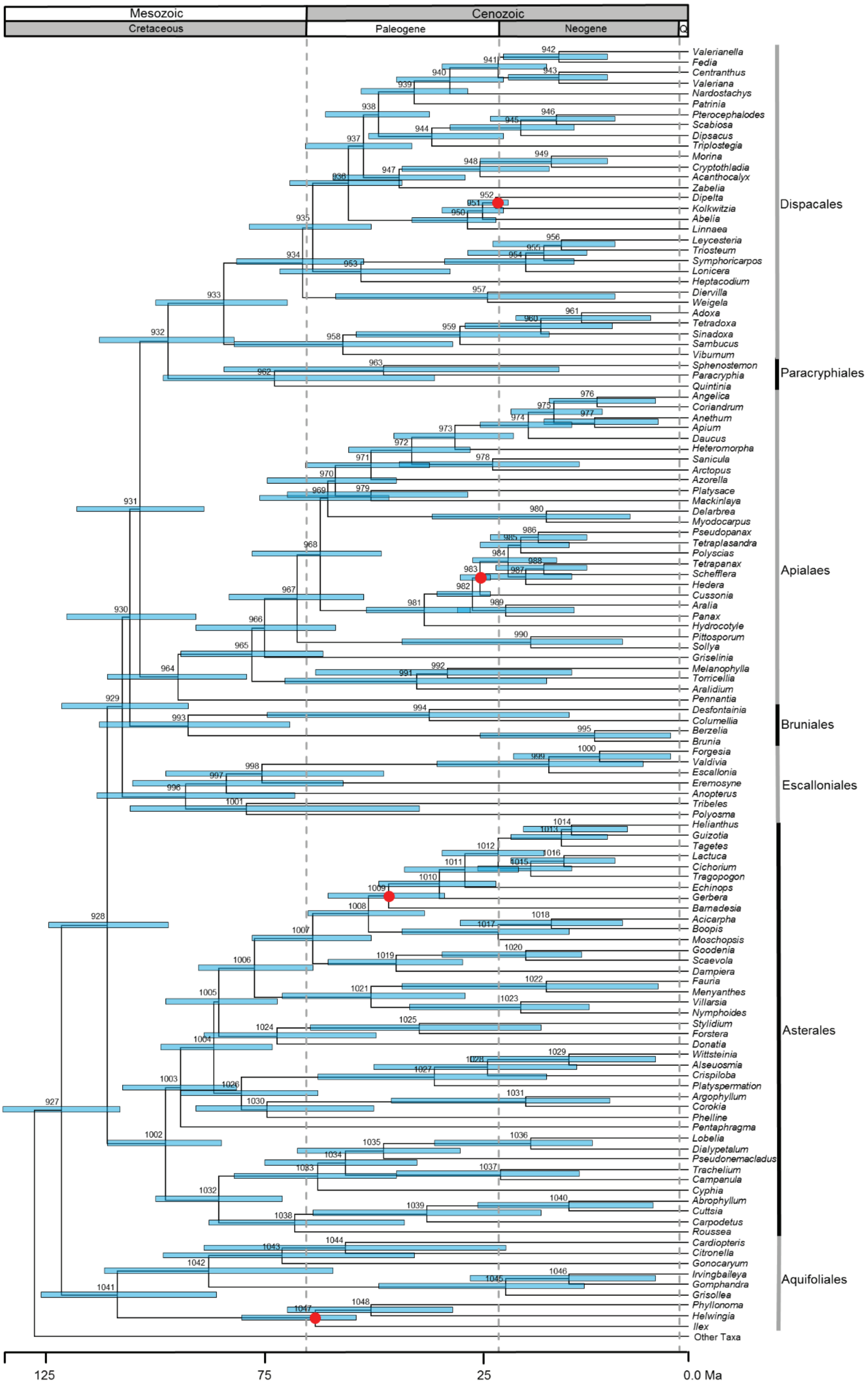

Figure S5.2. Chronogram of 644 taxa of tracheophytes (from SA-IR-3P).

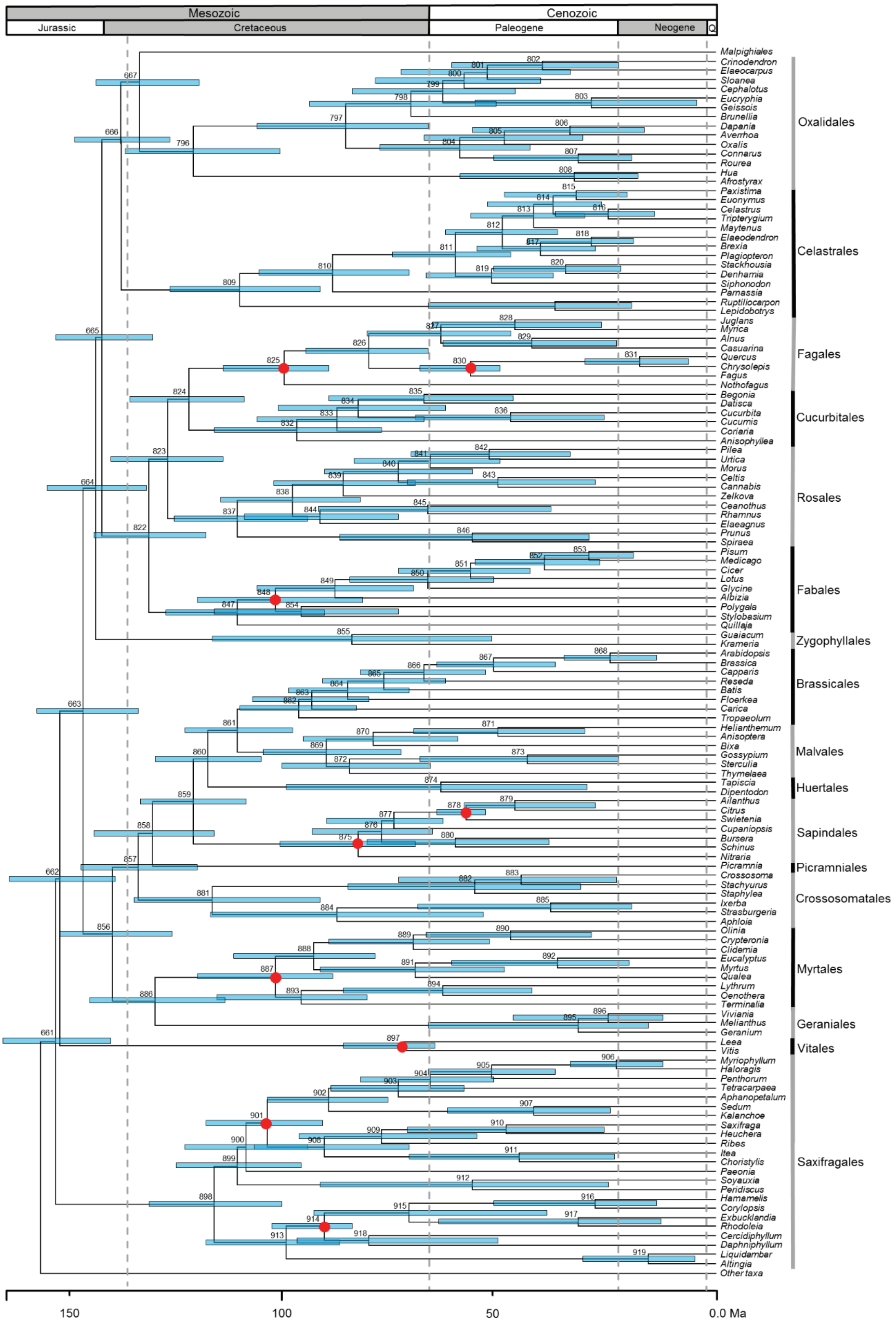

Figure S5.3. Chronogram of 644 taxa of tracheophytes (from SA-IR-3P).

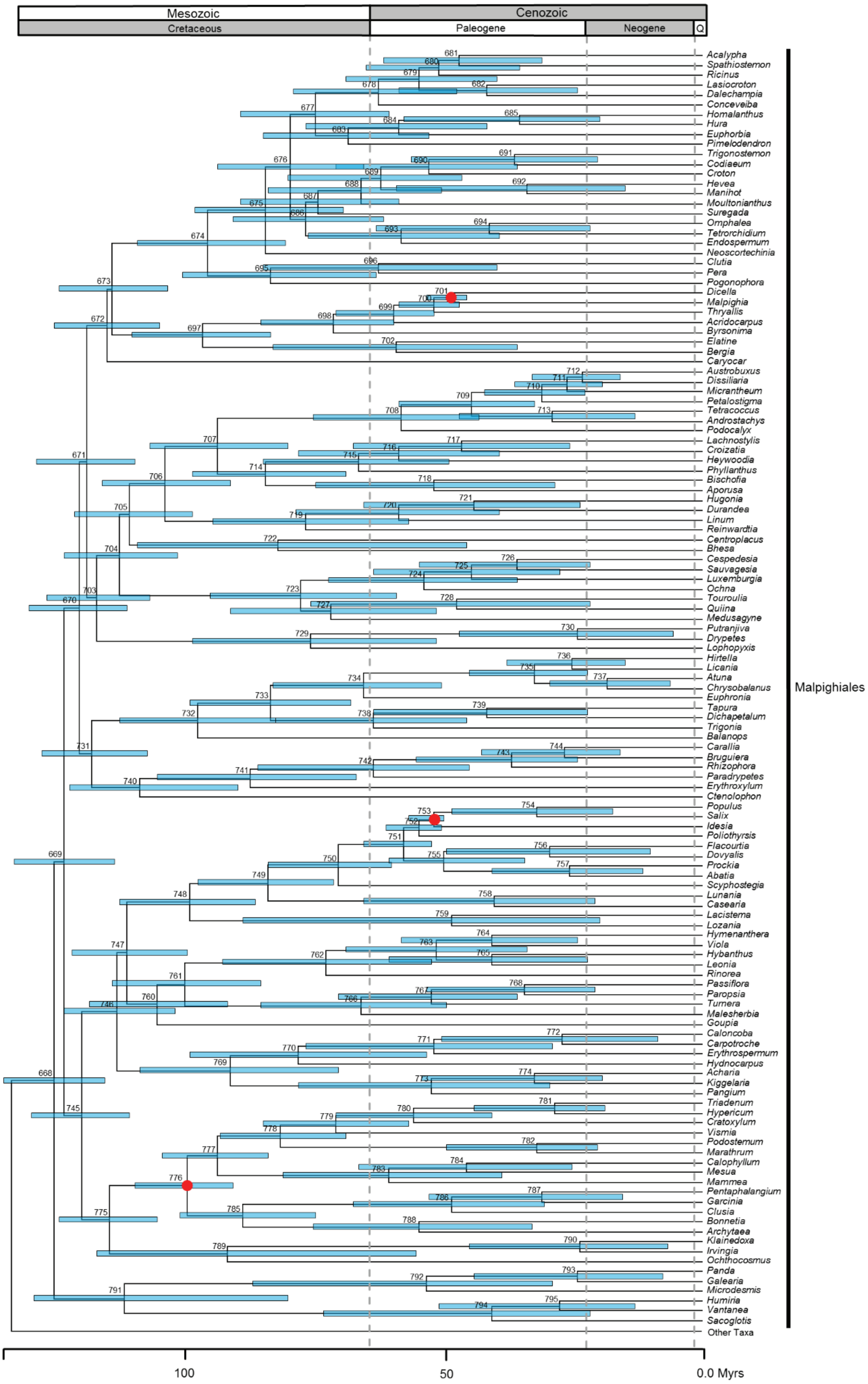

Figure S5.4. Chronogram of 644 taxa of tracheophytes (from SA-IR-3P).

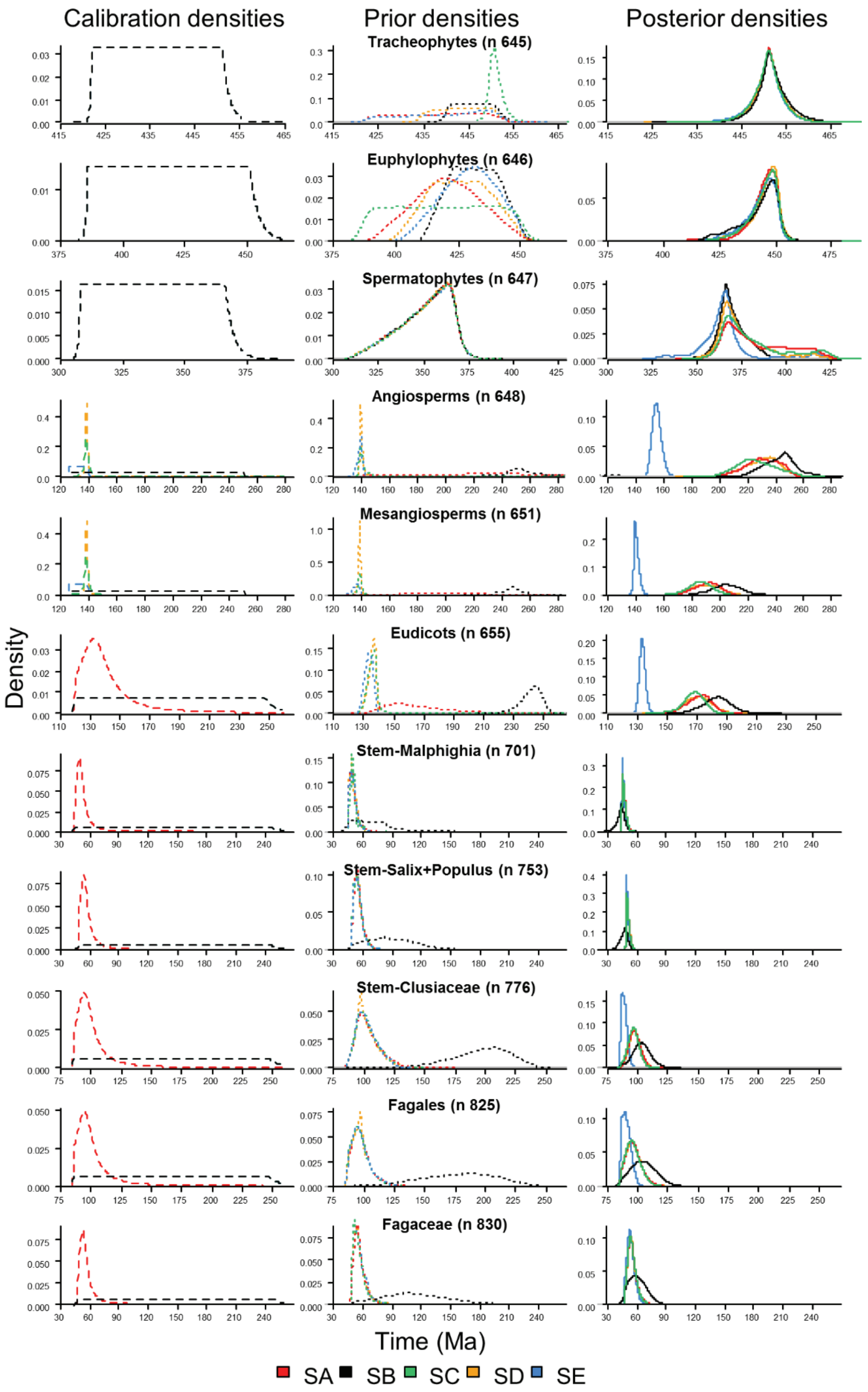

Figure S6. Calibration, prior and posterior densities for 52 calibrated nodes in the tree and for the five calibration strategies. Fossil calibrations are shown as dashed lines, priors as doted lines and posterior as solid lines. Nodes are numbered as in Supplementary Fig. 1. Estimates were obtained using the HKY85+Γ5 substitution model, independent rates model, with the 83 genes subdivided into three partitions: (1) 1st and 2nd codon positions for plastid genes; (2) 1st and 2nd codon positions for mitochondrial genes; and (3) nuclear RNA genes. Colouring relates to the calibration strategy (SA, SB, SC, SD, SE) as in Figure 1.

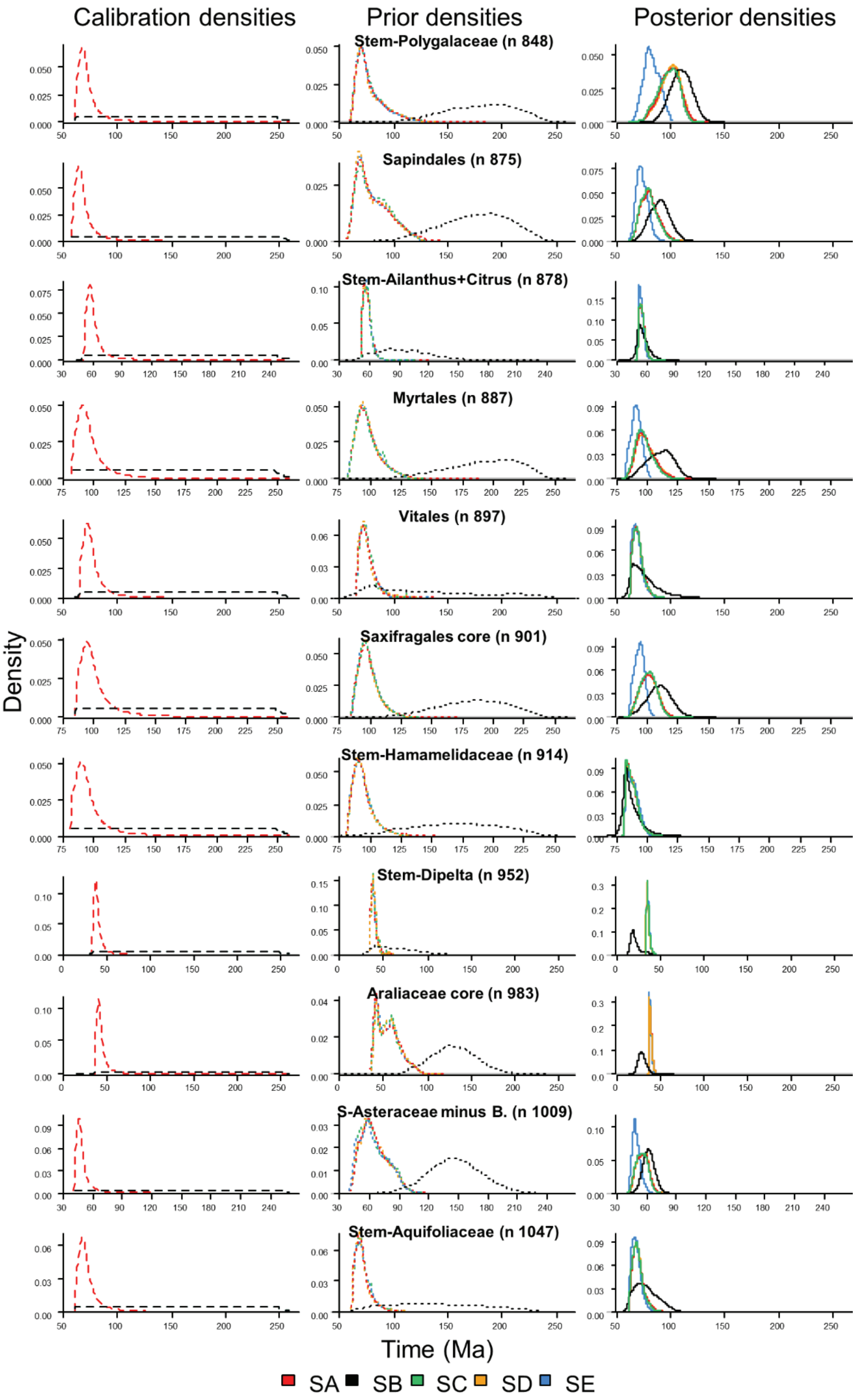

Figure S6.1. Calibration, prior and posterior densities for 52 calibrated nodes in the tree and for the 5 calibration strategies. Continued.

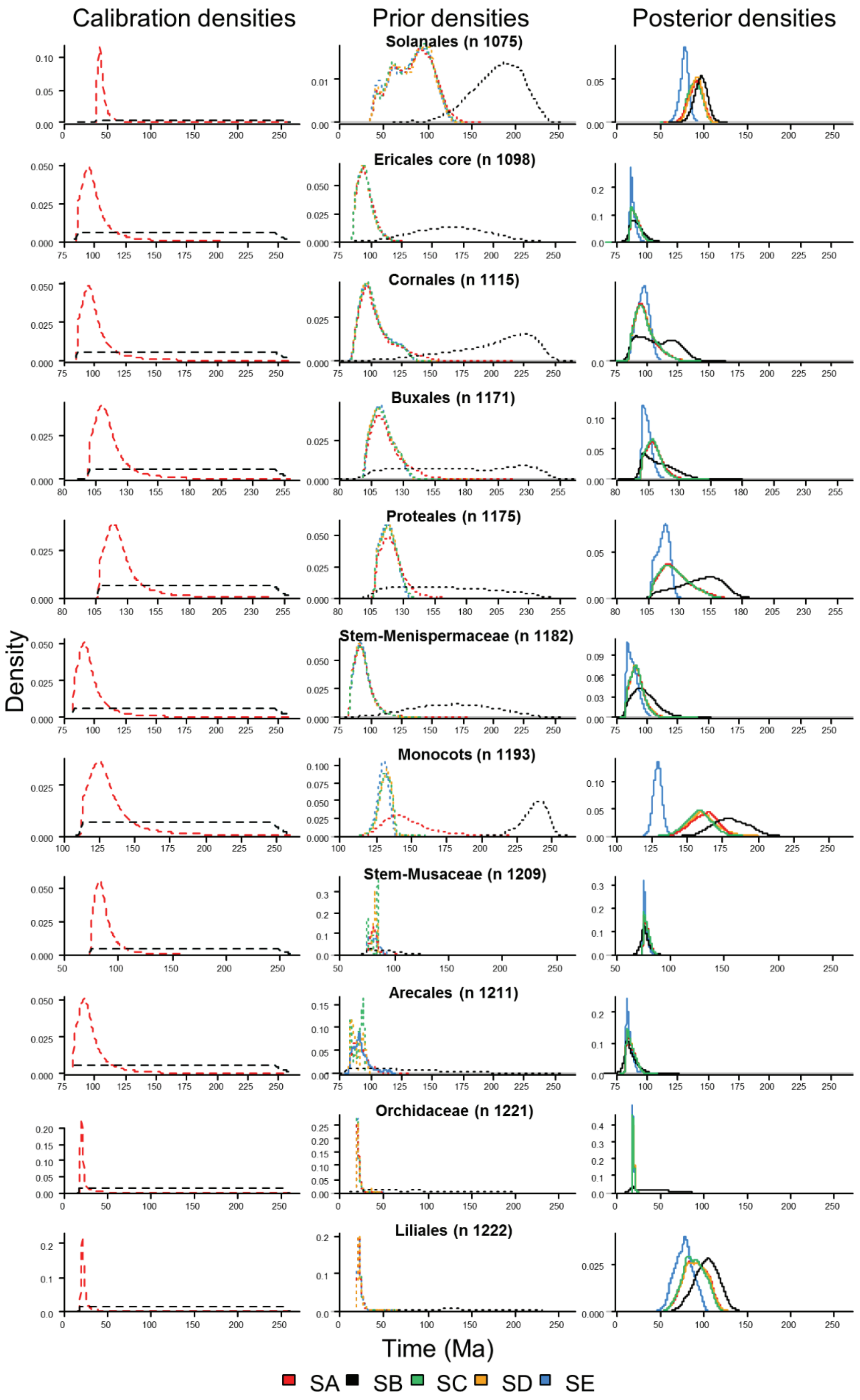

Figure S6.2. Calibration, prior and posterior densities for 52 calibrated nodes in the tree and for the 5 calibration strategies. Continued.

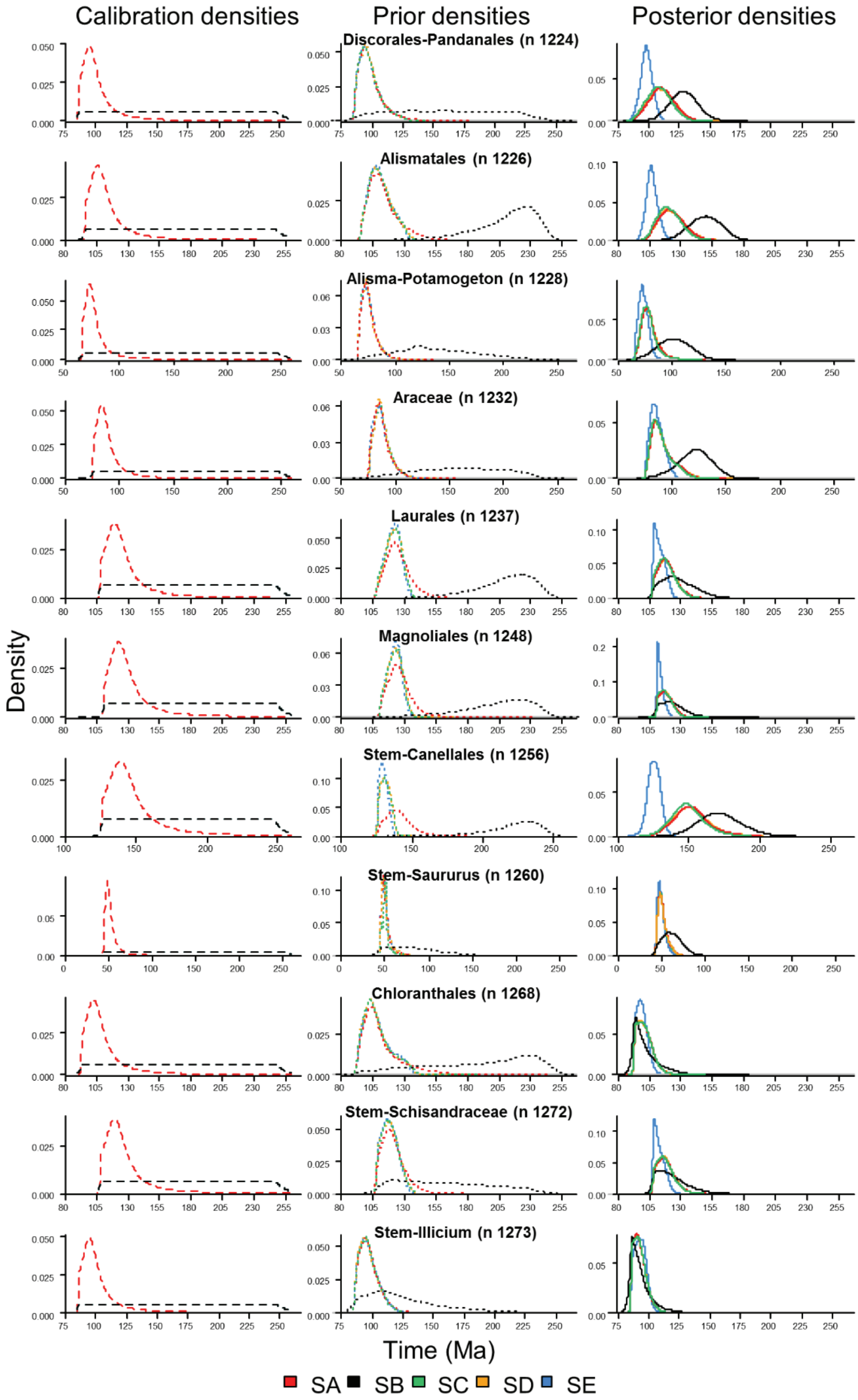

Figure S6.3. Calibration, prior and posterior densities for 52 calibrated nodes in the tree and for the 5 calibration strategies. Continued.

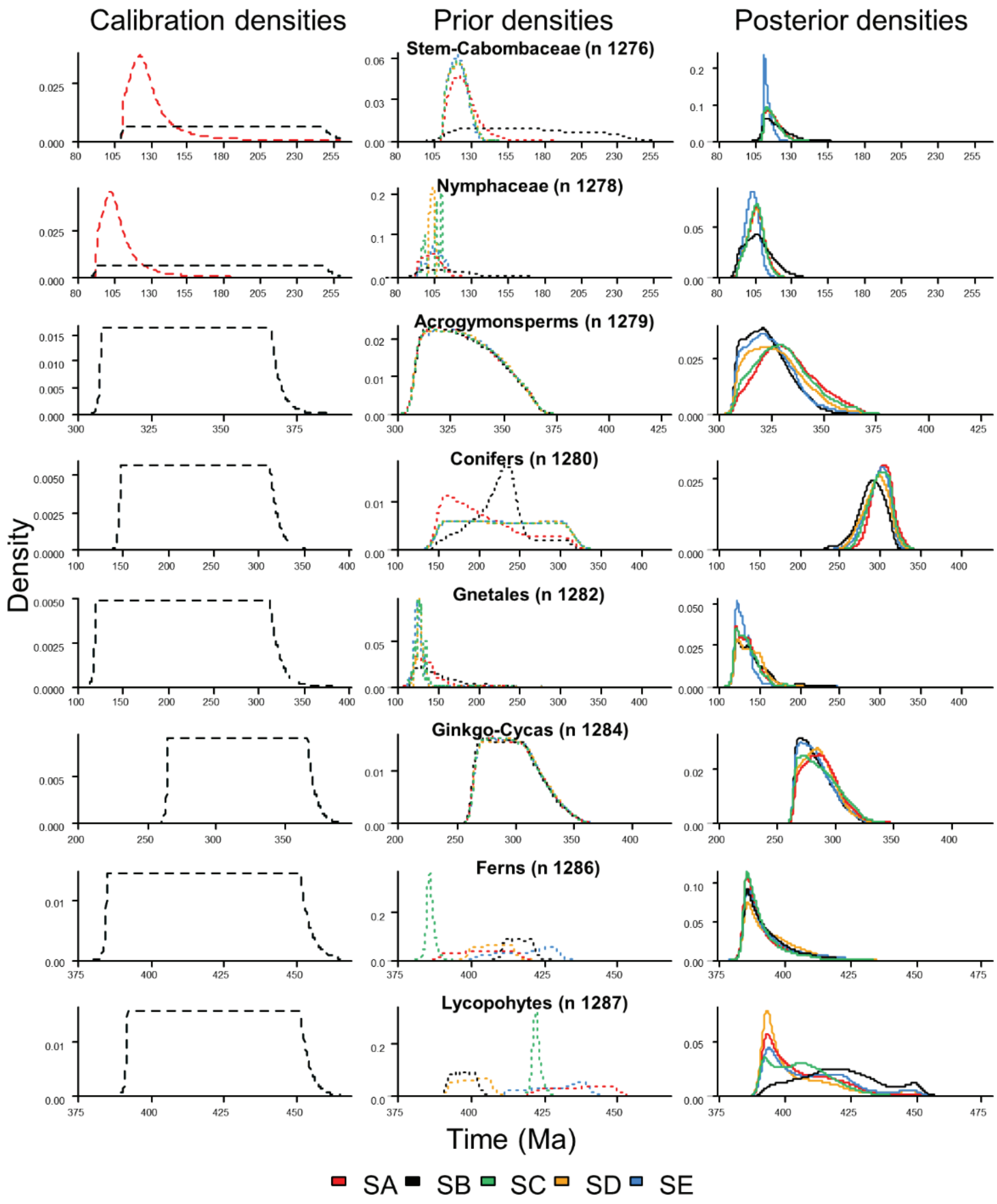

Figure S6.4. Calibration, prior and posterior densities for 52 calibrated nodes in the tree and for the 5 calibration strategies. Continued.

Table S1. List of genes included in the dataset. Tree lengths for each gene were obtained using RAxML under GTR+GAMMA model.

gene	No. taxa	# sites	tree length	gene	No. taxa	# sites	tree length
Plastid Genes				Plastid Genes			
accD	83	1200	7.03	*psbJ*	109	120	6.26
atpA	110	1518	6.98	*psbK*	110	126	5.31
atpB	559	1548	21.24	*psbL*	108	111	2.9
atpE	108	387	7.25	*psbM*	107	102	4.47
atpF	105	543	7.49	*psbZ*	106	183	5.06
atpH	110	240	5.62	*rbcL*	627	1497	43.26
atpI	109	741	7.34	*rpl14*	109	363	7.04
ccsA	109	672	10.56	*rpl16*	110	393	8.86
cemA	108	654	7.42	*rpl2*	100	813	4.49
clpP	102	582	12.32	*rpl20*	108	336	9.49
infA	77	222	6.42	*rpl22*	94	318	10.21
matk	588	2421	50.09	*rpl23*	99	270	3.72
ndhA	102	1086	8.97	*rpl32*	102	129	10.96
ndhB	98	1530	3.25	*rpl33*	108	195	7.24
ndhC	100	360	5.76	*rpl36*	110	108	6.58
ndhD	102	1488	9.71	*rpoA*	108	978	9.61
ndhE	103	300	8.19	*rpoB*	108	3207	8.23
ndhF	509	2928	43.81	*rpoC1*	102	2157	8.08
ndhG	101	525	9.22	*rpoC2*	398	8604	28.96
ndhH	101	1173	6.9	*rps11*	109	414	11.43
ndhI	101	465	6.16	*rps12*	104	369	5.28
ndhJ	100	468	5.55	*rps14*	109	294	7.51
ndhK	96	855	7.32	*rps15*	104	255	11.61
petA	108	891	8.72	*rps16*	95	234	7.35
petB	110	642	6.30	*rps18*	107	294	10.83
petD	108	471	6.41	*rps19*	109	273	8.57
petG	110	108	3.99	*rps2*	107	705	7.54
petL	102	183	3.44	*rps4*	370	984	16.63
petN	105	87	2.87	*rps7*	108	465	3.3
psaA	109	2235	6.24	*rps8*	110	390	8.74
psaB	109	2199	5.62	*ycf2*	97	3813	5.91
psaC	109	225	5.68	*ycf3*	108	504	5.74
psaI	107	108	7.15	*ycf4*	106	549	7.57
psaJ	108	123	6.58	**Mitochondrial genes**			
psbA	109	1056	4.18	*atp1*	378	1713	11.03
psbB/N/T/H	343	2073	16.16	*matr*	480	4236	10.85
psbC	109	1356	6.31	*nad5*	373	2073	5.60
psbD	109	1056	4.65	*rps3*	398	3171	21.05
psbE	110	243	5.12	**Nuclear RNA genes**			
psbF	109	117	2.97	*rDNA 18S*	501	1769	29.03
psbI	109	105	5.99	*rDNA 26S*	368	3396	37.04

Table S2. Basic information of data partitions.				
Partition	No. taxa	# sites	ML tree lenght	alpha
**Nucleotide partitions**				
Plastid 1st & 2nd	643	41021	22.541	0.519
Plastid 3rd	643	20360	47.605	1.180
Mitochondrial 1st & 2nd	515	5690	13.666	0.473
Mitochondrial 3rd	515	2878	22.82	0.776
Nuclear rDNA	540	5081	39.546	0.372
All partitions concatenated as a single partition	644	75030	29.41	0.486
**Amino acid partitions**				
Plastid proteins	643	21579	46.563	0.521
Mitochondrial proteins	515	3731	23.003	0.610

Tree length (sum of branch lengths) and gamma shape parameter (α) were estimated using RAxML under the GTR+GAMMA model for nucleotide partitions, the CPREV+GAMMA for plastid proteins and WAG+GAMMA for mitochondrial proteins.

Table S3. Summary of Fossil calibrations used in this study in million years before the present.

Node	Clade	Stem/Crown	Minimum divergence time (Ma)	Maximum divergence time (Ma)
645	Tracheophytes	Crown	422 (†*Zosterophyllum* sp)	451 (oldest occurrence of trilete spores)
646	Euphyllophytes	Crown	385.57 (†*Rellimia thomsonii)*	451 (oldest occurrence of trilete spores)
647	Spermatophytes	Crown	308.14 (†*Cordaites iowensis*)	365.63 (base of Vco zone which contains the first seeds)
648	Angiosperms	Crown	125.9 (tricolpate pollen)	247.3 (sediments below the oldest occurrence of angiosperm like pollen which are devoid of such pollen)
651	Mesangiosperms	Crown	125.9 (tricolpate pollen)	247.3 (sediments below the oldest occurrence of angiosperm like pollen which are devoid of such pollen)
655	Eudicots	Crown	119.6 (†*Hyrcantha decussata*)	—
701	*Malphigia*	Stem	44.83 (†*Perisyncolporites pokornyi*)	—
753	*Salix* plus *Populus*	Stem	48.57 (†*Pseudosalix handleyi*)	—
776	Clusiaceae	Stem	85.8 (†*Paleoclusia chevaliery*)	—
825	Fagales	Crown	85.8 (†*Nothofagidites senectus*)	—
830	Fagaceae	Crown	47.6 (†*Fagus langevinii*)	—
848	Polygalaceae	Stem	61.6 (†*Paleosecuridaca curisii*)	—
875	Sapindales	Crown	59.24 (†*Dipteronia brownii*)	—
878	*Ailanthus* plus *Citrus*	Stem	51.83 (†*Ailanthus confucii*)	—
887	Myrtales	Crown	83.3 (†*Esqueiria futabensis*)	—
897	Vitales	Crown	65.5 (†*Indovitis chitaleyae)*	—
901	Saxifragales core	Crown	85.8 (†*Divisestylus brevistamineus* and †*D. longistamineus*)	—
914	Hamamelidaceae	Stem	82 (†*Androdecidua endressii*)	—
952	*Dipelta*	Stem	33.7 (†*Diplodipelta reniptera*)	—
983	Araliaceae core	Crown	37.3 (†*Dendropanax eocenensis*)	—
1009	Asteraceae minus *Barnadesia*	Stem	41.5 (†*Tubulifloridites antipodica*)	—
1047	Aquifoliaceae	Stem	61.6 (†*Ilex hercynica*)	—
1075	Solanales	Crown	37.3 (†*Solanites crassus*)	—
1098	Ericales core	Crown	85.8 (†*Paleoenkianthus sayrevillensis*)	—
1115	Cornales	Crown	85.8 (†*Tylerianthus crossmanensis*)	—
1171	Buxales	Crown	100.1 (†*Spanomera marylandensis*)	—
1175	Proteales	Crown	107.59 (†*Sapindopsis variabilis*, †*Aquia brookensis* and †*Palatonocarpus brookensis*)	—
1182	Menispermaceae	Stem	83.41 (†*Prototinomiscium testudinarum* and †*P. vangerowii*)	—
1193	Monocots	Crown	112.6 (†*Liliacidites*)	—
1209	Musaceae	Stem	74.6 (†*Spirematospermum chandlerae*)	—
1211	Arecales	Crown	83.41 (†*Sabalites carolinensis*)	—
1221	Orchidaceae	Crown	17.82 (†*Meliorchis caribea*)	—
1222	Liliales	Crown	18.7 (†*Luzuriaga contortus*)	—
1224	Dioscoreales—Pandanales	Crown	85.8 (†*Mabelia connatifila*)	—
1226	Alismatales	Crown	96.24 (†*Mayoa portugallica*)	—
1228	*Alisma—Potamogeton*	Crown	66 (†*Cardstonia tolmanii*)	—
1232	Araceae	Crown	76 (†*Lysichiton austriacus*)	—
1237	Laurales	Crown	107.59 (†*Virginianthus calycanthoides* and †*Cohongarootonia hispida*)	—
1248	Magnoliales	Crown	110.87 (†*Schenkeriphyllum glanduliferum* and †*Endressinia brasiliana*,)	—
1256	Canellales	Stem	125.9 (†*Walkeripollis gabonensis*)	—
1260	*Saururus*	Stem	44.3 (†*Saururus tuckerae*)	—
1268	Chloroanthaeles	Crown	92.8 (†*Pennipolis sp.*)	—
1272	Schisandraceae	Stem	107.59 (†*Anacostia virginiensis*)	—
1273	*Illicium*	Stem	85.44 (†*Illiciospermum pusillum*)	—
1276	Cabombaceae	Stem	110.97 (†*Pluricarpellatia peltata*)	—
1278	Nymphaeaceae	Crown	92.8 (†*Monetianthus mirus*)	—
1279	Acrogymnospermae	Crown	308.14 (†*Cordaties iowensis*)	365.63 (base of Vco zone which contains the first seeds)
1280	Conifers	Crown	147 (†*Rissikia media*)	312.38 (sediments bearing †*Cordaites iowensis*)
1282	Gnetales	Crown	119.6 (†*Eoantha zherkihinii*)	312.38 (sediments bearing †*Cordaites iowensis*)
2284	*Ginkgo—Cycas*	Crown	264.7 (†*Crossozamia*)	365.63 (base of Vco zone which contains the first seeds)
1286	Ferns	Crown	384.71 (†*Ibyka amphikoma*)	451 (oldest occurrence of trilete spores)
1287	Lycophytes	Crown	392.1 (†*Leclercquia complexa*)	451 (oldest occurrence of trilete spores)

Nodes are numbered as in Figure S2. Fossil taxa are indicated by a dagger (†) before their names.

Table S4. The 95% HPD limits of posterior divergence times for selected nodes in the vascular plant tree under the 5 calibration strategies in millions of years before the present.

		SA-IR-3P		SB-IR-3P		SC-IR-3P		SD-IR-3P		SE-IR-3P		Composite	
Node	Clade	Min.	Max.	Min.	Max.	Min.	Max.	Min.	Max.	Min.	Max.	Min.	Max.
645*	Tracheophytes	444	458	444	460	443	458	443	458	442	458	442	458
646*	Euphylophytes	433	455	424	455	430	454	430	455	427	453	427	455
647*	Spermatophytes	360	418	356	387	359	422	356	413	340	418	340	422
648*	Angiosperms	206	253	219	266	201	255	203	256	149	162	149	256
651*	Mesangiosperms	173	208	183	225	168	203	171	210	138	145	138	210
655*	Eudicots	156	186	164	201	152	181	155	188	129	137	129	188
661	Superrosids	136	160	141	173	134	157	136	162	118	126	118	162
662	Rosids	135	159	140	172	133	155	135	160	117	125	117	160
668	**Malpighiales**	113	132	115	144	111	130	113	133	100	110	100	133
701*	Stem-*Malphighia*	44	51	35	53	44	51	45	51	44	51	44	51
753*	Stem-*Salix* plus *Populus*	48	55	38	55	48	55	48	55	48	54	48	55
776*	Stem-Clusiaceae	88	107	88	117	88	105	88	107	85	93	85	107
796	**Oxidales**	100	134	105	144	99	132	100	134	86	110	86	134
809	**Celastrales**	89	123	93	133	88	122	89	123	78	104	78	123
825*	**Fagales**	86	108	86	123	86	108	86	108	85	98	85	108
830*	Fagaceae	48	64	46	78	48	64	48	64	48	62	48	64
832	**Cucurbitales**	76	113	80	121	75	111	77	113	65	96	65	113
837	**Rosales**	92	122	97	132	91	120	91	122	81	104	81	122
847	**Fabales**	88	124	98	136	87	122	89	124	77	103	77	124
848*	Stem-*Polygalaceae*	79	116	90	128	78	114	80	117	69	96	69	117
855	**Zygophyllales**	50	112	52	118	49	111	49	112	43	95	43	112
862	**Brassicales**	81	107	84	115	78	104	81	107	69	90	69	107
869	**Malvales**	71	102	76	110	71	101	72	103	61	88	61	103
874	**Huertales**	28	96	30	103	28	94	29	97	23	81	23	97
875*	**Sapindales**	66	96	71	107	66	95	66	96	64	84	64	96
878*	Stem-*Ailanthus* plus *Citrus*	52	62	47	69	52	62	52	62	52	60	52	62
881	**Crossosomatales**	90	132	97	144	89	130	91	133	81	111	81	133
887*	**Myrtales**	84	114	89	130	84	112	85	115	83	99	83	115
895	**Geraniales**	14	48	8	50	12	52	7	60	9	41	9	60
897*	**Vitales**	63	80	58	105	63	80	62	80	62	79	62	63
898	**Saxifragales**	97	126	100	142	97	125	97	127	94	111	94	127
901*	Saxifragales core	87	113	89	128	88	112	88	114	86	100	86	114
914*	Stem-Hamamelidaceae	81	97	75	104	81	97	81	97	81	97	81	97
921	Superasterids	135	159	140	171	133	156	135	161	116	126	116	161
924	Asterids	121	145	127	156	120	143	121	146	107	118	107	146
933	**Dispacales**	75	100	77	106	74	98	74	99	67	87	67	100
952*	Stem-Dipelta	34	40	13	36	34	40	34	40	34	40	34	40
962	**Paracryphiales**	50	100	52	105	49	99	49	99	43	88	43	100
964	**Apiales**	83	109	85	115	82	108	83	109	74	93	74	109
983*	Araliaceae core	37	42	21	38	37	42	37	42	37	42	37	42
993	**Bruniales**	76	111	78	117	75	110	76	112	67	95	67	112
996	**Escalloniales**	76	112	79	118	75	110	77	112	66	96	66	112
1002	**Asterales**	87	109	90	116	86	108	87	109	77	93	77	109
1009*	Stem-Asteraceae minus *Barnadesia*	45	67	51	74	45	66	45	67	42	58	42	67
1041	**Aquifoliales**	90	122	94	130	89	120	90	123	83	103	83	123
1047*	Stem-*Aquifoliaceae*	62	81	60	99	62	80	62	81	62	76	62	81
1054	**Lamiales**	76	101	81	108	75	100	76	101	67	86	67	101
1075*	**Solanales**	75	105	81	111	75	105	77	105	66	88	66	105
1083	**Gentaniales**	65	97	70	103	64	96	65	97	57	81	57	93
1090	**Boraginales**	49	93	51	97	48	91	49	93	43	79	43	93
1093	**Garryales**	58	111	62	118	57	109	59	111	50	94	50	111
1094	**Ericales**	99	122	100	130	99	121	99	122	94	105	94	122
1098*	Ericales core	85	98	83	103	85	98	85	98	85	93	85	98
1115*	**Cornales**	85	115	84	134	85	115	85	116	86	105	86	116
1120	**Caryophyllales**	109	133	112	142	107	131	109	133	94	109	94	133
1162	**Berberidopsidales**	18	111	16	118	18	111	18	110	17	100	17	111
1163	**Santanales**	100	142	107	153	99	140	101	143	87	115	87	143
1168	**Dilleniales**	39	91	44	96	39	88	40	93	33	74	33	93
1170	**Gunnerales**	62	143	69	155	63	141	63	144	55	116	55	144
1171*	**Buxales**	99	124	96	138	99	122	99	123	99	112	99	124
1173	**Trochodendrales**	3	29	4	30	3	28	3	28	2	24	2	29
1175*	**Proteales**	108	151	110	173	108	148	107	150	108	125	107	151
1178	**Sabiales**	48	126	49	140	45	125	48	127	41	110	41	127
1179	**Ranunculales**	101	137	103	152	101	136	101	138	96	123	96	138

1182*	Stem-Menispermaceae	83	103	81	118	83	103	83	104	82	97	82	104
1193*	Monocots	144	179	159	203	141	176	141	181	123	135	123	181
1200	**Poales**	76	106	74	114	72	104	78	108	64	94	64	108
1208	**Zingiberales**	75	89	71	93	75	88	75	89	74	84	74	89
1209*	Stem-*Musaceae*	74	84	68	87	74	84	74	84	74	81	74	84
1210	**Commelinales**	62	99	62	106	55	98	58	100	52	90	52	100
1211*	**Arecales**	82	95	77	99	82	94	82	94	82	91	82	95
1212	**Asparagales**	94	123	100	136	93	121	93	124	85	105	85	124
1221*	Orchidaceae	18	21	13	76	18	21	18	22	18	21	18	22
1222*	**Liliales**	66	115	75	129	65	113	65	115	58	98	58	115
1224*	Dioscoreales-Pandanales	91	131	105	152	90	128	90	131	89	108	89	131
1225	**Pandanales**	45	99	48	113	44	97	45	99	38	86	38	99
1226*	**Alismatales**	105	143	125	172	105	140	104	143	98	116	98	95
1228*	*Alisma-Potamogeton*	66	94	74	128	66	92	66	95	66	82	66	95
1232*	Araceae	76	112	88	151	76	111	76	113	76	98	76	113
1235	Magnoliidae	141	190	155	212	141	185	141	190	128	140	128	190
1237*	**Laurales**	107	134	107	153	107	132	107	133	107	121	107	134
1248*	**Magnoliales**	110	129	108	142	110	128	110	129	109	120	109	129
1256*	Stem-Canellales	128	179	143	203	127	174	128	179	117	134	117	179
1257	**Piperales**	106	152	115	171	105	148	106	152	95	120	95	152
1260*	Stem-*Saururus*	44	63	43	84	44	63	44	63	44	59	44	63
1264	**Canellales**	67	135	70	154	67	131	67	134	61	112	61	135
1268*	**Chloranthales**	92	113	88	126	92	113	92	113	92	107	92	113
1271	**Austrobaileyales**	119	167	119	180	119	165	119	167	115	135	115	167
1272*	Stem-Schisandraceae	107	132	105	148	107	132	107	132	107	121	107	132
1273*	Stem-*Illicium*	85	103	80	112	85	104	85	103	85	101	85	104
1275	**Nymphaeales**	128	189	129	198	128	185	129	188	119	140	119	189
1276*	Stem-*Cabombaceae*	110	128	107	138	110	128	110	128	109	119	109	128
1278*	Nymphaceae	93	116	91	126	93	116	93	116	94	111	93	116
1279*	Acrogymnosperms	308	357	307	342	308	355	307	351	307	345	307	357
1280*	Conifers	274	327	254	318	271	325	264	322	265	321	265	327
1282*	Gnetales	116	160	113	184	115	161	116	166	115	151	115	166
1284*	*Ginkgo-Cycas*	264	317	263	310	264	318	263	313	263	311	263	317
1286*	Ferns	383	413	382	411	383	409	383	415	383	415	383	413
1287*	Lycophytes	390	430	395	452	391	432	389	443	390	444	389	443

Nodes are numbered as in Figure S2. The 52 calibrated nodes are represented by (*) and nodes in bold characters represent major angiosperm orders. Posterior times are the 95% HPD CI, estimated using the HKY85+Γ_5 substitution model, IR rate model, with the 83 genes subdivided into three partitions: (1) 1st and 2nd codon positions for plastid genes; (2) 1st and 2nd codon positions for mitochondrial genes; and (3) nuclear RNA genes. Composite: 95% high posterior density credibility interval (HPD CI) is a composite of the 95% HPD credibility intervals across all calibration strategies, except calibration strategy B (SB).

Table S5. The 95% HPD limits of posterior divergence times, in millions of years before the present, for selected nodes in the vascular plant tree under different partition strategies, AR rate model, birth-death parameters and excluding lycophytes and ferns.

node	Clade	SA-IR-1P		SA-IR-MP		SA-AR-3P		SA-IR-3P-EP		SA-IR-3P-BD1		SA-IR-3P-BD2	
645*	Tracheophytes	437	457	443	458	437	456	N/A	N/A	444	460	443	458
646*	Euphylophytes	418	452	430	454	422	451	N/A	N/A	425	454	433	454
647*	Spermatophytes	341	375	355	389	344	371	355	391	354	393	354	418
648*	Angiosperms	223	263	213	261	230	265	231	271	214	263	217	257
651*	Mesangiosperms	184	222	177	211	189	216	186	223	178	214	180	211
655*	Eudicots	164	196	159	187	166	188	166	198	160	190	161	188
661	Superrosids	143	167	140	160	138	154	145	170	139	163	140	160
662	Rosids	141	165	138	158	137	153	143	169	138	161	139	158
668	**Malpighiales**	118	138	114	131	109	121	119	139	115	134	116	132
701*	Stem-*Malphighia*	45	53	44	52	44	50	44	52	44	51	44	52
753*	Stem-*Salix* plus *Populus*	49	58	48	55	49	55	48	55	48	55	48	55
776*	Stem-Clusiaceae	91	115	89	107	85	93	91	112	89	108	89	107
796	**Oxidales**	104	140	105	135	116	130	106	142	102	135	101	134
809	**Celastrales**	95	131	90	123	100	116	95	130	91	124	91	124
825*	**Fagales**	86	111	86	109	94	115	86	112	86	109	86	108
830*	Fagaceae	48	67	48	64	51	80	48	65	48	64	48	65
832	**Cucurbitales**	67	113	78	112	85	110	81	118	78	113	77	112
837	**Rosales**	91	125	91	120	100	118	96	128	92	123	93	121
847	**Fabales**	87	129	89	124	91	116	93	131	89	125	89	124
848*	Stem-*Polygalaceae*	79	123	80	117	86	112	84	124	80	117	81	117
855	**Zygophyllales**	48	124	50	111	66	111	53	117	49	112	50	112
862	**Brassicales**	76	110	82	107	78	100	85	112	82	108	82	107
869	**Malvales**	70	108	74	103	77	98	76	107	73	103	73	103
874	**Huertales**	29	103	31	98	54	101	29	100	30	97	29	97
875*	**Sapindales**	65	99	68	98	77	100	67	100	66	97	67	97
878*	Stem-*Ailanthus* plus *Citrus*	52	63	52	62	52	64	52	62	52	62	52	62
881	**Crossosomatales**	83	134	84	131	111	130	95	140	93	134	91	133
887*	**Myrtales**	84	117	86	116	84	104	87	123	85	116	85	113
895	**Geraniales**	85	134	12	58	110	130	10	80	22	41	14	61
897*	**Vitales**	62	84	63	81	63	100	62	81	62	81	62	81
898	**Saxifragales**	101	137	96	126	108	134	99	132	97	127	97	127
901*	Saxifragales core	88	119	87	114	94	122	89	119	88	114	87	114
914*	Stem-Hamamelidaceae	81	101	81	94	89	118	81	98	81	97	81	97
921	Superasterids	141	166	138	159	138	155	143	169	138	161	139	158
924	Asterids	127	151	123	144	126	141	128	153	124	146	124	144
933	**Dispacales**	84	111	76	100	95	111	79	105	75	100	76	100
952*	Stem-Dipelta	34	42	34	40	34	41	33	40	34	40	34	41
962	**Paracryphiales**	38	106	45	97	84	110	51	104	49	100	50	100
964	**Apiales**	89	117	85	110	101	115	87	114	84	110	84	108
983*	Araliaceae core	37	44	37	43	38	59	37	42	37	42	37	42
993	**Bruniales**	77	121	74	111	99	117	79	116	77	112	77	110
996	**Escalloniales**	74	120	79	112	103	118	81	117	78	113	77	111
1002	**Asterales**	97	122	89	109	92	108	91	114	89	109	89	109
1009*	Stem-Asteraceae minus *Barnadesia*	47	77	46	68	53	72	47	71	46	68	46	67
1041	**Aquifoliales**	91	129	90	121	109	126	94	129	92	123	91	121
1047*	Stem-*Aquifoliaceae*	61	83	62	81	66	105	62	83	62	81	62	81
1054	**Lamiales**	82	109	76	100	86	102	81	107	78	103	77	100
1075*	**Solanales**	78	112	77	104	93	109	80	111	79	108	78	105
1083	**Gentaniales**	69	103	65	96	79	99	69	102	67	99	66	96
1090	**Boraginales**	37	95	48	90	77	98	51	96	50	94	49	92
1093	**Garryales**	45	110	55	107	91	118	62	116	59	112	59	111
1094	**Ericales**	103	130	100	123	101	118	102	128	100	123	99	122
1098*	Ericales core	85	104	85	99	86	101	85	100	85	98	85	98
1115*	**Cornales**	86	125	85	114	107	133	85	119	85	118	85	117
1120	**Caryophyllales**	116	143	112	134	106	122	114	139	110	134	111	134
1162	**Berberidopsidales**	18	105	18	94	64	138	18	112	18	111	18	109
1163	**Santanales**	93	145	103	145	117	142	107	151	103	144	103	143
1168	**Dilleniales**	36	109	36	85	37	88	42	95	38	92	39	92
1170	**Gunnerales**	66	162	72	150	104	147	66	151	63	146	61	145
1171*	**Buxales**	99	131	99	124	102	144	99	127	99	125	99	124
1173	**Trochodendrales**	5	55	4	30	18	73	4	31	3	29	3	28
1175*	**Proteales**	108	156	108	148	150	176	108	159	108	152	108	151
1178	**Sabiales**	37	133	47	125	91	154	48	134	48	129	46	129
1179	**Ranunculales**	105	157	102	141	107	156	103	142	101	138	101	139
1182*	Stem-Menispermaceae	83	108	83	106	83	118	83	106	82	103	83	103
1193*	Monocots	141	184	146	179	154	182	150	193	146	182	149	182
1200	**Poales**	76	108	74	105	84	101	79	113	82	108	69	104
1208	**Zingiberales**	75	90	75	87	75	86	75	91	75	90	75	89

1209*	Stem-*Musaceae*	74	86	74	84	74	83	74	85	74	85	74	84
1210	**Commelinales**	47	96	52	96	80	99	57	103	62	99	61	99
1211*	**Arecales**	82	96	82	95	82	90	82	96	82	95	82	95
1212	**Asparagales**	90	124	92	122	105	120	99	130	95	124	94	123
1221*	Orchidaceae	18	23	18	21	18	21	18	22	18	22	18	21
1222*	**Liliales**	51	120	66	114	88	115	69	122	67	117	66	114
1224*	Dioscoreales-Pandanales	87	132	89	126	109	130	92	139	90	132	92	131
1225	**Pandanales**	33	101	45	98	72	109	47	104	44	99	45	99
1226*	**Alismatales**	101	140	105	141	121	147	109	154	106	145	106	144
1228*	*Alisma-Potamogeton*	66	91	66	95	66	83	67	105	67	96	67	96
1232*	Araceae	76	108	76	114	91	130	76	122	76	114	76	115
1235	Magnoliidae	135	188	140	183	179	207	147	201	144	192	144	195
1237*	**Laurales**	108	136	108	133	123	162	108	138	108	135	108	134
1248*	**Magnoliales**	110	134	110	128	117	157	110	133	110	130	110	129
1256*	Stem-Canellales	118	169	127	170	169	201	134	190	130	180	130	186
1257	**Piperales**	96	142	108	148	113	169	111	160	108	154	108	156
1260*	Stem-*Saururus*	44	61	44	62	44	72	44	65	44	63	44	64
1264	**Canellales**	70	137	68	131	124	179	70	140	68	136	68	136
1268*	**Chloranthales**	92	121	92	114	93	144	92	114	92	114	92	113
1271	**Austrobaileyales**	115	175	119	168	132	192	120	174	119	170	119	169
1272*	Stem-Schisandraceae	107	135	107	133	109	153	107	135	107	134	107	133
1273*	Stem-*Illicium*	85	107	85	103	85	114	85	103	85	104	85	103
1275	**Nymphaeales**	124	197	125	185	145	208	132	202	130	193	129	196
1276*	Stem-*Cabombaceae*	110	135	110	128	110	137	110	130	110	129	110	129
1278*	Nymphaceae	93	116	93	116	94	124	93	118	93	116	93	116
1279*	Acrogymnosperms	307	346	307	345	306	326	310	362	307	343	308	355
1280*	Conifers	217	314	265	323	262	309	278	328	268	320	267	328
1282*	Gnetales	116	169	117	164	116	170	116	161	117	156	115	153
1284*	*Ginkgo-Cycas*	263	313	263	305	273	315	264	319	263	312	263	311
1286*	Ferns	383	411	383	410	383	405	N/A	N/A	383	398	384	413
1287*	Lycophytes	391	423	390	445	392	441	N/A	N/A	392	447	390	428

Nodes are numbered as in Figure S2. The 52 calibrated nodes are represented by (*) and nodes in bold characters represent major angiosperm orders. Posterior times are the 95% HPD CI, estimated using the HKY85+Γ_5 substitution model, and calibration strategy A (SA). 1P, the tree partitions analysed as a single partition; MP, mixed partitions of plastid proteins, mitochondrial proteins and nuclear RNA genes; IR, independent rates model; AR autocorrelated rates model and birth-death parameters adjusted to generate a tree with long internal branches and short tip branches (BD1) and large node ages with nodes close to the root (BD2). N/A: not applicable.

NOTES S1 – Justification of fossil calibrations

Node 645 | CG Tracheophyta | MRCA: Lycophyta-Euphyllophyta | 422 Ma – 451 Ma.
Fossil taxon and specimen. Clarke at al. [1] based their calibration of this node on *Zosterophyllum* sp. [US384-8137; University of Saskatchewan Collec but more accurate timescale but more accurate timescale tions, Canada] from Bathurst Island [2].
Phylogenetic justification. Following Clarke et al. (2011), the *Zosterophyllum* sp. from Bathurst Island (Kotyk et al. 2002) is unequivocally zostrophyll given its possession of reniform sporangia, sporangia that dehisce along their distal margins, and laterally inserted sporangia. All *Zosterophyllum* species are total group Lycopsida [3].
Minimum age. 422 Ma.
Maximum age. 451 Ma.
Age justification. *Zosterophyllum* sp. on Bathurst Island [2] co-occurs with conodont *Ozarkodina douroensis*, which is restricted to the Ludlow (as O. n. sp. B in [4-7]. Thus, a minimum age interpretation can be derived from the top of the Ludlow, dated to 423.0 Ma ± 1.0 Myr, thus 422.0 Ma. The soft maximum constraint, following Clarke et al. [1], is based on the oldest occurrences of trilete spores, known from the Qusaiba-1 core from the Quasim Formation of northern Saudi Arabia [8]. Thus, we establish our soft maximum for Tracheophyta at 451 Ma. The very oldest records precede the earliest occurrences of the *Acanthochitina barbata*, opening the possibility that they occur in the preceding *Tanuchitina fistulosa* biozone, though *T. fistulosa* does not occur. The oldest stratigraphic records within the core co-occur with the chitinozoan *Armoricochitina nigerica*, known to extend into the Caradoc, to within the biozone characterized by *Fungochitina spinifera* (= *Fungochitina fungiformis*) [9]. The base of the *F. spinifera* zone falls within the *Dicranograptus clingani* Biozone (*Dicellograptus morrisi* subzone) [10], the base of which is estimated at 451 Ma [11].

Node 646 | CG Euphyllophytes | MRCA: Ferns-Spermatophyta | 385.571 Ma – 451 Ma.
Fossil taxon and specimen. *Rellimia thomsonii* from the Panther Mountain Formation of New York [12] [335.34; Paleobotanical Collection of the State University of New York at Bingham].
Phylogenetic justification. Magallón et al. [13] identified *Ibyka amphikoma* [14] as the oldest record of the pteridophyte lineage based on phylogenetic analyses undertaken by Kenrick and Crane [3].
Minimum age. 384.71 Ma.
Maximum age. 451 Ma.
Age justification. Clarke et al. [1] proposed *Rellimia thomsonii*, an aneurophytalean progymnosperm from the Panther Mountain Formation of New York [12], as the oldest record of crown Euphyllophyta. The Panther Mountain Formation is equivalent to the Ludlowville and Skaneateles formations [1], which occur below the Moscow Formation of New York [15], making *Rellimia thomsonii* older than *Ibyka amphikoma* [1]. The Ludlowville-Moscow formation boundary falls deep within the Lower *varcus* zone [16] and, therefore, below the *rhenanus-ansatus* biozonal boundary [17], at the very least, which has been dated to 386.25 Ma ± 0.679 Myr, yielding a minimum constraint of 385.571 Ma. The soft maximum constraint, following Clarke et al. [1], is based on the oldest occurrences of trilete spores, known from the Qusaiba-1 core from the Quasim Formation of northern Saudi Arabia [8]. The very oldest records precede the earliest occurrences of the *Acanthochitina barbata*, opening the possibility that they occur in the preceding *Tanuchitina fistulosa* biozone, though *T. fistulosa* does not occur. The oldest stratigraphic records within the core co-occur with the chitinozoan *Armoricochitina nigerica*, known to extend into the Caradoc, to within the biozone characterized by *Fungochitina spinifera* (= *Fungochitina fungiformis*) [9]. The base of the *F. spinifera* zone falls within the *Dicranograptus clingani* Biozone (*Dicellograptus morrisi* Subzone) [10]et al. ,2008) [11], the base of which is estimated at 451 Ma(Cooper and Saddler 2012). Thus, we establish our soft maximum for Tracheophyta at 451 Ma.
Discussion. Magallón et al. [13] established a minimum age constraint using *Ibyka amphikoma,* based on the Givetian-Frasnian boundary, for which they provided a date of 385 Ma, though this has since been revised to 382.7 Ma ± 1 Myr [17]. *Ibyka amphikoma* was recovered from the Manorkill Shale Member, which is a lateral equivalent of the Windom Member, within the Moscow Formation of New York [18, 19], which falls fully within the *ansatus* conodont Biozone [20, 21] the top of which is dated to 385.41 Ma ± 0.7 Myr [17], thus, yielding a minimum age constraint of 384.71 Ma, younger than the minimum age of *Rellimia thomsonii*.

Node 647 | CG Spermatophytes | MRCA: *Ginkgo-Austrobuxus* | 308.14 Ma – 365.629 Ma.
Fossil taxon and specimen. *Cordaites iowensis* [UIC 12,233: University of Illinois at Chicago; OUPH 9616-9742: Ohio University Paleobotanical Herbarium, Department of Botany, Ohio University, Athens, Ohio] from the Laddsdale Coals (Cherokee Group, Desmoinesian) near What Cheer, Iowa [22].

Phylogenetic justification. Clarke *et al*. [1] identify cordaitean coniferophytes as the oldest records of the crown group of the spermatophyte clade. The oldest whole plant reconstruction is *Cordaites iowensis* from the Laddsdale Coals (Cherokee Group, Desmoinesian) near What Cheer, Iowa [22].
Minimum age. 308.14 Ma.
Maximum age. 365.629 Ma.
Age justification. Janousek and Pope [23] argue that the Laddsdale Coal is equivalent to the Bluejacket Coal of Oklahoma, which occurs as part of the Bluejacket Sandstone Member, underlying the Inola Limestone, part of the Inola Cyclothem of the Krebs subgroup of the Cherokee Group, characterized by the occurrence of the conodonts *Idiognathodus amplificus, Idiognathodus podolskensis and Neognathodus asymmetricus* [24]. The Inola cyclothem falls fully within the *Idiognathodus amplificus/ Idiognathodus obliquus* biozone [25]. This is indicative of the *Neognathodus medexultimus-Streptognathodus concinnus* (Pc10) biozone, certainly older than the *Neognathodus roundyi – Streptognathodus cancellosus* (Pc11) biozone [25, 26]. The base of Pc10 is bracketed by an older age constraint of 312.01 Ma ± 0.37 Myr and the base of Pc11 is bracketed by a younger age constraint of 308.5 Ma ± 0.36 Myr in the Composite Standard of Davydov et al. [26], yielding a minimum constraint of 308.14 Ma.

The soft maximum constraint follows Clarke et al. [1] who based theirs on the first records of seeds in the form of preovules that satisfy the criteria of the seed habit, which occur in the Upper Fammenian (Late Devonian) VCo Spore Biozone [27], a well documented example of which being *Elkinsia polymorpha* [28]; *E. polymorpha* has been recovered from the Hampshire Formation, West Virginia, from which the palynomorphs *Grandispora cornuta, Retispora macroreticulata, Retusotriletes phillipsii* and *Rugospora radiata* have been reported [29], which substantiate assignment to the VCo Biozone [30]. The VCo biozone is not directly dated but its base falls within the *Palmatolepis trachytera* conodont biozone [31], the base of which is dated to 364.19 Ma ± 1.439 Myr [17], yielding a soft maximum constraint on the divergence of crown Spermatophyta at 365.629 Ma.

Node 648 | CG Angiosperms | MRCA: *Amborella-Austrobuxus* | 125.9 Ma – 247.3 Ma.
Fossil taxon and specimen. Tricolpate pollen grain [BRN 126] from the Cowleaze Chine Member of the Vectis Formation of the Isle of Wight [32].
Phylogenetic justification. Following Clarke at al. [1], our minimum age constraint is based on the earliest occurrences Fischer's rule tricolpate pollen, and knowledge of the distribution of tricoplate pollen across the phylogeny of angiosperms [33].
Minimum age. 125.9 Ma.
Maximum age. 247.3 Ma.
Age justification. Following Clarke at al. [1], the Cowleaze Chine Member of the Vectis Formation of the Isle of Wight (Hughes & McDougall, 1990) occurs within the M1n polarity chron at the top of the Barremian, dated as 126.3 Ma ± 0.4 Myr [34]. The soft maximum age constraint is based on sediments devoid of angiosperm-like pollen below their first report in the Middle Triassic, thus, the base of the Anisian, dated to 247.1 Ma ± 0.2 Myr [35], thus, 247.3 Ma.
Discussion. The recently described *Euanthus panii* [36], *Juraherba bodae* [37] and *Yuhania dahugouensis* [38] from the Jiulongshan Formation were considered but not assigned. At the current stage, the age of the formation appears to be still not fully settled despite most experts agree on a middle Jurassic age (see [37, 38]), whereas the assignment to extant lineages also required further investigation using phylogenetic approaches to confirm the proposed relationships of *Juraherba* to Hydatellaceae - which are the sister to the remaining Nymphaeales lineage (node 1276) and *Yuhania* to monocots.

Node 651 | CG Mesangiosperms | MRCA: *Chloranthus-Austrobuxus* | 125.9 Ma – 247.3 Ma.
Fossil taxon and specimen. Tricolpate pollen grain [BRN 126] from the Cowleaze Chine Member of the Vectis Formation of the Isle of Wight. [32].
Phylogenetic justification. Following Clarke at al. [1], our minimum age constraint is based on the earliest occurrences Fischer's rule tricolpate pollen, and knowledge of the distribution of tricoplate pollen across the phylogeny of angiosperms [33].
Minimum age. 125.9 Ma.
Maximum age. 247.3 Ma.
Age justification. Following Clarke at al. [1], the Cowleaze Chine Member of the Vectis Formation of the Isle of Wight [32] occurs within the M1n polarity chron at the top of the Barremian, dated as 126.3 Ma ± 0.4 Myr [34]. The soft maximum age constraint is based on sediments devoid of angiosperm-like pollen below their first report in the Middle Triassic, thus, the base of the Anisian, dated to 247.1 Ma ± 0.2 Myr [35], thus, 247.3 Ma.

Node 655 | CG Eudicots | MRCA: *Dicentra-Austrobuxus* | 119.6 Ma.
Fossil taxon and specimen. *Hyrcantha decussata* [NJU-DES02001: Geological Institute, Chinese Academy of Sciences, Beijing], from the lower part of the Yixian Formation, Jehol Group, Liaoning Province, China [39].
Phylogenetic justification. Similar to *Leefrutcus* from the Yixian formation of the Lower Cretaceous of China, *Hycantha* is considered to be a stem group representative of the Ranunculales [40].
Minimum age. 119.6 Ma.
Age justification. The main fossil bearing beds have been dated and may be as old as 129.2 Ma [41], however, in the absence of knowledge of the position of the fossils within the stratigraphy, relative to the sources of the absolute dates, a minimum age constraint can be derived from the Jiufontang Formation which overlies it. ^{40}Ar/^{39}Ar dating of a number of samples from the Jiufontang Formation has yielded an age of 120.3 ± 0.7 Ma for the volcanic tuffs [42], establishing a minimum constraint of 119.6 Ma.

Node 701 | SG Malphighia | MRCA: *Dicella-Malphighia* | 44.83 Ma.
Fossil taxon and specimen. *Perisyncolporites pokornyi* [UFP65: Paleobotanical Collection of the Florida Museum of Natural History, University of Florida: Gainesville, Florida, USA] from central Colombia [43].
Phylogenetic justification. Assigned to Malpighiales [43, 44]. Based on morphological similarities, the fossils are considered as reliable assigned to the stem of the stigmaphyllloid clade [45].
Minimum age. 44.83 Ma.
Age justification. The minimum age of *Perisyncolporites pokornyi* is best constrained in sections in central Colombia which Jaramillo and Dilcher [43] integrated into a graphic correlation composite standard and, on this basis, were able to establish its first occurrence as within the Lower Eocene. This composite standard has been refined and calibrated to absolute time by Jaramillo et al. [46] who provide a date of 44.83 Ma for the first appearance of *Perisyncolporites pokornyi.*

Node 753 | SG *Salix* plus *Populus* | MRCA: *Idesia-Salix* | 48.57 Ma.
Fossil taxon and specimen. *Pseudosalix handleyi* [UMNH PB-1: Utah Museum of Natural History, Salt Lake City, USA] from lacustrine shales of the Parachute Creek Member of the Green River Formation in the vicinity of Bonanza, Utah, USA [47].
Phylogenetic justification. Our node assignment follows the currently accepted interpretation of the fossil record of Salicaceae [48].
Minimum age. 48.57 Ma.
Age justification. The Parachute Creek Member reaches into C22n magnetozone [49], the minimum age of which can be established from the base of the succeeding C21r, dated to 48.57 Ma in the combined age model of Vandenberghe et al. [50].

Node 776 | SG Clusiaceae | MRCA: Clusia-Hypericum | 85.8 Ma.
Fossil taxon and specimen. *Paleoclusia chevalieri* [CUPC 1192: L. H. Bailey Hortorium Paleobotanical Collection, Cornell University, Ithaca, NY, USA] from the Old Crossman locality, New Jersey, USA [51].
Phylogenetic justification. The phylogenetic interpretation follows Ruhfels [52].
Minimum age. 85.8 Ma.
Age justification. Clarke et al. [1] argued that a minimum constraint on the age of this deposit could be established from Santonian-Campanian Boundary, however, Massoni et al. [53] argue that a tighter correlation can be established with better rocks attributable to the CC13-14 Nannofossil zones in South Carolina, indicating a minimum age of 86.3 Ma ± 0.5 Myr, thus, 85.8 Ma.

Node 825 | CG Fagales | MRCA: *Nothofagus-Fagus* | 85.8 Ma.
Fossil taxon and specimen. *Nothofagidites senectus* [GSV 61898: Mines Department of Victoria, Melbourne, Australia] from the Bass and Gippsland Basins, Australia [54]
Phylogenetic justification. These microfossils are widely considered to be Nothofagaceae which in turn are the sister to the remaining Fagales clade [55].
Minimum age. 85.8 Ma.
Age justification. The first appearance of *Nothofagidites senectus* defines the base of the *Nothofagidites senectus* Palynozone, which coincides with the Coniacian-Santonian Boundary , which is dated to 86.3 Ma ± 0.5 Myr [34], affording a minimum age constraint of 85.8 Ma.

Node 830 | CG Fagaceae | *Fagus-Quercus* | 47.6 Ma.
Fossil taxon and specimen. *Fagus langevinii* [UWBM 97583: Burke Museum, Seattle, Washington, USA] preserved as impressions and carbonaceous films in a siliceous shale in an unnamed formation within the Kamloops Group at the McAbee Locality near the town of Cache Creek, British Columbia [57].
Phylogenetic justification. This fossil is accepted as a stem group representative of the genus *Fagus* [58].

Minimum age. 47.6 Ma.
Age justification. Ashes within the 30 metre sequence of fossiliferous shales have yielded dates based on K-Ar geochronology, ranging between 52 Ma ± 2 Myr and 49 Ma ± 2 Myr [59]. Correlation based on facies and pollen biostratigraphy establish an Ypresian age for the fossil assemblage , compatible with the aged from the intercalated ashes. Therefore, a minimum age constraint can be established on the Ypresian-Lutetian Boundary which has been dated to 47.8 Ma ± 0.2 Myr based on the combined age model of Vandenberghe et al. [50], thus, 47.6 Ma.

Node 848 | SG Polygalaceae | MRCA: *Polygala-Medicago* | 61.6 Ma.
Fossil taxon and specimen. *Paleosecuridaca curisii* [PP34562: Field Museum, Chicago, IL, USA], described by from the Sentinel Butte Formation at the Almont site in Morton County, central North Dakota [62].
Phylogenetic justification. The phylogenetic relationships were clarified by Pigg [61].
Minimum age. 61.6 Ma.
Age justification. The age of the Sentinel Butte Formation has been assigned to the Tiffanian 3 based on mollusk and mammal-based biostratigraphy, but the most concrete age evidence is based on palynostratigraphy, assigned the Almont Site sediments of the Sentinel Butte Formation to Pollen Zone 5 of the late Palaeocene [63]. In the absence of further constraint we establish a minimum age for *Paleosecuridaca curisii* based on the Palaeocene-Eocene boundary, dated to 61.6 Ma in the combined age model of Vandenberghe et al. [50].

Node 875 | CG Sapindales | MRCA: *Citrus-Nitraria* | 59.24 Ma.
Fossil taxon and specimen. *Dipteronia brownii* [UF 15740E-23086: Florida Museum of Natural History, Gainesville FL, USA] from the Paleocene Fort Union Formation at Hell's Half Acre, Wyoming [64].
Phylogenetic justification. This fossil is assigned to the extant genus *Dipteronia* which belongs to the subfamily Hippocantanoides of the family Sapindaceae. The extant genus is considered a Tertiary relict having two extent species endemic to China [65, 66]. Being a possible stem group representative of the extant genus nested in the Sapindales provided the framework for this assignment.
Minimum age. 59.24 Ma.
Age justification. *Dipteronia brownii* occurs within the P4 Pollen Zone in the type section of Nichols and Ott [67], which falls fully within Magnetic Anomaly Zone C26r [68], the end of which is dated to 59.24 Ma in the combined age model of Vandenberghe et al. [50].

Node 878 | SG Ailanthus plus *Citrus* | MRCA: *Ailanthus-Swietenia* | 51.83 Ma.
Fossil taxon and specimen. *Ailanthus confucii* (senior synonym of *Ailanthus lesquereuxi*) [DMNH 7879: Denver Museum of Natural History, Denver CO, USA] from the Fossil Butte fish quarries of the Green River Formation near Kemerrer, Wyoming [69].
Phylogenetic justification. This fossil is accepted to belong to the extant genus *Ailanthus* and can be therefore assigned to this clade [69].
Minimum age. 51.83 Ma.
Age justification. The Fossil Butte Member of the Green River Formation is bounded minimally by the C23r magnetozone [49], the minimum age of which can be established from the base of the succeeding C23n, dated to 51.83 Ma in the combined age model of Vandenberghe et al. [50].

Node 887 | CG Myrtales | MRCA: *Myrtus-Oenothera* | 83.3 Ma.
Fossil taxon and specimen. *Esqueiria futabensis* [PP45419: Field Museum, Chicago IL, USA] from two levels in the Futaba Group exposed in Fukushima Prefecture. northeastern Honshu, Japan [70].
Phylogenetic justification. The phylogenetic relationships have been established by several authors [71].
Minimum age. 83.3 Ma.
Age justification. One locality, considered Coniacian, occurs in the Asamigawa Member of the Ashizawa Formation, on a tributary of the Kitaba River in Kamikitaba, Hirono-machi. Unfortunately, no material evidence has been presented to substantiate this age assignment (Takahashi et al. [70], among others, merely cite the presence of unspecified Coniacian ammonites). The second locality is in the middle part of the Tamayama Formation, on the Kohisa River, Kohisa, Ouhisa machi, northeast of lwaki City. The Asamigawa Formation is the lowermost formation in the Futaba Group, and is overlain by the Kasamatsu Formation, in turn overlain by the Tamayama Formation. The age of the Tamayama Formation is substantiated on the presence of *Inoceramus amakusensis* [70], which is restricted to the Santonian [72]. Thus, a minimum age constraint may be established on the Santonian-Campanian Boundary, dated as 83.6 Ma ± 0.3 Myr [34], thus, 83.3 Ma.

Node 897 | CG Vitales | MRCA: *Vitis-Leea* | 65.508 Ma.
Fossil taxon and specimen. *Indovitis chitaleyae* [UF19279-56220: Florida Museum of Natural History (UF) Gainesville, Florida, USA] preserved as fruits and seeds in chert from Deccan Intertrappean beds exposed in a

quarry near the village of Mahurzari, India about 14 km from Nagpur, along with young fruits with intact seeds and isolated mature seeds from a series of localities including Mohgaonkalan in Chhindwara District and Ambabagholi in Baitul District, both in Madhya Pradesh, and Shibla in Yeotmal District, Maharashtra [73].
Phylogenetic justification. The phylogenetic relationships of this fossil have been clarified previously [73].
Minimum age. 65.508 Ma
Age justification. A minimum constraint on the age of *Indovitis chitaleyae* can be established on minimum age of Deccan volcanism, which has been constrained to 65.535 Ma ± 0.027 Myr [74], thus, 65.508 Ma.

Node 901 | CG Saxifragales core | MRCA: *Haloragis-Itea* | 85.8 Ma.
Fossil taxon and specimen. *Divisestylus brevistamineus* [CUPC 1340: L. H. Bailey Hortorium Paleobotanical Collection, Cornell University, Ithaca NY, USA], flowers described from the Old Crossman locality, New Jersey, USA [75].
Phylogenetic justification. The phylogenetic relationships of this fossil have been clarified previously [75].
Minimum age. 85.8 Ma
Age justification. Clarke et al. [1] argued that a minimum constraint on the age of this deposit could be established from Santonian-Campanian Boundary, however, Massoni et al. [53] argue that a tighter correlation can be established with better rocks attributable to the CC13-14 Nannofossil zones in South Carolina, indicating a minimum age of 86.3 Ma ± 0.5 Myr, thus, 85.8 Ma.

Node 914 | SG Hamamelidaceae | MRCA: *Daphniphyllum-Rhodoleia* | 82.00 Ma.
Fossil taxon and specimen. *Androdecidua endressii* [PP45947: Field Museum, Chicago IL, USA] from the Buffalo Creek Member of the Gaillard Formation in the south pit (Allon Quarry) of the Atlanta Sand and Supply Company in Gaillard, Georgia, ca. 9.5 km southeast of Roberta in Crawford County [76].
Phylogenetic justification. The phylogenetic relationships were carefully discussed by the authors introducing the taxon [76].
Minimum age. 82.00 Ma
Age justification. Christopher correlated this deposit to nannofossil zone CC17 based on its palynoflora, though the evidence on which this is based was not presented. CC17 ranges in age from Late Santonian to Early Campanian and the CC17-18 boundary falls within the *Scaphites hippocrepis* II ammonoid biozone, dated to 82.00 Ma [34].

Node 952 | SG Dipelta | *Dipelta-Kolkwitzia* | 33.71 Ma.
Fossil taxon and specimen. *Diplodipelta reniptera* [UM 33621: University of Michigan, Ann Arbor MI, USA] from the Florissant Formation, Mormon Cr, Ruby, CO, USA [78].
Phylogenetic justification. Based on the distribution of morphological characters in the phylogeny of Caprifoliaceae, this fossil is considered to be sister to the genus *Dipelta* which provide in turn the arguments for this assignment [78, 79].
Minimum age. 33.71 Ma.
Age justification. The Florissant Formation has been correlated with the early Chron C13r, based on its reversed polarity and a $^{40}Ar/^{39}Ar$ date of 34.07 Ma ± 0.10 Myr from the upper part of the section [80, 81]. The minimum age for the Florssant flora can be established on the C13r-C13n boundary, which has been dated to 33.71 Ma, based on the combined age model in Vandenberge et al. [50].

Node 983 | CG Araliaceae core | MRCA: *Cussonia-Tetralpasandra* | 37.3 Ma.
Fossil taxon and specimen. *Dendropanax eocenensis* [W1107': Indiana University paleobotanical collection] from the Claiborne Formation at Warman Clay Pit (2 miles west of Como), Tennessee, USA.
Phylogenetic justification. Phylogenetic relationships were discussed by Martinez-Millan [82].
Minimum age. 37.3 Ma.
Age justification. The Claiborne Formation is commonly attributed a middle Eocene age, but material evidence is rarely presented in support this. Taylor [83] provides a summary of the palynostratigraphic evidence, citing Elsik as supporting a middle Eocene age based on juglandaceous palynomorphs including *Plicatopollis, Platycarya, Platycaryapollenites, Carya* and *Casuarinidites*. Their presence may be indicative of a late Eocene age, though they occur in low frequency and, thus, Taylor [83] argues instead for a Middle Eocene age based on the presence of *Nuxpollenites terminalis* and *Amanoa* type palynomorphs in even the uppermost Claiborne Formation [84]. Thus, we establish a minimum age constraint on the age of *Solanites crassus* based on the Bartonian-Priabonian Boundary, dated to 37.8 Ma ± 0.5 Myr based on the combined age model of Vandenberghe et al. [50], thus, 37.3 Ma.

Node 1009 | SG Asteraceae minus Barnadesia | MRCA: *Barnadesia-Helianthus* | 41.5 Ma.
Fossil taxon and specimen. *Tubulifloridites antipodica* from onshore deposits taken from a paleochannel at Koingnaas, on the west coast of South Africa.
Phylogenetic justification. This fossil is accepted to belong to the Barnadesioides despite its actual placement in the subfamily is ambiguous but this is not a challenge to our analyses[82,85].
Minimum age. 41.5 Ma.
Age justification. These occurrences are, described to occur alongside the planktic forams *Globigerinatheka index* and *Turborotalia centralis* [85]. *Globigerinatheka index* is known to range from 42.9 - 34.3 Ma [86], but *Turborotalia centralis* is a junior synonym of *Turborotalia pomeroli*, which is known to range from 42.4-41.5 Ma [86]. Thus, the minimum age constraint on *Tubulifloridites antipodica* is 41.5 Ma. The newly described *Tubulifloridites lilliei* arguably slightly predates the other fossils of this palynological genus in its age[58].

Node 1047 | SG Aquifoliaceae | MRCA: *Ilex- Phyllonoma* | 61.6 Ma.
Fossil taxon and specimen. *Ilex hercynica* [MAI, Nr. 6004: Zentralsammlung Zentrales Geologisches Institut Berlin] described from Walkmühle in Gonna, Germany [87-89].
Phylogenetic justification. Phylogenetic relationships were discussed by Martinez-Millan [82].
Minimum age. 61.6 Ma
Age justification. Knobloch et al. [90] assert an early Palaeocene age range for *Ilex hercynica*, affording a minimum constraint of 61.6 Ma based on the Danian-Salandian Boundary and the combined age model of Vandenberghe et al. [50].

Node 1075 | CG Solanales | MRCA: *Solanum-Montinia* | 37.3 Ma.
Fossil taxon and specimen. *Solanites crassus* [USNM-39949: Smithsonian Institution National Museum of Natural History, Washington DC, USA] from Holly Springs Sand in the Claiborne Formation at Mill Creek, at a railroad cut north of Shandy, Hardeman County, Tennessee, USA [91].
Phylogenetic justification. The phylogenetic interpretation of the fossil record of Solanales have been explored recently including the fossil incorporated here .
Minimum age. 37.3 Ma.
Age justification. The Claiborne Formation is commonly attributed a middle Eocene age, but material evidence is rarely presented in support this. Taylor [83] provides a summary of the palynostratigraphic evidence, citing Elsik [84] as supporting a middle Eocene age based on juglandaceous palynomorphs including *Plicatopollis, Platycarya, Platycaryapollenites, Carya* and *Casuarinidites*. Their presence may be indicative of a late Eocene age, though they occur in low frequency and, thus, Taylor [83] argues instead for a Middle Eocene age based on the presence of *Nuxpollenites terminalis* and *Amanoa* type palynomorphs in even the uppermost Claiborne Formation [84]. Thus, we establish a minimum age constraint on the age of *Solanites crassus* based on the Bartonian-Priabonian Boundary, dated to 37.8 Ma ± 0.5 Myr based on the combined age model of Vandenberghe et al. [50], thus, 37.3 Ma,

Node 1098 | CG Ericales core | MRCA: *Arbutus-Camellia* | 85.8 Ma.
Fossil taxon and specimen. *Paleoenkianthus sayrevillensis* [CUPC 1100: L. H. Bailey Hortorium, Cornell University, Ithaca NY, USA] from the South Amboy Fire Clay of the Raritan Formation, of which outcrops are exposed in the Old Crossman Clay Pit in Sayreville, New Jersey.
Phylogenetic justification. The phylogenetic relationships of this fossil has been tested based on morphological evidence [93].
Minimum age. 85.8 Ma
Age justification. Clarke et al. [1] argued that a minimum constraint on the age of this deposit could be established from Santonian-Campanian Boundary, however, Massoni et al. [53] argue that a tighter correlation can be established with better rocks attributable to the CC13-14 Nannofossil zones in South Carolina, indicating a minimum age of 86.3 Ma ± 0.5 Myr, thus, 85.8 Ma.

Node 1115 | CG Cornales | MRCA: *Cornus-Petalyonyx* | 85.8 Ma.
Fossil taxon and specimen. *Tylerianthus crossmanensis* [CUPC 1047: L. H. Bailey Hortorium, Cornell University, Ithaca NY, USA] from the South Amboy Fire Clay of the Raritan Formation, of which outcrops are exposed in the Old Crossman Clay Pit in Sayreville, New Jersey [94].
Phylogenetic justification. The phylogenetic relationships of this fossil have been clarified [95, 96].
Minimum age. 85.8 Ma
Age justification. Clarke et al. [1] argued that a minimum constraint on the age of this deposit could be established from Santonian-Campanian Boundary, however, Massoni et al. [53] argue that a tighter correlation can be established with better rocks attributable to the CC13-14 Nannofossil zones in South Carolina, indicating a minimum age of 86.3 Ma ± 0.5 Myr, thus, 85.8 Ma.

Node 1171 | CG Buxales | MRCA: *Didymeles-Buxus* | 100.1 Ma.
Fossil taxon and specimen. *Spanomera marylandensis* [PP42978: Field Museum, Chicago IL, USA] described by Drinnan et al. from the Potomac Formation at West Brothers clay pit, east of Washington, DC, Prince Georges County, eastern Maryland (late Albian), assigned to Palynozone IIB.
Phylogenetic justification. Based on similarities in the inflorescences and the striate tricolpate pollen, this fossil is likely related to the extant Buxaceae [98].
Minimum age. 100.1 Ma
Age justification. In the absence of further constraint on where within Zone IIB *Spanomera marylandensis* was recovered, we take the upper age constraint on the age of this zone to establish a minimum age constraint. Massoni et al. [53] argued that deposits lower within IIB were middle Albian, but Hochuli et al. [99] have demonstrated that at least some of Zone IIB is Upper Albian. Thus, we establish our minimum age constraint on the Albian-Cenomanian Boundary, dated to 100.5 Ma 0.4 Myr [34].

Node 1175 | CG Proteales | *Nelumbo-Platanus* | 107.59 Ma.
Fossil taxon and specimen. *Aquia brookensis* [PP4295: Field Museum, Chicago IL, USA] described by Crane et al. [100] from the Potomac Formation at Bank, near Brooke, Virginia, USA.
Phylogenetic justification. Doyle [98] recognized *Aquia* (combination of *Sapindopsis variabilis, Platanocarpus brookensis, Aquia brookensis*) as a stem group member of the genus *Platanus* and the widespread Albian leaf fossil *Nelumbites* as related to the genus *Nelumbo*.
Minimum age. 107.59 Ma
Age justification. Palynological correlations place Brooke in lower Subzone II-B, for which Massoni et al. [53] provide a very detailed biostratigraphic justification for a middle Albian age, to which we refer readers. Thus, a minimum constraint for Proteales may be established on the middle-late Albian Boundary, which coincides with the base of the *Diploceras cristatum* Biozone, dated to 107.59 Ma [34].

Node 1182 | SG Menispermaceae | MRCA: *Menispermum-Ranunculus* | 83.41 Ma.
Fossil taxon and specimen. *Prototinomiscium testudinarum* and *P. vangerowii* from Klikov Formation, Czech Republic [89].
Phylogenetic justification. The phylogenetic relationships of the fossil were clarified in a study testing them as calibration points for divergence time estimates of Menispermacae [101].
Minimum age. 83.41 Ma
Age justification. An Upper Turonian-Santonian age was established for the Klikov Formation by Pacltova [102] and Knobloch [103]. Thus, a minimum constraint can be established on the Santonian-Campanian Boundary, coincident with the base of the *Scaphites Leei III* Zone, dated to 83.64 Ma ± 0.23 Myr [34], thus, 83.41 Ma.

Node 1193 | CG Monocots | MRCA: *Acorus-Saccharum* | 112.6 Ma.
Fossil taxon. The earliest records of *Liliacidites* occur at the Trent's Reach Locality of the Potomac Group, attributable to the Albian Zone I [99].
Phylogenetic justification. Doyle et al. [104] identified pollen referred to the genus *Liliacidites* (but not Similipollis) as represesentative of the monocot stem, making it the oldest secure record of the monocot total group (see [98]).
Minimum age. 112.6 Ma.
Age justification. In the absence of further stratigraphic constraint, these earliest records of *Liliacidites* can be constrained in age by the Aptian-Albian Boundary, dated to 113.0 ma ± 0.4 Myr, thus, 112.6 Ma.
Discussion. Doyle et al. [104] highlight that, despite decades of sampling of the Hauterivian and Barremian of England, no clear representatives of *Liliacidites* pollen have been recovered [105], perhaps implying that the earliest records from the Albian are a close approximation of their antiquity. Because of the position of monocots in our molecular tree we consider *Liliacidites* to be nested within monocots, and use it to calibrate the monocot crown node.

Node 1209 | SG Musaceae | MRCA: *Musa-Maranta* | 74.6 Ma.
Fossil taxon and specimen. *Spirematospermum chandlerae* has been described from isolated seeds and groups of seeds from the Neuse River locality, Black Creek Formation, southwest of Goldsboro, Wayne County, North Carolina, USA.
Phylogenetic justification. The phylogenetic relationships of this fossil have discussed in previous studies [106].
Minimum age. 74.6 Ma.

Age justification. Widely reported, but not evidenced as Late Cretaceous (Early Campanian) in age [107], the Black Creek Formation has been assigned to the *Exogyra ponderosa* Biozone which occurs beneath the *Didymoceras cheyennense* Tethyan ammonoid biozone [108], the base of which is dated to 74.6 Ma [34].

Node 1211 | CG Arecales | MRCA: *Elaeis-Chamaedorea* | 83.41 Ma.
Fossil taxon and specimen. *Sabalites carolinensis* [PAL 175717/P 38208: Smithsonian Museum of Natural History; Washington DC, USA] described from the Middendorf Arkose Member of Black Creek Formation near Langley, Aiken County, South Carolina [109].
Phylogenetic justification. The phylogenetic relationships of this fossil have discussed in Hertweck et al. [106] and Iles et al. .
Minimum age. 83.41 Ma
Age justification. Berry's view that the Middendorf was merely a distinct facies within the Black Creek Formation, rather than a stratigraphically distinct unit, has been rejected. Sohl and Owens [111] subdivided the Upper Cretaceous of Carolina coastal plain into three lithostratigraphic units, the Middendorf, Black Creek and Peedee Formations, raised the Black Creek to group status and subdivided this into three unconformity-bound formations, viz. in stratigraphic sequence, the Tar Heel, Bladen and Donoho Creek formations. Evidently, *Sabalites carolinensis* was recovered from what is now recognized as the Middendorf Formation, and a minimum age constraint can be established on the boundary between the Middendorf and Tar Heel Formations. The Middendorf is commonly considered Santonian in age, however, little material evidence has been presented in support of this, in part a consequent of the complex history of stratigraphic divisions at outcrop, in subsurface and offshore [112]. Habib and Miller [113] established an age 'not younger than Campanian' on the basis of dinoflagellate biostratigraphy, but following the stratigraphic scheme outlined Campbell and Grohn [112], the Middendorf is older that the Shepherd Grove Formation and, therefore, following the stratigraphy of Christopher and Prowell [108], must be no younger than Santonian. Thus, we may established a minimum age constraint on the *Sabalites carolinesis* based on the Santonian-Campanian Boundary, coincident with the base of the *Scaphites leei III* Zone, dated to 83.64 Ma ± 0.23 Myr [34], thus, 83.41 Ma.

Node 1221 | CG Orchidaceae | MRCA: *Onicidium-Phaelaenopsis* | 17.82 Ma.
Fossil taxon and specimen. *Meliorchis caribea* [MCZ-31141: Museum of Comparative Zoology (Harvard University), Cambridge MA, USA], recovered from a mine east of Santiago, Cordillera Septentrional, Dominican Republic [114].
Phylogenetic justification. This amber fossil is composed of a pollinium attached to the wing of a stingless bee showing some similarities to the extand genus *Ligeophila* [114]. Pollinium structure provide unequivocal evidence for the occurrence of Orchidaceae a this time.
Minimum age. 17.82 Ma.
Age justification. The age of Dominican Amber has been the subject of much speculation, but its dating has been best constrained on the basis of plantik forams, to late Early to early Middle Miocene [115] who list a series of biostratigraphically important species of planktic foraminifera, the stratigraphic ranges of which are not entirely compatible. However, the contemporaneous sediments contain including *Catapsydrax dissimillis*, the last appearance of which has been dated to 17.62 Ma [86], providing a minimum constraint on the age of *Meliorchis caribea*.

Node 1222 | CG Lillales | *Trillium-Lilium* | 18.7 Ma.
Fossil taxon and specimen. *Luzuriaga contortus* [L24916: Palynological Type Collection of th eNew Zealand Institute of Geological and Nuclear Sciences], based on leaf remains, from the Foulden Hill Diatomite, near Middlemarch, Otago, New Zealand [116].
Phylogenetic justification. The preserved structure show high similarity to structures observed in extend this of the genus *Luzuriaga* and therefore the fossil is assigned unequivocally to the Alstromeriaceae .
Minimum age. 18.7 Ma.
Age justification. The deposit has been well studied and its maximum age is constrained by radiometric dating to 23.3 Ma ± 0.2 Ma [117]. The deposit has been attributed to the Waitakian, but a minimum age constraint must also encompass evidence of early Otaian elements of the flora and fauna, including *Assamiapollenites incognitus* which extends into the Otaian *Proteacidites isopogiformis* Zone [118]. The Otaian-Altonian Boundary has been dated to 18.7 Ma [119, 120].

Node 1224 | CG Dioscoreales-Pandanales| MRCA: *Dioscorea-Croomia* | 85.8 Ma.
Fossil taxon and specimen. *Mabelia connatifila* [CUPC 1255: L. H. Bailey Hortorium Paleobotanical Collection,
Cornell University, Ithaca NY, USA] from the South Amboy Fire Clay Member of the Raritan Formation at the Old Crossman clay pit in Sayreville, New Jersey, USA [121].

Phylogenetic justification. The phylogenetic assignment is based on the phylogenetic hypothesis reconstructed by Gandolfo et al. [121].
Minimum age. 85.8 Ma.
Age justification. Clarke et al. [1] argued that a minimum constraint on the age of this deposit could be established from Santonian-Campanian Boundary, however, Massoni et al. [53] argue that a tighter correlation can be established with better rocks attributable to the CC13-14 Nannofossil zones in South Carolina, indicating a minimum age of 86.3 Ma ± 0.5 Myr, thus, 85.8 Ma.

Node 1226 | CG Alismatales | MRCA: *Orontium-Hydrocharis* | 96.24 Ma.
Fossil taxon and specimen. *Mayoa portugallica* [S136663: Swedish Museum of Natural History Palaeobotanical Collection], from the Torres Vedras flora of the Figueira da Foz Formation [122], as the oldest record of crown-Alismatales.
Phylogenetic justification. Magallón et al. [13] argue that these striate and inaperturate pollen grains are similar to those of Monsteroideae (Araceae), such as *Holochlamys* and *Spathiphyllum*.
Minimum age. 96.24 Ma
Age justification. The Torres Vedras flora has been considered Late Barremian to Early Aptian in age [71, 123]. However, recent evidence suggests that they are considerably younger, within the 'Upper Almargem' Formation overlying a late Aptian to early Albian unconformity [124]. Despite the uncertainty, an unequivocal minimum age is provided by the appearance of ostracod *Fossocytheridea merlensis* in the overlying Canecas Formation, attributable to the base of the Middle Cenomanian [125, 126], which coincides with the base of the Conlinoveras gilberti Zone, dated to 96.24 Ma [34].

Node 1228 | MRCA: *Alisma-Potamogeton* | 66 Ma.
Fossil taxon and specimen. *Cardstonia tolmanii* [UAPC-ALTA S55138: University of Alberta Paleobotanical Collections] from the St. Mary River Formation [127].
Phylogenetic justification. Ridley and Stockey (2004) provided a convincing argument that this fossil shows closest similarity to extant genera of Alismatales. This interpretation is accepted here.
Minimum age. 66 Ma.
Age justification. Riley and Stockey [127] attribute *Cardstonia tolmanii* to the Late Campanian–Early Maastrichtian (Upper Cretaceous), though there is little direct evidence to support this. However, the St. Mary River Formation is chronostratigraphically equivalent to the Horseshoe Canyon Formation into which it intergrades and which in turn is constrained minimally by the C30n magnetozone [128], the top of which is slightly older than the Maastrichtian-Paleogene Boundary, dated to 66.0 Ma [34].

Node 1232 | CG Araceae | MRCA: *Orontium-Xanthosoma* | 76 Ma.
Fossil taxon and specimen. *Lysichiton (Araciphyllites) austriacus* [NHMW 1999B0057/0183: Natural History Museum, Vienna, Austria], from the Grünbach Formation of Austria, is the oldest record of this clade [129, 130]
Phylogenetic justification. This fossil was interpreted as closely related to extant species of the Orontioideae (family Araceae) by Bogner et al. [129]. This assignment is supported by the occurrence of several putative related fossil taxa at around the same time [131].
Minimum age. 76 Ma.
Age justification. The Grunbach Formation can be attributed to the *Globotruncata elevata* planktic foram biozone and the UC15 calcareous nannofossil biozone [130, 132]. The overlying Piesting Formation has been attributed to the UC16 nannofossil biozone [130, 132] and, thus, the age of the Grunbach Flora can be constrained by the UC15-UC16 boundary which is estimated to be 76 Ma [34].

Node 1237 | CG Laurales | MRCA: *Laurus-Calycanthus* | 107.59 Ma.
Fossil taxon and specimen. *Virginianthus calycanthoides* [PP43703: Field Museum, Chicago IL, USA] from the Albian of Puddledock, Virginia [133].
Phylogenetic justification. Doyle and colleagues [104, 134] identify *Virginianthus calycanthoides* from the Albian of Puddledock, Virginia [133], as the sister group to Calycanthaceae, or to the remaining Laurales, in either instance the oldest record of crown Laurales. Massoni et al. [53] also recognise *Cohongarootonia hispida* from the Puddledock Flora [135] as the oldest record of the total group comprised of Lauraceae, Monimiaceae, and Hernandiaceae, in other words the crown node of the clade of Laurales excluding Calycanthaceae [53]. We accept both of these as records of crown-Laurales, deeming evidence of their membership of more derived clades as insufficiently robust to be used as a basis for calibration.
Minimum age. 107.59 Ma.
Age justification. Massoni et al. [53] reason that the sediments in the Puddledock Locality are definitively early Albian based on the presence of reticulate tricolpate pollen and *Clavatipollenites rotundus* (aff. *Retimonocolpites dividuus* [136] but not striate tricolpates, which occur later in the early Albian.

Node 1248 | CG Magnoliales | MRCA: *Magnolia- Myristica* | 110.87 Ma.
Fossil taxon and specimen. *Endressinia brasiliana* [MB. PB. 2001/1455: Museum of Natural History, Institute of Paleontology, Berlin, Germany], from the Crato Formation of Brazil [137].
Phylogenetic justification. Masson et al. identify both *Schenkeriphyllum glanduliferum* and *Endressinia brasiliana*, both from the Crato Formation of Brazil [137, 138], as the oldest records of crown Magnoliineae, the sister clade of Myristicaceae , based on the phylogenetic analyses [98, 134, 138].
Minimum age. 110.87 Ma.
Age justification. Clarke et al. [1] argued that the age of the Crato Formation could not be constrained to being definitively older than Albian based on pollen [140], ostracod [141], and dinoflagellate [142] biostratigraphy and, in the absence of further evidence, established a minimum constraint on the Albian-Cenomanian boundary. Massoni et al. [53] argued for an Aptian age for the Crato Formation based on evidence from Heimhofer and Hochuli [142] but, unfortunately, these authors do not present evidence that can discriminate against a possible early Albian age for the Crato Formation, as acknowledged by Mohr et al. [138]. While the evidence suggests at worst, an early Albian age for the Crato Formation, and so it is possible to derive a minimum age interpretation for the Formation based on the Early-Middle Albian Boundary, which coincides approximately with the base of the *Douvilleiceras mammillatum* ammonite biozone, dated to 110.87 Ma [34].
Discussion. *Archaeanthus linnenbergii* was recognized as a further putative stem group Magnoliaceae but it is younger than *Endressinia* [53, 98].

Node 1256 | SG Canellales | MRCA: *Piper-Canella* | 125.9 Ma.
Fossil taxon and specimen. *Walkeripollis gabonensis* [Single-pollen grain preparation 2963-27: University of California (Berkeley) Museum of Paleontology], Cocobeach sequence near N'Toum, Gabon [53].
Phylogenetic justification. Massoni et al. [53] identify *Walkeripollis gabonensis* as the oldest record of Canellales, based on phylogenetic of pollen grain characters that resolve this pollen species as stem-Winteraceae [134], one of the two families that comprise Canellales, based on the presence of permanent tetrads, rounded aperture shape, as well as characteristic pore sculpture.
Minimum age. 125.9 Ma.
Age justification. Massoni et al. [53] establish a pre-Aptian age for *Walkeripollis gabonensis* based on the correlation of its occurrence in the Elf-Aquitaine palynological Subzone C-VIIc of the Cocobeach sequence near N'Toum, Gabon, to better-dated late Barremian sequences elsewhere. Thus, a minimum age constraint is established on the Barremian-Aptian boundary that has been dated to 126.3 Ma ±0.4 Myr, thus, 125.9 Ma.
Discussion. Massoni et al. [53] proposed records of more derived clades within Canellales (e.g. *Appomattoxia*, including the pollen of *Tucanopollis* and *Transitoripollis* types, from the Puddledock, Virginia [143], and Torres Vedras, Portugal [71, 144]). However, these are not sufficiently convincing to use as a basis for calibration. For example, *Appomattoxia* maybe more closely related to *Ceratophyllum* and Chloranthaceae [98].

Node 1260 | SG Saururus | MRCA: *Saururus-Houttuynia* | 44.3 Ma.
Fossil taxon and specimen. *Saururus tuckerae* [UAPC P1631 Bbot a: University of Alberta (Edmonton) Paleobotanical Collections] from the Middle Eocene Princeton Chert, British Columbia, Canada.
Phylogenetic justification. Massoni et al. [53] follow Smith and Stockey [145] in identifying *Saururus tuckerae* as the oldest record of total group *Saururus*. Based on tens of flowers and a partial inflorescence, the flower structure and pollen are characteristic of Saururaceae (Piperales), and phylogenetic analyses resolved *S. tuckerae* as the sister clade to extant *Saururus* [145].
Minimum age. 44.3 Ma.
Age justification. The Princeton Chert is part of the Allenby Formation which has been the subject of a number of absolute dating studies yielding age estimates of 48 Ma ± 2 Myr [146, 147], between 47 Ma ± 2 Myr and 50 Ma ± 2 Myr [148], between 46.2 Ma ± 1.9 Myr and 49.4 Ma ± 2 Myr [149], and 52.08 Ma ± 0.12 Myr [150] for the Allenby Formation. We follow Massoni et al. [53] in basing our minimum constraint based on the youngest age Interpretation of the youngest radiometric age estimate, viz. 44.3 Ma

Node 1268 | CG Chloranthales | MRCA: *Chloranthus-Hedyosmum* | 92.8 Ma.
Fossil taxon and specimen. *Pennipolis* plant [151] based on material from Vale de Agua, Buarcos, Portugal [152].
Phylogenetic justification. *Pennipolis* was found as sister to the crown group Chloranthaceae [153], whereas the Aptian to Cernomanina *Asteropollis* mesofossils may represent both crown and stem group Chloranthaceae [98].
Minimum age. 92.8 Ma.
Age justification. Clarke et al. [1] established a minimum age for Vale de Agua, which is assigned to the Figueira da Foz Formation [154-156] based on the overlying Costa d'Arnes Formation, the oldest ammonites in

which include *Calycoceras naviculare* [157], indicative of the naviculare biozone, the dating of which in error of the top of the Cenomanian, dated 93.6 Ma ± 0.8 Myr [34], thus, 92.8 Ma.
Discussion. Other early Cretaceous fossils with possible relationships to Chloranthaceae, such as *Zlatkcarpus* and *Canrightia* are younger than *Pennipolis* [98].

Node 1272 | SG Schisandraceae | MRCA: Trimenia-Kadsura | 107.59 Ma.
Fossil taxon and specimen. *Anacostia virginiensis* based on material from Kenilworth, Maryland, Puddledock, Virginia .
Phylogenetic justification. The assignment is based on the result of maximum parsimony based reconstruction of the phylogenetic relationships of this fossil to extant angiosperms that recovered this taxon as nested between *Trimenia* and the clade comprising *Illicium* and *Schisandra* [153].
Minimum age. 107.59 Ma.
Age justification. Massoni et al. [53] reason that the sediments in the Puddledock Locality are definitively early Albian based on the presence of reticulate tricolpate pollen and *Clavatipollenites rotundus* (aff. *Retimonocolpites dividuus* [136]) but not striate tricolpates, which occur later in the early Albian. Therefore, they constrain minimally the age of the *A. virginiensis* by the Middle-late Albian boundary, which coincides with the base of the *Diploceras cristatum* biozone which has been dated to 107.59 Ma [34].
Discussion. *Anacostia*, reportedly from the early and middle Albian of Buarcos, Famalicão, and Vale de Agua (Portugal), Puddledock (Virginia, USA), and Kenilworth (Maryland, USA) was recognized as the oldest fossil record of the Austrobaileyales [98, 153]. Doyle and Endress [153] *identified Anacostia portugallica* and *A. teixeirae* as early Albian and, therefore the oldest species belonging to this lineage. However, the minimum age interpretation of these localities the Figueira da Foz Formation cannot be constrained minimally to more than 92.8 Ma (see above). However, the minimum age constraint on *A. virginiensis* from the Puddledock Locality is older.

Node 1273 | SG *Illicium* | MRCA: *Illicium-Schisandra* | 85.44 Ma.
Fossil taxon and specimen. *Illiciospermum pusillum* [1700b-127: Komarov Botanical Institute, St. Petersburg, Russia.], known from seeds from the Cenomanian-Turonian of Kazahkstan [158].
Phylogenetic justification. *Illiciospermum pusillum,* known from seeds that preserve a structure resembling the strophiole of *Illicium* [158].
Minimum age. 85.44 Ma.
Age justification. There is a paucity of evidence supporting the age interpretation of the Sarbay Quarry near Rudnyy, Kustanay Region, north-western Kazakhstan. Frumin and Friis (1996) describe the sediments, including the plant-bearing bed, as belonging to the Shet-Irgiz Formation of Cenomanian-Turonian age [159], overlain by marine sands of the Santonian-Campanian Ayat Suite containing *Inoceramus cardissoides* [159]. The *Inoceramus cardissoides* Zone falls fully within the *Sigalia carpatica* Planktic foraminiferal Zone [160], the range end of which is dated to 85.44 Ma [34].

Node 1276 | SG Cabombaceae | MRCA: *Nymphaea-Cabomba* | 110.87 Ma.
Fossil taxon and specimen. *Pluricarpellatia peltata* [MB.Pb. 2000/80: Museum of Natural History, Berlin, Germany], from the Crato Formation of Brazil [161]
Phylogenetic justification. *Pluricarpellatia peltata* has been considered phylogenetically and resolved as members of the lineage leading to *Cabomba* after it diverged from *Nymphaea* [162].
Minimum age. 110.87 Ma.
Age justification. Clarke et al. [1] argued that the age of the Crato Formation could not be constrained to being definitively older than Albian based on pollen [140], ostracod [141], and dinoflagellate [142] biostratigraphy and, in the absence of further evidence, established a minimum constraint on the Albian-Cenomanian boundary. Massoni et al. [53] argued for an Aptian age for the Crato Formation based on evidence from Heimhofer and Hochuli [142] but, unfortunately, these authors do not present evidence that can discriminate against a possible early Albian age for the Crato Formation, as acknowledged by Mohr et al. [138]. While the evidence suggests, at worst, an early Albian age for the Crato Formation, it is possible to derive a minimum age interpretation for the Formation based on the Early-Middle Albian Boundary, which coincides approximately with the base of the *Douvilleiceras mammillatum* ammonite biozone, dated to 110.87 Ma [34].
Discussion. Magallon et al. [13] derive a minimum constraint from *Monetianthus mirus* which they recognize as a representative of the Nymphaeaceae stem lineage and, thus, use it as the basis of a minimum constraint on the age of total-group Nymphaceaceae at 125 Ma. However, Clarke et al. [1] demonstrated that the minimum age of the host deposit, Vale de Água, Portugal [163, 164] is 93.9 Ma [34]. However, there are other, potentially older records of Nymphaeaceae and, more specifically, the crown clade circumscribed by *Nymphaea-Cabomba*. Clarke et al. [1] identified much older, but more equivocal records, as well as the oldest unequivocal records, viz. *Pluricarpellatia peltata* from the Crato Formation of Brazil [161] and *Scutifolium jordanicum* from the

Jarash Formation (Kurnub Group) of Jordan [162], both of which have been considered phylogenetically and resolved as members of the lineage leading to *Cabomba* after it diverged from *Nymphaea* [162]. *Scutifolium jordanicum* was used to establish a minimum age for crown-Nymphaeales at 105 Ma by Smith et al. [165], and for total-group Cabombaceae at 105 Ma by Zanne et al. [166]. The Jarash Formation can be dated minimally to 95 Ma (96.1 Ma ± 1.1 Myr in [167], but the Crato Formation is older .

Node 1278 | CG Nymphaeaceae | MRCA: *Nymphaea - Nuphar* crown | 92.8 Ma.
Fossil taxon and specimen. *Monetianthus mirus* [S122015: Palaeobotanical Collectons, Swedish Museum of Natural History, Stockholm, Sweden] from Vale de Agua, Portugal [164].
Phylogenetic justification. Doyle and Endress [153] identify *Monetianthus mirus*, a coalified flower from Vale de Agua, Portugal, as a stem member of the clade Barclaya-Nymphaeoideae, to the exclusion of *Nuphar*.
Minimum age. 92.8 Ma.
Age justification. Clarke et al. [1] established a minimum age for Vale de Agua, which is assigned to the Figueira da Foz Formation [154-156], based on the overlying Costa d'Arnes Formation, the oldest ammonites in which include *Calycoceras naviculare* [157], indicative of the *naviculare* Biozone, the dating of which in error of the top of the Cenomanian, dated 93.6 Ma ± 0.8 Myr [34], thus, 92.8 Ma.

Node 1279 | CG Acrogymnosperms | MRCA: *Ginkgo-Pinus* | 308.14 Ma – 365.629 Ma.
Fossil taxon and specimen. *Cordaites iowensis* [UM4616: University of Michigan and Illinois Geological Survey, Ann Arbor MI, USA] from the Laddsdale Coals (Cherokee Group, Desmoinesian) near What Cheer, Iowa, USA [22].
Phylogenetic justification. Clarke *et al*. [1] identify cordaitean coniferophytes as the oldest records of the *Ginkgo-Pinus* clade, the oldest whole plant reconstruction of which is *Cordaites iowensis* from the Laddsdale Coals (Cherokee Group, Desmoinesian) near What Cheer, Iowa [22].
Minimum age. 308.14 Ma.
Maximum age: 365.629 Ma.
Age justification. Janousek and Pope [23] argue that the Laddsdale Coal is equivalent to the Bluejacket Coal of Oklahoma, which occurs as part of the Bluejacket Sandstone Member, underlying the Inola Limestone, part of the Inola Cyclothem of the Krebs subgroup of the Cherokee Group, characterized by the occurrence of the conodonts *Idiognathodus amplificus, Idiognathodus podolskensis and Neognathodus asymmetricus* [24]. The Inola cyclothem falls fully within the *Idiognathodus amplificus/ Idiognathodus obliquus* biozone [25]. This is indicative of the *Neognathodus medexultimus-Streptognathodus concinnus* (Pc10) biozone, certainly older than the *Neognathodus roundyi – Streptognathodus cancellosus* (Pc11) biozone [25, 26]. The base of Pc10 is bracketed by an older age constraint of 312.01 Ma ± 0.37 Myr and the base of Pc11 is bracketed by a younger age constraint of 308.5 Ma ± 0.36 Myr in the Composite Standard of Davydov et al. [26], yielding a minimum age constraint of 308.14 Ma. A soft maximum is based upon the first appearance of seeds in the form of preovules which are attributable to the spermatophyte stem, the oldest interpretation of which is 365.629 Ma (see Spermatophyta).
Discussion. Zanne et al. [166] derive a minimum constraint from *Emporia lockardii* at 290.0 Ma which they recognize as a member of crown-Acrogymnospermae within a phylogenetic concept of the group in which, as here, cycads and *Ginkgo* comprise a clade.

Node 1280 | CG Conifers | MRCA: Pinus-Metasequoia | 147 Ma - 312.38 Ma.
Fossil taxon and specimen. *Araucaria mirabilis* [NHM V. 30953: Natural History Museum, London, UK], represented by cones, from Cerro Cuadrado petrified forest, La Matilde Formation, Patagonia, Argentina [168-171].
Phylogenetic justification. These fossils possess a 'vascular plexus' at the ovule base, ovuliferous scale vascularization, two vascular strands to the conescale complex and an embryo with two cotyledons, all characters established to distinguish *Araucaria* section *Bunya* of the Araucariaceae [170, 172], to which only extant *Araucaria bidwillii* belongs.
Minimum age. 147 Ma.
Maximum age: 312.38 Ma.
Age justification. The age of La Matilde Formation is poorly constrained as the stratigraphy is complex, although the volcanic deposits do allow radiometric dating. La Matilde Formation is overlain by volcanics dated to 157 Ma ± 10 Myr [173], and thus the minimum constraint on the divergence of crown Cupressophyta, total group Cupressophyta and crown Coniferae is 147 Ma. A soft maximum constraint can be based on *Cordaites iowensis*, a cordaitean coniferophyte from the Laddsdale Coals (Cherokee Group, Desmoinesian) near What Cheer, Iowa [22], is the oldest whole plant reconstruction for Coniferae. Janousek and Pope [23] argue that the Laddsdale Coal is equivalent to the Bluejacket Coal of Oklahoma, which occurs as part of the Bluejacket Sandstone Member, underlying the Inola Limestone, part of the Inola Cyclothem of the Krebs subgroup of the

Cherokee Group, characterized by the occurrence of the conodonts *Idiognathodus amplificus, Idiognathodus podolskensis and Neognathodus asymmetricus* [24]. The Inola cyclothem falls fully within the *Idiognathodus amplificus/ Idiognathodus obliquus* biozone [25]. This is indicative of the *Neognathodus medexultimus-Streptognathodus concinnus* (Pc10) biozone, certainly older than the *Neognathodus roundyi – Streptognathodus cancellosus* (Pc11) biozone [25, 26]. The base of Pc10 is bracketed by an older age constraint of 312.01 Ma ± 0.37 Myr and the base of Pc11 is bracketed by a younger age constraint of 308.5 Ma ± 0.36 Myr in the Composite Standard of Davydov et al. [26], yielding a soft maximum of 312.38 Ma.
Discussion. This is the fundamental divergence of Coniferae into Cupressophyta, Gnetales and Pinaceae. The oldest secure records of the gnepine total group occur within the Yixian Formation of Liaoning, China, the minimum age of which is 121.8 Ma (see [1]). The oldest possible records of Cupressophyta total group include Triassic *Rissikia media* (Townrow, 1967) but it lacks the Podocarpaceae diagnostic feature of one ovule per cone scale, instead possessing two [1]. Other Triassic-Jurassic records are equally problematic [174-176].

Node 1282 | CG Gnetales | MRCA: *Gnetum-Welwitschia* | 119.6 Ma – 312.38 Ma.
Fossil taxon and specimen. *Eoantha zherikhinii* [Repository of the Institute of Biology and Pedology, Vladivostok, Russia], from the Zaza Formation at the Baisa locality in the upper reaches of the Vitim River in Lake Baikal [177].
Phylogenetic justification. This fossil is assigned as a representative of the Gnetales because of the presence of an ovule with a an exending micropylar tube similar to extend Gnetales and the presence of polyplicate pollen of *Ephedritopes*-type (see [178]). *Eoantha* is one out of several Early Cretaceous fossils unequivocally assigned to the crown Gnetales [179]
Minimum age. 119.6 Ma.
Maximum age. 312.38 Ma.
Age justification. The Zaza Formation can be correlated with the Turga Formation, also of Transbaikalia based principally on common elements of their floral assemblages, including *Asteropollis asteroids*, *Dicotylophyllum pusilum*, *Baisa hirsuita, Podozamites, Schizolepis, Pseudolarix, Phoenicopsis, Czekanowskia rigida* and *Sphenobaiera* [177, 180-182]. The age of the Turga flora and Formation is based on the chronological distribution of *Asteropollis* type pollen, but correlation with the Yixian Formation of China is also supported strongly [180], allowing for refinement of the *Asteropollis*-derived ages. Correlation between Turga and Yixian is based on similarities in the floral assemblages of these two formations, with the shared presence of the species *Baisa hirsuita, Botrychites reheensis, Neozamites verchojanensis, Pityolepis pseudotsugaoides, Brachyphyllum longispicum, Scarbugia hilii, Ephedrites chenii, Carpolithus multiseminalis, Carpolithus pachythelis, Schizolepis, Baiera, Coniopteris, Ginkoites, Pityocladus* , *Pityospermum* and *Elatocladus* [177, 180, 183]. The shared presence of *Asteropollis asteroides* in Turga and Zaza can be used to constrain their age. The last appearance of *Asteropollis* pollen is in Antarctica [184] and is dated to the end-Campanian, at the latest 72.1 Ma ± 0.2 [34]. This minimum may be constrained further based on the correlation of the Zaza Formation through the Turga Formation to the Yixian Formation. The main fossil bearing beds in the Yixian Formation have been recently dated and may be as old as 129.2 Ma [41], however, in the absence of knowledge of the position of the fossils within the stratigraphy, relative to the sources of the absolute dates, a minimum age constraint can be derived from the Jiufontang Formation which overlies it. ^{40}Ar/^{39}Ar dating of a number of samples from the Jiufontang Formation has yielded an age of 120.3 ± 0.7 Ma for the volcanic tuffs [42], establishing a minimum constraint of 119.6 Ma for the age of the Yixian, Formation and, thus ultimately the Zaza Formation.

A soft maximum constraint can be based on *Cordaites iowensis*, a cordaitean coniferophyte from the Laddsdale Coals (Cherokee Group, Desmoinesian) near What Cheer, Iowa [22], is the oldest whole plant reconstruction for Coniferae. Janousek and Pope [23] argue that the Laddsdale Coal is equivalent to the Bluejacket Coal of Oklahoma, which occurs as part of the Bluejacket Sandstone Member, underlying the Inola Limestone, part of the Inola Cyclothem of the Krebs subgroup of the Cherokee Group, characterized by the occurrence of the conodonts *Idiognathodus amplificus, Idiognathodus podolskensis and Neognathodus asymmetricus* [24]. The Inola cyclothem falls fully within the *Idiognathodus amplificus/ Idiognathodus obliquus* biozone [25]. This is indicative of the *Neognathodus medexultimus-Streptognathodus concinnus* (Pc10) biozone, certainly older than the *Neognathodus roundyi – Streptognathodus cancellosus* (Pc11) biozone [25, 26]. The base of Pc10 is bracketed by an older age constraint of 312.01 Ma ± 0.37 Myr and the base of Pc11 is bracketed by a younger age constraint of 308.5 Ma ± 0.36 Myr in the Composite Standard of Davydov et al. [26], yielding a soft maximum of 312.38 Ma.

Node 1284 | MRCA: *Ginkgo-Cycas* | 264.7 Ma – 365.629 Ma.
Fossil taxon and specimen. *Crossozamia chinensis* [GP0027: Beijing Graduate School, China Institute of Mining, Beijing, China], Lower Shihhotse Formation at Simugedong, Dongshan (East Hills), Taiyuan, north China [185].

Phylogenetic justification. Nagalingum et al. [186] identify *Crossozamia* as the oldest record of the *Cycas* lineage, based on megasporophylls that exhibit similarity to extant *Cycas* [185]. They argue against the interpretation of *Crossozamia* as the sister lineage of *Cycas* based on the presence of an estipulate leaf base and a terminal pinna found in the seedlings [187], instead favouring its assignment to the cycad stem. The arguments presented clearly raise doubts about the assignment of *Crossozamia* to crown-cycads, however, they do not provide definitive evidence of its exclusion from this clade and so *Crossozamia* may more appropriately be assigned to the cycad total group (i.e. we cannot discriminate between a stem or crown-cycad affinity based on the available evidence). In either instance, *Crossozamia* is the oldest record of the minimal clade comprised of *Gingko* and *Cycas*.
Minimum age. 264.7 Ma.
Maximum age. 365.629 Ma.
Age justification. The Lower Shihhotse Formation at Simugedong, Dongshan (East Hills), Taiyuan, north China [185] has been established biostratigraphically as Roadian-Wordian (middle Permian) [188] and, thus a minimum age constraint can be established on the Wordian-Capitanian Boundary which has been dated to 265.1 Ma ± 0.4 Myr [189]. Thus, the minimum age constraint on the *Cycas-Ginkgo* clade is 264.7 Ma. A soft maximum is based upon the first appearance of seeds in the form of preovules which are attributable to the spermatophyte stem, the oldest interpretation of which is 365.629 Ma (see Spermatophyta).

Node 1286 | CG Ferns | MRCA: *Psilotum-Adiantum* | 384.71 Ma – 451 Ma.
Fossil taxon and specimen. *Ibyka amphikoma* was recovered from the Manorkill Shale Member at Schoharie Creek directly below the spillway of Gilboa dam, Gilboa, Schoharie County, New York, Gilboa [14].
Phylogenetic justification. *Ibyka amphikoma* [14] is the oldest record of the equisetopsid lineage based on the phylogenetic analyses undertaken by Kenrick and Crane [3].
Minimum age. 384.71 Ma.
Maximum age. 451 Ma.
Age justification. *Ibyka amphikoma* was recovered from the Manorkill Shale Member, which is a lateral equivalent of the Windom Member, within the Moscow Formation of New York [18, 19], which falls fully within the *ansatus* conodont Biozone [20, 21] the top of which is dated to 385.41 Ma ± 0.7 Myr , thus, yielding a minimum age constraint of 384.71 Ma. The soft maximum constraint, following Clarke et al. [1], is based on the oldest occurrences of trilete spores, known from the Qusaiba-1 core from the Quasim Formation of northern Saudi Arabia [8]. The very oldest records preceed the earliest occurrences of the *A. barbata*, opening the possibility that they occur in the preceding *Tanuchitina fistulosa* biozone, though *T. fistulosa* does not occur. The oldest stratigraphic records within the core co-occur with the chitinozoan *Armoricochitina nigerica*, known to extend into the Caradoc, to within the biozone characterized by *Fungochitina spinifera* (= *Fungochitina fungiformis*) [9]. The base of the *F. spinifera* Zone falls within the *Dicranograptus clingani* Biozone (*Dicellograptus morrisi* Subzone) [10], the base of which is estimated at 451 Ma [11]. Thus, we establish our soft maximum for Tracheophyta at 451 Ma.
Discussion. Magallón et al. [13] established a minimum age constraint based on *Ibyka amphikoma* using the Givetian-Frasnian boundary, for which they provided a date of 385 Ma, though this has since been revised to 382.7 Ma ± 1 Myr [17]. However, we provide a more detailed stratigraphic justification for the age of *I. amphikoma* which allows for an older minimum age constraint.

Node 1287 | CG Lycophytes | MRCA: *Huperzia-Selaginella* | 392.1 Ma – 451 Ma.
Fossil taxon and specimen. *Leclercquia complexa* [CW092 (07 – 061): Collections of the Centre for Palynological Studies, Department of Animal and Plant Sciences, University of Sheffield, UK], from Campbellton Formation outcropping on the south shore of the Restigouche River, between Dalhousie and Campbellton, New Brunswick, eastern Canada [190].
Phylogenetic justification. Kenrick and Crane [3] identified *Leclercquia complexa* as the oldest member of Isoetopsida and crown Lycopodiophyta. This interpretation is supported by spore characteristics analysed phylogenetically by Wellman et al. [190].
Minimum age. 392.1 Ma.
Maximum age. 451 Ma.
Age justification. A Late Emsian age is often cited for the New Brunswick occurrences of identified *Leclercquia complexa* and, indeed, the *Stockmensella-Leclerqia* macroplant Biozone spans all but the earliest Emsian [17]. However, Wellman et al. [190] attribute their own material of *Leclercquia complexa* to the middle of the *Emphanisporites annulatus – Camarozonotriletes sextantii* Spore Assemblage Biozone which falls within the early part of the Emsian. In either instance, the earliest records of *Leclercquia complexa* fall fully within the Emsian, the end of which is dated to 393.3 Ma ± 1.2 Myr [17], yielding a minimum constraint of 392.1 Ma. The soft maximum constraint, following Clarke et al. (2011), is based on the oldest occurrences of trilete spores, known from the Qusaiba-1 core from the Quasim Formation of northern Saudi Arabia [8]. The very oldest

records precede the earliest occurrences of the *A. barbata*, opening the possibility that they occur in the preceding *Tanuchitina fistulosa* biozone, though *T. fistulosa* does not occur. The oldest stratigraphic records within the core co-occur with the chitinozoan *Armoricochitina nigerica*, known to extend into the Caradoc, to within the Biozone characterized by *Fungochitina spinifera* (= *Fungochitina fungiformis*) [9]. The base of the *F. spinifera* Zone falls within the *Dicranograptus clingani* Biozone (*Dicellograptus morrisi* Subzone) [10], the base of which is estimated at 451 Ma [11]. Thus, we establish our soft maximum for Tracheophyta at 451 Ma. **Discussion.** Magallon et al. [13] cite a minimum age of 385 Ma, based on the Middle-Upper Devonian Boundary, but our more detailed stratigraphy allows for an older minimum age interpretation of *Leclercquia complexa.*

Supplementary References

1. **Clarke J, Donoghue PCJ, Warnock RCM. 2011.** Establishing a timescale for plant evolution. *New Phytologist* **192**(1), 266-301.
2. **Kotyk ME, Basinger JF, Gensel PG, de Freitas TA. 2002.** Morphologically complex plant macrofossils from the Late Silurian of Arctic Canada. *American Journal of Botany* **89**(6), 1004-1013.
3. **Kenrick P, Crane PR. 1997.** *The origin and early diversification of land plants: a cladistic study*. Washington D.C., Smithsonian Institution Press.
4. **Klapper G, Murphy MA. 1974.** Silurian - lower Devonian conodont sequence in the Roberts Mountains Formation of central Nevada. *University of California Publications in Geological Sciences* **111**, 1-62.
5. **Thorsteinsson R. 1980.** Stratigraphy and conodonts of Upper Silurian and Lower Devonian rocks in the environs of the Boothia Uplift Canadian Arctic Archipelago. 1. Contributions to stratigraphy. *Geological Survey of Canada Bulletin* (292), VIII-38.
6. **Uyeno TT. 1990.** Biostratigraphy and conodont faunas of Upper Ordovician through Middle Devonian rocks, eastern Arctic Archipelago. *Geological Survey of Canada, Bulletin* **401**, 1-211.
7. **Mayr U, Brent TA, de Freitas T, Frisch T, Nowlan GS, Okulitch AV. 2004**. Geology of Eastern Prince of Wales Island and Adjacent Smaller Islands, Nunavut. *Geological Survey of Canada, Bulletin* **574**, 1-88.
8. **Steemans P, Le Herisse A, Melvin J, Miller MA, Paris F, Verniers J, Wellman CH. 2009.** Origin and Radiation of the Earliest Vascular Land Plants. *Science* **324**(5925), 353-353.
9. **Paris F, Le Herisse A, Monod O, Kozlu H, Ghienne J-F, Dean WT, Vecoli M, Gunay Y. 2007**. Ordovician chitinozoans and acritarchs from southern and southeastern Turkey. *Revue de micropaléontologie* **50**(1), 81-107.
10. **Vandenbroucke TRA, Williams M, Zalasiewicz JA, Davies JR, Waters RA. 2008.** Integrated Upper Ordovician graptolite-chitinozoan biostratigraphy of the Cardigan and Whitland areas, southwest Wales. *Geological Magazine* **145**, 199-214.
11. **Cooper RA, Sadler PM. 2012.** The Ordovician Period. In *Geologic timescale 2012* (eds. Gradstein FM, Ogg JG, Schmitz M, Ogg G), pp. 489-523, Elsevier.
12. **Bonamo PM. 1977**. *Rellimia Thomsonii* (Progymnospermopsida) from Middle Devonian of New York State. *American Journal of Botany* **64**(10), 1272-1285.
13. **Magallón S, Hilu KW, Quandt D. 2013.** Land plant evolutionary timeline: gene effects are secondary to fossil constraints in relaxed clock estimation of age and substitution rates. *Am J Bot* **100**(3), 556-573.
14. **Skog JE, Banks HP. 1973.** *Ibyka amphikoma*, gen et sp-n - new protoarticulate precursor from late Middle Devonian of New York State. *American Journal of Botany* **60**(4), 366-380.
15. **Bartholomew AJ, Brett CE. 2007.** Correlation of Middle Devonian Hamilton Group-equivalent strata in east-central North America: implications for eustasy, tectonics and faunal provinciality. *Geological Society, London, Special Publications* **278**(1), 105-131.
16. **Johnson JG, Klapper G, Sandberg CA 1985.** Devonian eustatic fluctuations in Euramerica. *Geological Society of America Bulletin* **96**(5), 567.
17. **Becker RT, Gradstein FM, Hammer O. 2012.** The Devonian Period. In *The geological timescale 2012* (eds. Gradstein F.M., Ogg J.G., Schmitz M., Ogg G.), pp. 559-601, Elsevier.
18. **Fisher DW., Isachsen YW., Rickard LV, Broughton JG, Offield TW. 1962.** *Geologic map of New York.* Albany, New York State Museum Sciece Service, Geological Survey.
19. **Rickard LV. 1964.** *Correlation of the Devonian rocks in New York State. Map and Chart Series 4*. Albany, New York State Musuem Science Service Geological Survey.
20. **Klapper G. 1981.** Review of New York Devonian conodont biostratigraphy. In *Devonian biostratigraphy of New York, Part I* (eds. Oliver W.A., Jr., Klapper G.), pp. 57-68, IUGS SDS.
21. **Kirchgasser WT. 2000.** Correlation of stage boundaries in the Appalachian Devonian, Eastern United States. *Courier Forschungsinstitut Senckenberg* **225**, 271-284.

22. **Trivett ML 1992.** Growt, architecture, structure, and relationships of *Cordaixylon iowensis* nov comb (Cordaitales). *International Journal of Plant Sciences* **153**(2), 273-287.
23. **Janousek TJ, Pope JP. 2014.** Petrology, petrography and conodont biostratigraphy of the Laddsdale Coal interval, along Whitebreast Creek, Bauer, Iowa. *GSA North-Central Section, 48th Annual Meeting, Abstracts* **16-5**.
24. **Heckel PH. 2013.** Pennsylvanian stratigraphy of Northern Midcontinent Shelf and biostratigraphic correlation of cyclothems. *Stratigraphy* **10**, 3-39.
25. **Barrick JE, Lambert LL, Heckel PH, Rosscoe SJ, Boardman DR. 2013.** Midcontinent Pennsylvanian conodont zonation. *Stratigraphy* **10**, 55-72.
26. **Davydov VI, Korn D, Schmitz MD. 2012.** The Carboniferous Period. In *The geologic time scale 2012* (eds. Gradstein F.M., Ogg J.G., Schmitz M., Ogg G.), pp. 603-651, Elsevier.
27. **Prestianni C. 2005.** Early diversification of seeds and seed-like structures. *Carnets De Geologie*, 33-38.
28. **Rothwell GW, Scheckler SE, Gillespie WH. 1989.** *Elkinsia* gen nov, a late Devonian gymnospermn with cupulate ovules. *Botanical Gazette* **150**(2), 170-189.
29. **Streel M, Scheckler SE. 1990.** Miospore lateral distribution in upper Fammenian alluvial lagoonal to tidal facies from eastern United States and Belgium. *Review of Palaeobotany and Palynology* **64**(1-4), 315-324.
30. **Streel M, Higgs K, Loboziak S, Riegel W, Steemans P. 1987.** Spore stratigraphy and correlation with faunas and floras in the type marine Devonian of the Ardenne-Rhenish regions. *Review of Palaeobotany and Palynology* **50**(3), 211-229.
31. **House MR., Gradstein FM. 2004.** The Devonian Period. In *A geologic timescale 2004* (eds. Gradstein FM, Ogg JG, Smith AG), pp. 202-221. Cambridge, Cambridge University Press.
32. **Hughes NF, McDougall AB. 1990.** Barremian-Aptian angiospermid pollen records from southern England. *Review of Palaeobotany and Palynology* **65**(1-4), 145-151.
33. **Judd WS, Olmstead RG. 2004.** A survey of tricolpate (eudicot) phylogenetic relationships. *American Journal of Botany* **91**(10), 1627-1644.
34. **Ogg JG., Hinnov LA. 2012.** Cretaceous. In *The geologic time scale 2012* (eds. Gradstein FM, Ogg JG, Schmitz M, Ogg G), pp. 793-853, Elsevier.
35. **Ogg JG. 2012.** Triassic. In *The geologic time scale 2012* (eds. Gradstein FM, Ogg JG, Schmitz M, Ogg G), pp. 681-730, Elsevier.
36. **Liu ZJ, Wang X. 2016.** A perfect flower from the Jurassic of China. *Hist Biol* **28**(5), 707-719.
37. **Han G, Liu Z, Liu X, Mao L, Jacques FMB., Wang X. 2016.** A whole plant herbaceous angiosperm from the Middle Jurassic of China. *Acta Geologica Sinica* **90**, 19-29.
38. **Liu Z-J, Wang X. 2016.** Yuhania: a unique angiosperm from the Middle Jurassic of Inner Mongolia, China. *Historical Biology*, 1-11.
39. **Dilcher DL., Sun G, Ji Q, Li HQ. 2007.** An early infructescence Hyrcantha decussata (comb. nov.) from the Yixian Formation in northeastern China. *Proceedings of the National Academy of Sciences of the United States of America* **104**(22), 9370-9374.
40. **Wang W, Dilcher DL, Sun G, Wang H-S, Chen Z-D. 2016.** Accelerated evolution of early angiosperms: Evidence from ranunculalean phylogeny by integrating living and fossil data. *Journal of Systematics and Evolution* **54**(4), 336-341.
41. **Chang S-C., Zhang H, Hemming SR, Mesko GT, Fang Y. 2012.** Chronological evidence for extension of the Jehol Biota into Southern China. *Palaeogeography, Palaeoclimatology, Palaeoecology* **344–345**(0), 1-5.
42. **He HY, Wang XL, Zhou ZH, Wang F, Boven A, Shi G.H, Zhu RX. 2004.** Timing of the Jiufotang Formation (Jehol Group) in Liaoning, northeastern China, and its implications. *Geophysical Research Letters* **31**, 1-4.
43. **Jaramillo CA., Dilcher DL. 2001.** Middle Paleogene palynology of Central Colombia, South America: A study of pollen and spores from tropical latitudes. *Palaeontographica Abteilung B* **258**(4-6), 87-213.
44. **Germeraad JH., Hopping CA., Muller J. 1968.** Palynology of Tertiary sediments from tropical areas. *Review of Palaeobotany and Palynology* **6**, 189-348.
45. **Davis CC, Schaefer H, Xi Z, Baum D.A, Donoghue MJ, Harmon LJ. 2014.** Long-term morphological stasis maintained by a plant-pollinator mutualism. *Proceedings of the National Academy of Sciences of the United States of America* **111**(16), 5914-5919.
46. **Jaramillo CA, Rueda M, Torres V. 2010.** A palynological zonation for the Cenozoic of the Llanos and Llanos foothills of Colombia. *Palynology* **35**(1), 46-84.
47. **Boucher LD, Manchester SR, Judd WS. 2003.** An extinct genus of Salicaceae based on twigs with attached flowers, fruits, and foliage from the Eocene Green River Formation of Utah and Colorado, USA. *American Journal of Botany* **90**(9), 1389-1399.

48. **Manchester SR., Judd WS., Handley B. 2006.** Foliage and fruits of early poplars (Salicaceae: Populus) from the Eocene of Utah, Colorado, and Wyoming. *International Journal of Plant Sciences* **167**(4), 897-908.
49. **Smith ME, Carroll AR., Singer BS. 2008.** Synoptic revision of a major ancient lake system: Eocene Green River Formation, western United States. *GSA Bulletin* **120**, 54-84.
50. **Vandenberghe N, Hilgen FJ, Speijer RP. 2012.** The Paleogene Period. In *The geologic timescale 2012* (eds. Gradstein F.M., Ogg J.G., Schmitz M., Ogg G.), pp. 855-921. Ansterdam, Elsevier.
51. **Crepet WL, Nixon KC. 1998.** Fossil Clusiaceae from the Late Cretaceous (Turonian) of New Jersey and implications regarding the history of bee pollination. *American Journal of Botany* **85**(8), 1122-1133.
52. **Ruhfel BR, Stevens PF, Davis CC. 2013.** Combined Morphological and Molecular Phylogeny of the Clusioid Clade (Malpighiales) and the Placement of the Ancient Rosid Macrofossil Paleoclusia. *International Journal of Plant Sciences* **174**(6), 910-936.
53. **Massoni J, Doyle JA., Sauquet H. 2014.** Fossil calibration of Magnoliidae, an ancient lineage of angiosperms. *Palaeontologica Electronica*.
54. **Dettmann ME, Playford G. 1968.** Taxonomy of some Cretaceous spores and pollen grains from Eastern Australia. *Proceedings of the Royal Society of Victoria* **81**, 69-83.
55. **Sauquet H, Ho SY, Gandolfo MA, Jordan GJ, Wilf P, Cantrill DJ, Bayly MJ, Bromham L, Brown GK. Carpenter RJ, et al. 2012.** Testing the impact of calibration on molecular divergence times using a fossil-rich group: the case of Nothofagus (Fagales). *Syst Biol* **61**(2), 289-313.
56. **Partridge AD. 2006.** Late Cretaceous — Cenozoic palynology zonations Gippsland Basin. In *Australian Mesozoic and Cenozoic Palynology Zonations – updated to the 2004 Geologic Time Scale Geoscience Australia Record 2006/23* (ed. Monteil E), Geoscience Australia Record.
57. **Manchester SR, Dillhoff RM. 2004.** Fagus (Fagaceae) fruits, foliage, and pollen from the Middle Eocene of Pacific Northwestern North America. *Canadian Journal of Earth Sciences* **82**, 1509-1517.
58. **Renner SS., Grimm GW, Kapli P, Denk T. 2016.** Species relationships and divergence times in beeches: new insights from the inclusion of 53 young and old fossils in a birth-death clock model. *Philosophical transactions of the Royal Society of London Series B, Biological sciences* **371**(1699).
59. **Ewing TE. 1981.** Regional stratigraphy and structural setting of the Kamloops Group, South-central British Columbia. *Canadian Journal of Earth Sciences* **18**, 1464-1477.
60. **Moss PT, Greenwood DR, Archibald SB. 2005.** Regional and local vegetation community dynamics of the Eocene Okanagan Highlands (British Columbia – Washington State) from palynology. *Canadian Journal of Earth Sciences* **42**(2), 187-204.
61. **Pigg KB, DeVore Melanie L, Wojciechowski MF. 2008.** *Paleosecuridaca curtisii* gen. et sp. nov., Securidaca-like Samaras (Polygalaceae) from the Late Paleocene of North Dakota and their significance to the divergence of families within the Fabales. *International Journal of Plant Sciences* **169**(9), 1304-1313.
62. **Crane PR, Manchester SR, Dilcher DL 1990.** A preliminary survey of fossil leaves and well-preserved reproductive structures from the Sentinel Butte Formation (Paleocene) near Almont, North Dakota. *Fieldiana Geology* **20**(1418).
63. **Zetter R, Farabee MJ, Pigg KB, Manchester SR, DeVore ML., Nowak MD. 2011.** Palynoflora of the late Paleocene silicified shale at Almont, North Dakota, USA. *Palynology* **35**(2), 179-211.
64. **McClain AM, Manchester SR. 2001.** Dipteronia (Sapindaceae) from the Tertiary of North America and Implications for the Phytogeographic History of the Aceroideae. *American Journal of Botany* **88**(7), 1316-1325.
65. **Qiu YL, Li LB, Wang B, Chen ZD, Dombrovska O, Lee J, Kent L, Li RQ, Jobson RW, Hendry TA, et al. 2007.** A nonflowering land plant phylogeny inferred from nucleotide sequences of seven chloroplast, mitochondrial, and nuclear genes. *International Journal of Plant Sciences* **168**(5), 691-708.
66. **Manchester SR, Chen Z-D, Lu A-M, Uemura K. 2009.** Eastern Asian endemic seed plant genera and their paleogeographic history throughout the Northern Hemisphere. *Journal of Systematics and Evolution* **47**(1), 1-42.
67. **Nichols DJ, Ott HL. 1978.** Biostratigraphy and evolution of the *Momipites-Caryapollenites* lineage in the early Tertiary in the Wind River Basin, Wyoming. *Palynology* **2**, 93-112.
68. **Peppe DJ. 2010.** Megafloral change in the early and middle Paleocene in the Williston Basin, North Dakota, USA. *Palaeogeography, Palaeoclimatology, Palaeoecology* **298**, 224-234.
69. **Corbett SL, Manchester SR. 2004.** Phytogeography and Fossil History of Ailanthus (Simaroubaceae). *International Journal of Plant Sciences* **165**(4), 671-690.
70. **Takahashi M, Crane PR, Ando H. 1999.** *Esgueiria futabensis* sp. nov., a new angiosperm flower from the Upper Cretaceous (lower Coniacian) of northeastern Honshu, Japan. *Paleontological Research* **3**, 81-87.

71. **Friis EM., Pedersen KR., Crane PR. 2006.** Cretaceous angiosperm flowers: Innovation and evolution in plant reproduction. *Palaeogeography, Palaeoclimatology, Palaeoecology* **232**(2-4), 251-293.
72. **Yazykova E. 2002.** Ammonite and inoceramid radiations after the Santonian–Campanian bioevent in Sakhalin, Far East Russia. *Lethaia* **35**, 51-60.
73. **Manchester SR, Kapgate DK, Wen J. 2013.** Oldest fruits of the grape family (Vitaceae) from the Late Cretaceous Deccan Cherts of India. *American Journal of Botany* **100**(9), 1849-1859.
74. **Schoene B, Samperton KM, Eddy MP., Keller G, Adatte T, Bowring SA, Khadri SFR., Gertsch B. 2015**. U-Pb geochronology of the Deccan Traps and relation to the end-Cretaceous mass extinction. *Science* **347**, 182-184.
75. **Hermsen EJ, Gandolfo MA, Nixon K.C, Crepet WL. 2003** *Divisestylus* gen. nov. (aff. Iteaceae), a fossil saxifrage from the Late Cretaceous of New Jersey, USA. *American Journal of Botany* **90**, 1371-1388.
76. **Magallón S, Herendeen PS., Crane PR. 2001**. Androdecidua endressii gen. et sp. nov., from the Late Cretaceous of Georgia (United States): Further Floral Diversity in Hamamelidoideae (Hamamelidaceae). *International Journal of Plant Sciences* **162**(4), 963-983.
77. **Sims HJ, Herendeen PS., Crane PR. 1998.** New Genus of Fossil Fagaceae from the Santonian (Late Cretaceous) of Central Georgia, U. S. A. *International Journal of Plant Sciences* **159**(2), 391-404.
78. **Manchester SR, Donoghue MJ. 1995.** Winged fruits of Linnaeeae (Caprifoliaceae) in the Tertiary of western North America: *Diplodipelta* gen. nov. *International Journal of Plant Sciences* **156**(5), 709-722.
79. **Bell CD, Donoghue MJ. 2005.** Dating the dipsacales: Comparing models, genes, and evolutionary implications. *American Journal of Botany* **92**(2), 284-296.
80. **Evanoff E, McIntosh WC, Murphey PC. 2001.** Stratigraphic summary and 40Ar/39Ar geochronology of the Florissant Formation, Colorado. In *Fossil flora and stratigraphy of the florissant Formation, Colorado: Proceedings of the Denver Museum of Nature & Science, scr 4, no I, p 1-16* (eds. Evanoff E, Gregory-Wodzicki KM. Johnson KR), pp. 1-16. Denver, Denver Museum of Nature & Science.
81. **Prothero DR. 2008.** Magnetic stratigraphy of the Eocene-Oligocene floral transition in western North America. In Meyer, ll. W., and Smith, D. M., eds., Paleomology of the Upper Eocene Florissant Formation, Colorado. *Geological Society of America Special Paper 435*, 71-87.
82. **Martínez-Millán M. 2010.** Fossil record and age of the Asteridae. *Botanical Review* **76**, 83-135.
83. **Taylor D.W. 1989.** Select palynomorphs from the Middle Eocene Claiborne Formation, Tenn., (U.S.A.). *Review of Palaeobotany and Palynology* **58**(2-4), 111-128.
84. **Elsik WC. 1974.** Characteristic Eocene palynomorphs in the Gulf Coast, U.S.A. *Palaeontographica Abteilung B* **149**, 90-111.
85. **Zavada M, de Villiers S. 2000.** Pollen of the Asteraceae from the Paleocene-Eocene of South Africa. *Grana* **39**(1), 39-45.
86. **Wade BS., Pearson PN, Berggren WA, Pälike H. 2011.** Review and revision of Cenozoic tropical planktonic foraminiferal biostratigraphy and calibration to the geomagnetic polarity and astronomical time scale. *Earth-Science Reviews* **104**(1–3), 111-142.
87. **Mai DH. 1970.** Neue Arten aus tertiären Lorbeerwäldern in Mitteleuropa (Vorläufige Mitteilung). *Feddes Repertorium* **98**, 347-370.
88. **Mai DH. 1987.** Neue Früchte und Samen aus paläozänen Ablagerungen Mitteleuropas. *Feddes Repertorium* **98**, 197-229.
89. **Knobloch EN, Mai DH. 1986.** *Monographie der Früchte und Samen in der Kreide von Mitteleuropa*. Prague, Ústřední ústav geologický v Academii, nakl. Československé akademie věd.
90. **Knobloch E, Kvaček Z, Bůžek Č, Mai DH, Batten DJ. 1993.** Evolutionary significance of floristic changes in the Northern Hemisphere during the Late Cretaceous and Palaeogene, with particular reference to Central Europe. *Review of Palaeobotany and Palynology* **78**(1–2), 41-54.
91. **Berry EW. 1930.** Revision of the Lower Eocene Wilcox Flora of the southeastern states with descriptions of new species, chiefly from Tennessee and Kentucky. *United States Geological Survey Professional Paper* **156**, 1-196.
92. **Millan M, Crepet WL. 2014**. The fossil record of the Solanaceae revisited and revised—the fossil record of Rhamnaceae enhanced. *Botanical Review* **80**, 73-106.
93. **Crepet WL., Nixon KC, Daghlian CP. 2012.** Fossil Ericales from the Upper Cretceous of New Jersey. *International Journal of Plant Sciences* **174**, 572-584.
94. **Gandolfo MA., Nixon KC., Crepet WL. 1988.** *Tylerianthus crossmanensis* gen. et sp. nov. (Rosales) from the Upper Cretaceous of New Jersey. *American Journal of Botany* **85**, 376-386.
95. **Gandolfo MA, Nixon KC., Crepet WL 1998.** A new fossil flower from the Turonian of New Jersey: Dressiantha bicarpellata gen. et sp. nov. (Capparales). *American Journal of Botany* **85**(7), 964-974.

96. **Crepet WL., Nixon KC., Gandolfo MA. 2004.** Fossil evidence and phylogeny: The age of major angiosperm clades based on mesofossil and macrofossil evidence from Cretaceous deposits. *American Journal of Botany* **91**(10), 1666-1682.
97. **Drinnan AN, Crane PR, Friis EM, Pedersen KR. 1991.** Angiosperm flowers and tricolpate pollen of buxaceous affinity from the Potomac Group (mid-Cretaceous) of eastern North America. *American Journal of Botany* **78**, 153-176.
98. **Doyle JA. 2015.** Recognising angiosperm clades in the Early Cretaceous fossil record. *Historical Biology* **27**(3-4), 414-429.
99. **Hochuli PA., Heimhofer U, Weissert H. 2006.** Timing of early angiosperm radiation: recalibrating the classical succession. *Journal of the Geological Society* **163**, 587-594.
100. **Crane PR, Pedersen KR, Friis EM, Drinnan AN. 1993**. Early Cretaceous (early to middleAlbian) platanoid inflorescences associated with Sapindopsis leaves from the Potomac Group of eastern North America. *Systematic Botany* **18**, 328-344.
101. **Jacques FMB., Wang W, CORD, Li H-L., Zhou Z-K., Chen Z-D. 2010.** Integrating fossils in a molecular-based phylogeny and testing them as calibration points for divergence time estimates in Menispermaceae. *Journal Systematics and Evolution* **49**, 25-49.
102. **Pacltová B. 1981**. The evolution and distribution of *Normapolles* pollen during the Cenophytic. *Review of Palaeobotany & Palynology* **35**, 175-208.
103. **Knobloch E. 1985.** Paläobotanisch-biostratigraphische Charakteristik der Klikov-Schichtenfolge (Oberturon–Santon) in Südböhmen. *Sborník geologických věd, Geologie* **40**, 101–145.
104. **Doyle JA, Endress PK, Upchurch GR. Jr. 2008.** Early Cretaceous monocots: a phylogenetic evaluation. *Sbornik Narodniho Muzea v Praze Rada B Prirodni Vedy* **64**(2-4), 59-87.
105. **Hughes NF. 1994.** *The enigma of angiosperm origins*. Cambridge, Cambridge University Press.
106. **Hertweck KL, Kinney MS, Stuart SA, Maurin O, Mathews S, Chase MW, Gandolfo MA, Pires JC. 2015.** Phylogenetics, divergence times and diversification from three genomic partitions in monocots. *Botanical Journal of the Linnean Society* **178**(3), 375-393.
107. **Friis EM. 1988.** *Spirematospermum chandlerae* sp. nov., an extinct species of Zingiberacea from the North American Cretaceous. *Tertiary Research* **9**, 7-12.
108. **Christopher RA, Prowell DC 2010.** A palynological biozonation for the uppermost Santonian and Campanian Stages (Upper Cretaceous) of South Carolina, USA. *Cretaceous Research* **31**(2), 101-129.
109. **Berry EW. 1914.** The Upper Cretaceous and Eocene floras of South Carolina and Georgia. *United States Geological Survey Professional Paper* **84**, 1-200.
110. **Iles WJD, Smith SY, Gandolfo MA, Graham SW. 2015.** Monocot fossils suitable for molecular dating analyses. *Botanical Journal of the Linnean Society* **178**(3), 346-374.
111. **Sohl NF, Owens JP. 1991.** Cretaceous stratigraphy of the Carolina Coastal Plain. In *The Geology of the Carolinas: Knoxville* (eds. Horton J.W., Jr., Zullo V.A.), pp. 191-220, University of Tennessee Press.
112. **Campbell BG, Gohn GS. 1994.** Stratigraphic framework for geologic and geohydrologic studies of the subsurface Cretaceous section near Charleston, South Carolina. *United States Geological Survey Map MF-2273*, 1-11.
113. **Habib D, Miller JA. 1989.** Dinoflagellate species and organic facies evidence of marine transgression and regression in the atlantic coastal plain. *Palaeogeography, Palaeoclimatology, Palaeoecology* **74**(1–2), 23-47.
114. **Ramirez SR, Gravendeel B, Singer RB., Marshall CR., Pierce NE. 2007**. Dating the origin of the Orchidaceae from a fossil orchid with its pollinator. *Nature* **448**(7157), 1042-1045.
115. **Iturralde-Vinent MA., MacPhee RDE. 1996.** Age and paleogeographical origin of Dominican Amber. *Science* **273**, 1850-1852.
116. **Conran JG., Bannister JM, Mildenhall DC, Lee DE, Chacon J, Renner SS. 2014.** Leaf fossils of Luzuriaga and a monocot flower with in situ pollen of Liliacidites contortus Mildenh. & Bannister sp. nov. (Alstroemeriaceae) from the Early Miocene. *Am J Bot* **101**(1), 141-155
117. **Lindqvist JK, Lee DE. 2009.** High-frequency paleoclimate signals from Foulden Maar, Waipiata Volcanic Field, southern New Zealand: an early Miocene varved lacustrine diatomite deposit. *Sedimentary Geology* **222**, 98-110.
118. **Mildenhall DC., Kennedy EM, Lee DE, Kaulfuss U, Bannister JM, Fox B, Conran JG. 2014.** Palynology of the early Miocene Foulden Maar, Otago, New Zealand: Diversity following destruction. *Review of Palaeobotany and Palynology* **204**(0), 27-42.
119. **Cooper RA. 2004.** The New Zealand geological timescale. *Institute of Geological and Nuclear Sciences Monograph* **22**, 1-284.
120. **Hilgen FJ., Lourens LJ, Van Dam JA. 2012.** The Neogene Period. In *The Geologic Times Scale 2012* (eds. Gradstein F.M., Ogg J.G., Schmitz M., Ogg G.), pp. 923-978, Elsevier.

121. **Gandolfo MA., Nixon K.C, Crepet WL. 2002.** Triuridaceae fossil flowers from the Upper Cretaceous of New Jersey. *American Journal of Botany* **89**(12), 1940-1957.
122. **Friis EM, Pedersen KR, Crane PR. 2004.** Araceae from the Early Cretaceous of Portugal: Evidence on the emergence of monocotyledons. *Proceedings of the National Academy of Sciences of the United States of America* **101**(47), 16565-16570.
123. **Friis EM, Pedersen KR, Crane PR. 2010.** Diversity in obscurity: fossil flowers and the early history of angiosperms. *Philosophical Transactions of the Royal Society B: Biological Sciences* **365**, 369-382.
124. **Dinis JL, Rey J, Cunha PP, Callapez P, dos Reis RP. 2008.** Stratigraphy and allogenic controls of the western Portugal Cretaceous: an updated synthesis. *Cretaceous Research* **29**(5-6), 772-780.
125. **Berthou PY. 1973.** Le Cénomanien de l'Estremadure portugaise. *Memória, Serviços Geológicos de Portugal, Lisboa, NS* **23**, 169.
126. **Berthou PY. 1984.** Résumé synthetique de la stratigraphie et de la paléogéographie du Cretacé moyen et supérieur du bassin occidental portugais. *Geonovas* **7**, 99-120.
127. **Riley MG., Stockey RA. 2004**. *Cardstonia tolmanii* gen. et sp. nov. (Limnocharitaceae) from the Upper Cretaceous of Alberta, Canada. *International Journal of Plant Sciences* **165**(5), 897-916.
128. **Eberth DA., Braman DR. 2012.** A revised stratigraphy and depositional history for the Horseshoe Canyon Formation (Upper Cretaceous), southern Alberta plains. *Canadian Journal of Earth Sciences* **49**, 1053-1086.
129. **Bogner J, Johnson KR, Kvacek Z, Upchurch Jr. GR. 2007.** New fossil leaves of Araceae from the Late Cretaceous and Paleogene of western North America. *Zitteliana* **A47**, 133-147.
130. **Kvaček J, Herman AB. 2004.** Monocotyledons from the Early Campanian (Cretaceous) of Grünbach, Lower Austria. *Review of Palaeobotany and Palynology* **128**(3–4), 323-353.
131. **Nauheimer L, Metzler D, Renner SS. 2012.** Global history of the ancient monocot family Araceae inferred with models accounting for past continental positions and previous ranges based on fossils. *New Phytologist* **195**(4), 938-950.
132. **Herman AB, Kvaček J. 2010**. *Late Cretaceous Grünbach Flora of Austria*. Vienna, Naturhistorisches Museum Wien.
133. **Friis EM, Eklund H, Pedersen KR., Crane PR. 1994.** *Virginianthus calycanthoides* gen. et sp. nov.—A calycanthaceous flower from the Potomac Group (Early Cretaceous) of eastern North America. *International Journal of Plant Sciences* **155**, 772-785.
134. **Doyle JA., Endress PK. 2010.** Integrating Early Cretaceous fossils into the phylogeny of living angiosperms: Magnoliidae and eudicots. *Journal of Systematics and Evolution* **48**(1), 1-35.
135. **von Balthazar M, Crane PR, Pedersen KR, Friis EM. 2011.** New flowers of Laurales from the Early Cretaceous (Early to Middle Albian) of eastern North America. In *Flowers on the tree of life* (eds. Wanntorp L., Ronse De Craene L.P.), pp. 49–87. Cambridge Cambridge University Press.
136. **Doyle JA, Robbins EI. 1977.** Angiosperm pollen zonation of the continental Cretaceous of the Atlantic coastal plain and its application to deep wells in the Salisbury Embayment. *Palynology* (1), 43-78.
137. Mohr B.A.R., Bernardes-de-Oliveira M.E.C. 2004 Endressinia brasiliana, a magnolialean angiosperm from the lower Cretaceous Crato Formation (Brazil). *International Journal of Plant Sciences* **165**(6), 1121-1133.
138. **Mohr BAR, Coiffard C, Bernardes-de-Oliveira MEC. 2013.** Schenkeriphyllum glanduliferum, a new magnolialean angiosperm from the Early Cretaceous of Northern Gondwana and its relationships to fossil and modern Magnoliales. *Review of Palaeobotany and Palynology* **189**, 57-72.
139. **Sauquet H, Doyle JA, Scharaschkin T, Borsch T, Hilu K.W, Chatrou L.W, Le Thomas A. 2003.** Phylogenetic analysis of Magnoliales and Myristicaceae based on multiple data sets: implications for character evolution. *Botanical Journal of the Linnean Society* **142**, 125-186.
140. **Batten DJ. 2007.** Spores and pollen from the Crato Formation: biostratigraphic and palaeoenvironmental implications. In *The Crato fossil beds of Brazil - window into an ancient world* (eds. Martill D.M., Bechly G., Loveridge R.F.), pp. 566-573. Cambridge, Cambridge University Press.
141. **Martill DM. 2007.** The age of the Cretaceous Santana Formation fossil Konservat Lagerstatten of north-east Brazil: a historical review and an appraisal of the biochronostratigraphic utility of its palaeobiota. *Cretaceous Research* **28**(6), 895-920.
142. **Heimhofer U, Hochuli P-A. 2010.** Early Cretaceous angiosperm pollen from a low-latitude succession (Araripe Basin, NE Brazil). *Review of Palaeobotany & Palynology* **161**(3-4), 105-126.
143. **Friis EM., Pedersen KR., Crane PR. 1995.** *Appomattoxia ancistrophora* gen et sp nov, a new Cretaceous plant with similarities to Circaeaster and extant Magnoliidae. *American Journal of Botany* **82**(7), 933-943.
144. **Friis E.M, Pedersen KR., Crane PR. 2010.** Cretaceous diversification of angiosperms in the western part of the Iberian Peninsula. *Review of Palaeobotany and Palynology* **162**(3), 341-361.

145. **Smith SY, Stockey A. 2007.** Establishing a fossil record for the perianthless Piperales: *Saururus tuckerae* sp. nov. (Saururaaceae) from the Middle Eocene Princeton Chert. *American Journal of Botany* **94**, 1642–1657.
146. **Rouse GE, Mathews W. 1961,** Radioactive dating of Tertiary plant-bearing deposits. *Science* **133**, 1079-1080.
147. **Mathews WH. 1964.** Potassium-argon age determinations of Cenozoic volcanic rocks from British Columbia. *Geological Society of America Bulletin* **75**, 465-468.
148. **Hills LV, Baadsgaard H. 1967.** Potassium-argon dating of some Lower Tertiary strata in British Columbia. *Bulletin of Canadian Petroleum Geology* **15**, 138-149.
149. **Read PB. 2000.** Geology and industrial minerals of the Tertiary basins, south-central British Columbia. *British Columbia Geological Survey Geo-File* **2000**(3).
150. **Moss P.T, Greenwood DR., Archibald SB. 2005.** Regional and local vegetation community dynamics of the Eocene Okanagan Highlands (British Columbia – Washington State) from palynology. *Canadian Journal of Earth Sciences* **42**, 187-204.
151. **Hughes NF, Drewry G, Laing J.F. 1979.** Barremian earliest angiosperm pollen. *Palaeontology* **22**, 513-535.
152. **Friis EM., Pedersen KR., Crane PR. 2000.** Fossil floral structures of a basal angiosperm with monocolpate, reticulate-acolumellate pollen from the Early Cretaceous of Portugal. *Grana* **39**, 226–239.
153. **Doyle JA., Endress PK. 2014.** Integrating Early Cretaceous Fossils into the Phylogeny of Living Angiosperms: ANITA Lines and Relatives of Chloranthaceae. *International Journal of Plant Sciences* **175**(5), 555-600.
154. **Rocha RB., Manuppella G, Mouterde R, Ruget C, Zbyszewski G. 1981.** *Carta geológica de Portugal na escala de 1/50 00. Notícia explicativa da folha 19-C Figueria da Foz*. Lisboa, Serviços Geológicos de Portugal.
155. **Manuppella G, Telles Antunes M, Costa Almedia CA, Azeredo AC, Barbosa B, Cardosa JL., Crispim JA., Duarte LV., Henriques MH., Martins LT., et al. 2000.** *Carta geoógica de Portugal na escala de 1:50 000. Notícia explicativa da folha 27-A (Vila Nova de Ourém)*. Lisboa, Instituto Geológico e Mineiro; 156 p.
156. **Dinis JL. 2001.** Definicão da Formacão da Figueira da Foz-Aptiano a Cenomaniano do sector central da margem oesteibé rica. *Comunicações Instituto Geológico e Mineiro* **88**, 127– 160.
157. **Callapez P. 2003.** The Cenomanian-Turonian transition in West Central Portugal: ammonites and biostratigraphy. *Ciências da Terra* **15**, 53-70.
158. **Frumin S, Friis EM. 1999.** Magnoliid reproductive organs from the Cenomanian-Turonian of north-western Kazakhstan: Magnoliaceae and Illiciaceae. *Plant Systematics and Evolution* **216**(3-4), 265-288.
159. **Levina AP, Zhelezko VI, Leiptsig AB, Papulov GN, Ponomarenko ZK, Paskar ZS. 1990.** The Sokolov and Sarbay ironmine quarries. In *Upper Cretaceous deposits of the southern Transural (The region of the upper current of the Tobol River)* (eds. Papulov G.N., Zhelezko V.I., Levina A.R.), pp. 46-58. Sverdlovsk, Akademia Nauk SSSR.
160. **Korchagin OA, Bragina LG., Bragin NY. 2012.** Planktonic foraminifers and radiolarians from the Coniacian-santonian deposits of the Mt. Ak-Kaya, Crimean Mountains, Ukraine. *Stratigraphy and Geological Correlation* **20**(1), 73-96.
161. **Mohr BAR., Bernardes-De-Oliveira MEC, Taylor DW. 2008.** Pluricarpellatia, a nymphaealean angiosperm from the Lower Cretaceous of northern Gondwana (Crato Formation, Brazil). *Taxon* **57**(4), 1147-1158.
162. **Taylor DW., Brenner GJ., Basha SH. 2008.** Scutifolium jordanicum gen. et sp nov (Cabombaceae), an aquatic fossil plant from the Lower Cretaceous of Jordan, and the relationships of related leaf fossils to living genera. *American Journal of Botany* **95**(3), 340-352.
163. **Friis E.M, Pedersen KR., Crane PR. 2001.** Fossil evidence of water lilies (Nymphaeales) in the Early Cretaceous. *Nature* **410**(6826), 357-360.
164. **Friis EM, Pedersen KR, Von Balthazar M, Grimm G.W, Crane PR. 2009**. Monetianthus mirus gen. et sp. nov., a nymphaealean flowers from the Early Cretaceous of Portugal. *International Journal of Plant Science* **170**, 1086-1101.
165. **Smith SA., Beaulieu JM., Donoghue MJ. 2010.** An uncorrelated relaxed-clock analysis suggests an earlier origin for flowering plants. *Proceedings of the National Academy of Sciences* **107**(13), 5897-5902.
166. **Zanne AE., Tank DC., Cornwell WK, Eastman JM., Smith SA., FitzJohn RG, McGlinn DJ, O'Meara BC, Moles AT., Reich PB., et al. 2014**. Three keys to the radiation of angiosperms into freezing environments. *Nature* **506**(7486), 89-92.

167. **Amireh BS., Jarrar G, Henjes-Kunst F, Schneider W. 1998**. K-Ar dating, X-ray diffractometry, optical and scanning electron microscopy of glauconites from the Early Cretaceous Kurnub. *Geological Journal* **33**, 49-65.
168. **Wieland GW. 1935.** The Cerro Cuadrado petrified forest. *Carnegie Institution of Washington Publication* **449**, 1-183.
169. **Calder MG. 1953.** A coniferous petrified forest in Patagonia. *Bulletin of the British Museum (Natural History): Geology* **2**, 99-138.
170. **Stockey RA. 1975.** Seeds and embryos of *Araucaria mirabilis*. *American Journal of Botany* **62**(8), 856-868.
171. **Stockey RA. 1978.** Reproductive biology of Cerro Cuadrado fossil conifers: Ontogeny and reproductive strategies in *Araucaria mirabilis* (Spegazzini) Windhausen. *Palaeontographica Abteilung B* **166**, 1-15.
172. **Wilde MH., Eames AJ. 1948.** The ovule and seed of *Araucaria badwillii* with discussion of the taxonomy of the genus. 1. Morphology. *Annals of botany* **12**(47), 311-&.
173. **Spalleti L, Iñiguez Rodríguez AM, Masón M. 1982.** Edades radimétricas de piroclastitas y volvanitas del Grupo Bahía Laura, Gran Bajo de San Julián, Santa Cruz. *Revista de la Asociación Geológica Argentina* **37**, 483-485.
174. **Florin R. 1951.** Evolution in cordaites and conifers. *Acta Horti Bergiani* **15**, 285-388.
175. Yao X.L., Taylor T.N., Taylor E.L. 1997 A taxodiaceous seed cone from the Triassic of Antarctica. *American Journal of Botany* **84**(3), 343-354.
176. **Axsmith BJ., Taylor TN, Taylor EL. 1998.** Anatomically preserved leaves of the conifer Notophytum krauselii (Podocarpaceae) from the Triassic of Antarctica. *American Journal of Botany* **85**(5), 704-713.
177. **Krassilov VA. 1986.** New floral structure from the Lower Cretaceous of Lake Baikal Area. *Review of Palaeobotany and Palynology* **47**, 9-16.
178. **Rydin C, Pedersen KR, Friis EM. 2004.** On the evolutionary history of *Ephedra*: Cretaceous fossils and extant molecules. *Proceedings of the National Academy of Sciences, USA* **101**, 16571-16576.
179. **Friis EM., Pedersen KR., Crane PR. 2009.** Early Cretaceous mesofossils from Portugal and eastern North America related to the Bennettitales-Erdtmanithecales-Gnetales group. *American Journal of Botany* **96**, 252-283.
180. **Godefroit P. 2012**. *Bernissart dinosaurs and Early Cretaceous terrestrial ecosystems*. Bloomington, IN, Indiana University Press; 648 p.
181. **Vakhrameev V, Kotova I. 1977.** Ancient angiosperms and accompanying plants from the Lower Cretaceous of Transbaikalia. *Paleontological Journal* **4**, 487-495.
182. **Vakhrameev V. 1991**. *Jurassic and Cretaceous floras and climates of the Earth*. Cambridge, Cambridge University Press; 318 p.
183. **Chen P, Wang Q, Zhang H, Cao M, Li W, Wu S, Shen Y. 2005.** Jianshangou Bed of the Yixian Formation in West Liaoning, China. *Science in China Series D: Earth Sciences* **48**(3), 298-312.
184. **Dettmann ME., Thomson MRA. 1987.** Cretaceous palynomorphs from the James-Ross Island area, Antarctica - a pilot-study. *British Antarctic Survey Bulletin* **77**, 13-59.
185. **Gao Z, Thomas BA. 1989.** A review of fossil cycad megasporophylls, with new evidence of Crossozamia pomel and its associated leaves from the lower Permian of Taiyuan, China. *Review of Palaeobotany and Palynology* **60**, 205-223.
186. **Nagalingum NS, Marshall CR, Quental TB, Rai HS, Little DP., Mathews S. 2011.** Recent synchronous radiation of a living fossil. *Science* **334**(6057), 796-799.
187. **Hermsen EJ., Taylor TN., Taylor EL, Stevenson DW. 2006.** Cataphylls of the Middle Triassic cycad Antarcticycas schopfii and new insights into cycad evolution. *American Journal of Botany* **93**(5), 724-738.
188. **Wang J. 2010.** Late Paleozoic macrofloral assemblages from Weibei Coalfield, with reference to vegetational change through the Late Paleozoic Ice-age in the North China Block. *International Journal of Coal Geology* **83**(2-3), 292-317.
189. **Henderson CM, Gradstein FM., Hammer O. 2012.** The Permian Period. In *The geologic timescale 2012* (eds. Gradstein F.M., Ogg J.G., Schmitz M., Ogg G.), pp. 653-679, Elsevier.
190. **Wellman CH, Gensel PG, Taylor WA. 2009.** Spore wall ultrastructure in the early lycopsid *Leclercqia* (Protolepidodenrales) from the Lower Devonian of North America: evidence for a fundamental division in the lycopsids. *American Journal of Botany* **96**(10), 1849-1860.
191. **Meyer-Berthaud B, Fairon-Demaret M, Steemans P, Talent J, Gerrienne P. 2003**. The plant Leclercqia (Lycopsida) in Gondwana: implications for reconstructing Middle Devonian palaeogeography. *Geological Magazine* **140**(2), 119-130.
